# Liquid biopsy in cancer: current status, challenges and future prospects

**DOI:** 10.1038/s41392-024-02021-w

**Published:** 2024-12-02

**Authors:** Liwei Ma, Huiling Guo, Yunxiang Zhao, Zhibo Liu, Chenran Wang, Jiahao Bu, Ting Sun, Jianwei Wei

**Affiliations:** 1 Department of Clinical Laboratory, The First Affiliated Hospital of Zhengzhou University, Zhengzhou, Henan China; 2 Key Clinical Laboratory of Henan province, Zhengzhou, Henan China; 3 Department of Neurosurgery, The First Affiliated Hospital of Zhengzhou University, Zhengzhou, Henan China

**Keywords:** Cancer, Cell biology

## Abstract

Cancer has a high mortality rate across the globe, and tissue biopsy remains the gold standard for tumor diagnosis due to its high level of laboratory standardization, good consistency of results, relatively stable samples, and high accuracy of results. However, there are still many limitations and drawbacks in the application of tissue biopsy in tumor. The emergence of liquid biopsy provides new ideas for early diagnosis and prognosis of tumor. Compared with tissue biopsy, liquid biopsy has many advantages in the diagnosis and treatment of various types of cancer, including non-invasive, quickly and so on. Currently, the application of liquid biopsy in tumor detection has received widely attention. It is now undergoing rapid progress, and it holds significant potential for future applications. Around now, liquid biopsies encompass several components such as circulating tumor cells, circulating tumor DNA, exosomes, microRNA, circulating RNA, tumor platelets, and tumor endothelial cells. In addition, advances in the identification of liquid biopsy indicators have significantly enhanced the possibility of utilizing liquid biopsies in clinical settings. In this review, we will discuss the application, advantages and challenges of liquid biopsy in some common tumors from the perspective of diverse systems of tumors, and look forward to its future development prospects in the field of cancer diagnosis and treatment.

## Introduction

Cancer is the second major cause of death in the world and is a major worldwide public health problem. Early detection and appropriate therapy are crucial for cancer patients to enhance their prognosis and enhance their chances of survival.^1^ Currently, the golden standard for tumor diagnosis is still tissue biopsy. Although tissue biopsy can definitively diagnose tumors and their subtypes, tissue biopsy is difficult to collect and, as an invasive test, it is prone to cause damage to patients and is not convenient for continuous monitoring of the disease progression.^2^ As tumors are sometimes hard to detect early, it is difficult to use tissue biopsies to accurately detect tumors at an early stage in the diseases.

Liquid biopsy is a mini-invasive sample collection method that focuses on blood or body secretions for the detection of molecular alterations, tumor cells, and metabolites.^3,4^ Compared to tissue biopsies, liquid biopsies provide a role in early screening. Common specimens for liquid biopsy are blood and urine.^5^ Therefore, liquid biopsies are easier to perform than tissue biopsies and are virtually non-invasive to the patient,^5,6^ which makes liquid biopsies have the potential for continuous monitoring of tumor progression.^7^ Several molecular markers can be detected by liquid biopsy, such as circulating tumor cells (CTCs), circulating tumor DNA (ctDNA), tumor-derived extracellular vesicles (EVs), tumor-educated platelets (TEPs), and circulating free RNA (cfRNA).^7,8^ Currently, more studies focus on the detection of CTCs, ctDNA and exosomes. In this paper, we will introduce various liquid biopsy molecular markers and summarize the current applications of liquid biopsy in various tumor systems from different systems.

## The research history of liquid biopsy

**Fig. 1:**
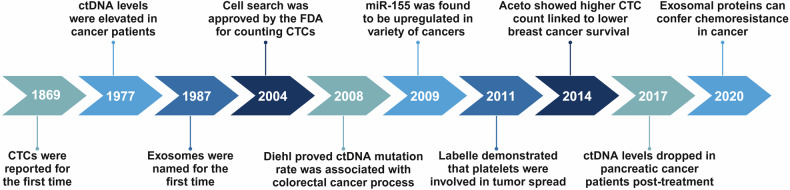
History of liquid biopsy. Timeline of the research history and milestone events of study on liquid biopsy. CTCs Circulating tumor cells, ctDNA Circulating tumor DNA, FDA Food and Drug Administration. Created with BioRender.com

The development of liquid biopsy has gone through four main phases: the period of scientific exploration (before the 1990s), the period of scientific development (1990s), the period of industrial growth (2000–2010), and the period of industrial outbreak (2010-present) (Fig. 1).

During the period of scientific exploration, several scholars have discovered the existence of CTCs, cfDNA and extracellular vehicles (EVs). In 1869, Australian physician Thomas Ashworth found cells similar to tumor cells in the blood of a recently deceased tumor patient.^9^ In 1948, Mandel and Metais made the groundbreaking discovery of the existence of unbound nucleic acid molecules in plasma.^10^ In 1967, Wolf obtained the first electron micrographs of EVs.^11^ In 1983, Stahl and Johnstone’s laboratory suggested that exosomes are discharged from EVs that had merged with the cell membrane through multivesicular structures.^12^ In addition, a study conducted by Leon et al. in 1977 revealed that levels of plasma free DNA were much elevated in individuals with tumors compared to those in the healthy population. This led to the hypothesis that free DNA is linked to the presence of tumors.^13^ In the period of scientific progress, CTC was initially isolated from blood in 1998 and was proven to correlate with pathologic staging, and it has only since been employed in the clinic.^14^ Additionally, in 1994, PCR was used to identify the first KRAS mutation in pancreatic cancer patients’ blood cfDNA, and the results were consistent with those found in tumor tissue.^15^ In 1996, Raposo provided evidence that EVs possess biological activity. It has been discovered that immune cells’ EVs can present antigens.^16^ Liquid biopsy indicators were discovered to be useful in the diagnosis of a variety of cancers during this time of industrial expansion. In patients with metastatic breast cancer, the quantity of CTCs prior to therapy was found to be an independent predictor of both overall survival and progression-free survival in 2005.^17^ Diehl F. et al. followed up on the ctDNA of 18 patients with bowel cancer in 2008 and used the BEAMing technique to identify hotspot mutations in genes like TP53, APC, KRAS, and PIK3CA. They discovered that the rate of ctDNA mutations changed over the course of treatment and that the trend of the change was positively correlated with both the tumor load and the CEA concentration.^18^ Several liquid biopsy markers were included into oncology guidelines and given the go-ahead for clinical use during the industrial boom. The use of ctDNA to identify EGFR mutations for concurrent Erizar diagnosis was authorized by the European Medicines Agency (EMA) in 2014, hence initiating the official clinical usage of ctDNA. According to the 2015 Chinese Expert Consensus on Blood EGFR Gene Mutation Testing in Non-Small Cell Lung Cancer (NSCLC), which was published in the Chinese Medical Journal, ctDNA from the blood (plasma) specimen can be used for evaluation if the tumor specimen cannot be assessed for EGFR gene status.^19^ And the use of CTC testing for prognostic assessment in breast cancer was addressed by AJCC recommendations in 2018.^20^ In 2019, CTC was included into the 2019 CSCO Breast Cancer Treatment Guidelines.^21^ More recently, in 2023, CTC entered the Chinese Technical Guidelines for Integrated Cancer Therapy (CACA).

## Molecular markers of liquid biopsy

**Fig. 2:**
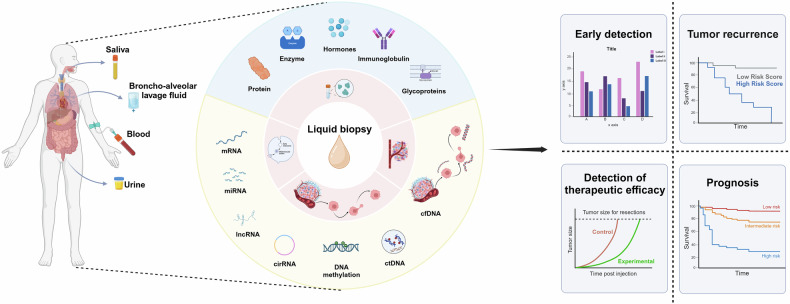
Flowchart of applying liquid biopsy in cancers. Applications of liquid biopsies and types of biomarkers for liquid biopsies. Created with BioRender.com

In this section we focus on several liquid biopsy biomarkers currently in use (Fig. 2). And summarizes the comparison of different liquid biopsy markers (Tables 1–5).

**Table 1:** Separation and detection of CTCs

Cellular Assay Methods	Cell Characterization/Detection Principles	Advantages	Disadvantages	Reference
Automatic scanning of fluorescence microscopes	Telomerase-specific replication-selective adenovirus expressing GFP (green fluorescent protein)	The assay is simple, detects a wide range of tumor cells, and does not require CTC enrichment.	Lack of large sample tests, relatively time-consuming and complicated procedures.	^93^
CTC-iChip	NA	No need to enrich CTC	Higher cost and only 39% CTC detection rate	^94^
Subtractive enrichment (SE) and immunostaining-FISH (iFISH)	Polyploid with chromosome 8	Effective removal of leukocytes and erythrocytes, less loss of CTCs, significantly less time-consuming than traditional fish methods, and simultaneous detection of protein expression of multiple tumor markers specifically on CTCs	The development of relevant techniques is still in its infancy, and acute infectious lesions and benign space-occupying lesions may lead to false positives	^94,95^
Parsortix PC1 system	Physical method detection, microfluidic devices	Several multi-center clinical studies have demonstrated its ability to capture and collect CTCs	Processing is quite slow.	^96^
Cellsearch	EpCAM protein, immunomagnetic enrichment, fluorescent labeling	Considered the gold standard for CTC detection	Detection of EpCAM+ cells only, not applicable for some CTCs lacking EpCAM expression, e.g., GBM	^97^

**Table 2:** ctDNA detection techniques

Detection Methods	Detection Principle	Role	Advantages	Disadvantages	Reference
ddPCR	DNA amplification, sample microtitration and reading of the starting concentration of target molecules by fluorescence signaling	Detection of single nucleotide variants, quantification of nucleotides	High sensitivity and specificity, relatively low cost for specific DNA detection, short time to achieve absolute quantification of target molecules, suitable for long-term monitoring of patients with known mutations	It cannot process a large amount of sequence information at the same time and can only amplify known sequences.	^98^
NGS	Sequence information is read after DNA amplification using signals emitted at base insertion into the DNA strand with the help of chemical markers	Whole exome sequencing (WES) as well as whole genome sequencing (WGS), detection of nucleotide variants	Large amount of sequence information can be processed at the same time, detection time is not long, suitable for patient screening of unknown mutations, lower cost compared to ddPCR for large amount of DNA detection	The sensitivity and specificity are not as good as ddPCR.	^99,100^

**Table 3:** Exosome detection techniques

Technology	Mechanisms	Advantage	Disadvantage	Reference
differential centrifugation	Separation of substances of different sizes and densities by centrifugal force	The extraction method is simple, widely applicable, does not introduce additional markers, can handle a certain dose of sample, low cost, and does not contaminate exosomes	Cumbersome, time-consuming and labor-intensive, and the structure of the exosome may be destroyed.	^101^
filtration	Utilizes ultrafiltration membranes to selectively allow molecules or particles smaller than the membrane pore size to pass through.	High exosome recovery, simple handling, no introduction of additional markers	Poor ability to separate exosomes, time consuming, contamination of exosomes	^101^
polymer precipitation	and exosomes bind to each other, forming a complex, and then exosomes are separated by centrifugation	High recovery rate, easy to operate, can handle a large number of samples	Contamination of exosomes, low recovery purity, easy to damage the integrity of the exosome membrane	^101^
immunomagnetic bead method	Magnetic separation of exosomes using magnetism after specific binding of antibody-coated magnetic beads to exosome markers	High specificity, high purity of isolated exosomes, average difficulty in getting started	Takes time, costly, can’t handle large numbers of samples, tends to change exosome function	^101^
Chromatography (volumetric exclusion chromatography)	Separation of exosomes by continuous movement in different phases, taking advantage of the differences in the partitioning, adsorption and desorption properties of the components of the mixture between the stationary and mobile phases	High recovery rate, high purity, short time-consumption, low cost, simple operation, not easy to change the function of exosomes, no need for a large number of samples to isolate exosomes, can handle a large number of samples	Exosomes are diluted during the isolation process and may need to be subsequently concentrated, with potential contaminants that may contaminate the sample	^101^
microfluidic	Isolation of exosomes by methods such as exosome size or surface-specific markers	Good ability to isolate exosomes, high recovery of exosomes, no need for a large number of samples to isolate exosomes, fast isolation speeds	Costly to operate and maintain, requires specialized equipment, training prior to use, not able to process large quantities of samples	^57,101^

**Table 4:** RNA detection methods

Technology	Mechanisms	Advantage	Disadvantage	Reference
RNA fluorescence in situ hybridization (RNA-FISH)	Hybridization signals were observed using fluorescence microscopy after binding to the target RNA with a fluorescent probe complementary to the target RNA sequence	High sensitivity and specificity, multi-color detection, relatively simple and time-consuming operation, tissue morphology can be maintained for detection	High sample requirements, need to ensure RNA integrity, need specialized equipment and probes, high cost, limited accuracy of quantification	^102^
RT-PCR (reverse transcription PCR)	PCR amplification after reverse transcription of RNA to cDNA	It is highly sensitive and specific, suitable for the detection of a wide range of RNAs, less time consuming and more accurate.	Complexity of operation, susceptibility to contamination by foreign products, expensive equipment and reagents	^103^
Northern Blotting	Complexity of operation, susceptibility to contamination by foreign products, expensive equipment and reagents	High sensitivity and specificity, quantitative detection of RNA compared to RNA-FISH	Time-consuming, more complex operations, high sample requirements, need to ensure RNA integrity, need specialized equipment and probes, higher costs	^104^
in situ hybridization	The principle is similar to RNA-FISH, but labeled using markers such as radioisotopes, biotin, digoxin, etc., and finally visualized by radioactive autoradiography, immunohistochemistry, etc.	Both DNA and RNA can be detected at a moderate cost	Not as accurate as RNA-FISH, multiple hybridizations are not as simple as RNA-FISH, can only capture RNA from cells at a certain time point	^105^
RNA microarray	Hybridization of RNA by immobilizing a large number of probes on a microarray	High throughput, accurate quantification and good reproducibility.	Can only detect highly expressed RNAs and cannot cover the full range of RNAs, especially lncRNAs. cost is high and affected by experimental complexity.	^106^
RNA sequencing	Direct sequencing of RNA molecules using high-throughput sequencing technology	Detects all RNAs, capable of deep sequencing with high sensitivity and specificity	Costly, requires advance removal of rRNAs	^106^

**Table 5:** Comparison of different liquid biopsy markers

Form	ctDNA	CTCs	Exosome
source	Blood, urine, saliva, synovial fluid, cerebrospinal fluid, etc.	Blood, cerebrospinal fluid, urine, etc.	Blood, urine, cerebrospinal fluid, ascites, pleural fluid, etc.
scale	Nanoscale (DNA fragments)	cellular level	Nanoscale (40–150 nm)
information load	Can carry information on multiple genetic variants	Complete genetic information, including genome, transcriptome, epigenetic variation	Carrying proteins, RNA and many other biomolecules
clinical significance	Early screening, companion diagnosis, prognostic assessment, MRD testing	Prognostic assessment, drug sensitivity prediction, drug resistance mechanism studies	Early diagnosis, prognostic assessment, drug response monitoring
stability	Relatively low (short half-life)	high	High (phospholipid bilayer protection)
rarity	High (especially in early-stage tumors)	high	moderate (interference from other vesicles in body fluids)
heterogeneity	low	High (large variation between CTCs)	moderate
Difficulty of isolation and purification	moderate	High (not yet standardized)	High (technically complex)
background noise	Moderate (normal cfDNA interference)	low	Moderate (interference from other vesicles in body fluids)
Difficulty of standardization	moderate	high	high
technical difficulty	Moderate (relies on high-sensitivity detection technology)	High (complex enrichment, identification techniques)	Medium (dependent on specific detection techniques)
operating difficulty	low	High (multi-step operation)	moderate

## Circulating tumor cells (CTCs)

**Fig. 3:**
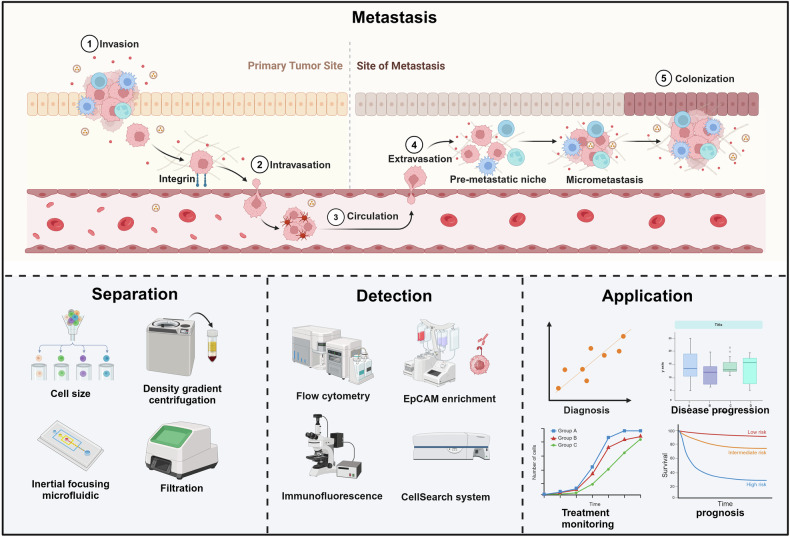
Liquid biopsy markers—CTCs. The metastasis, separation detection and application of CTCs. Created with BioRender.com

In 1869, Ashworth et al. first reported CTCs in the circulation of patients, which laid an important foundation for the study of CTCs. CTCs are cells released from primary and metastatic tumors that are shed into the blood or lymphatic vessels of cancer patients and circulate in the peripheral blood^22^ (Fig. 3). Although the proportion of CTCs in the blood is low, almost 1 CTCs is found per 1 million leukocytes, and most CTCs die in the peripheral blood in 1–2.5 h.^23,24^ However, in recent years, a large number of studies have demonstrated that the level of CTCs is associated with cancer development, especially playing an important role in the metastatic process of cancer,^25^ and these confirm that CTCs are an important biomarker. Therefore, CTCs have the potential to become an effective tool for cancer diagnosis, providing information for clinical decision-making and clinical research.^26,27^ A key challenge currently faced is how to isolate and collect CTCs more accurately, and the rapid development of technology has further facilitated the clinical application of CTCs.^28^ With technological advances and innovations, CTCs counts are associated with tumor status and higher accuracy. Studies have shown that higher levels of CTCs counts are associated with reduced progression-free survival and overall survival.^29,30^ For example, in 2014, Ramirez et al. demonstrated that in blood samples from breast cancer patients, an increased count of CTCs was found to be associated with a significant reduction in progression-free survival. As a result, the detection of CTCs has gained increasing attention as one of the important biomarkers for liquid biopsy. Due to the extremely low number of CTCs, it is high sensitivity advanced techniques to efficiently capture and detect CTCs that are necessary. Currently, methods used for the detection or isolation of CTCs are constantly being improved and have greatly increased in complexity and sensitivity.^31^ There are traditional methods such as density gradient centrifugation, inertial focusing, and filtration based on biophysical properties such as size, deformability, etc.^32^. There are also methods for the detection of cells by the expression of specific markers, epithelial cell adhesion molecule (EpCAM), vimentin, and N-cadherin, such as EpCAM enrichment, immunomagnetic separation, and microfluidic devices.^33^ Among them, the CellSearch® method is currently the only method authorized by the FDA to monitor the number of CTCs in blood samples.^34^ Even though these methods have a variety of shortcomings (Table 1), they have played a significant role in promoting research on the detection and clinical value of CTCs. CTCs, as an almost noninvasive test, will play an increasingly important value in the diagnosis, detection, and prognosis of tumors in the future.

## Circulating tumor DNA (ctDNA)

**Fig. 4:**
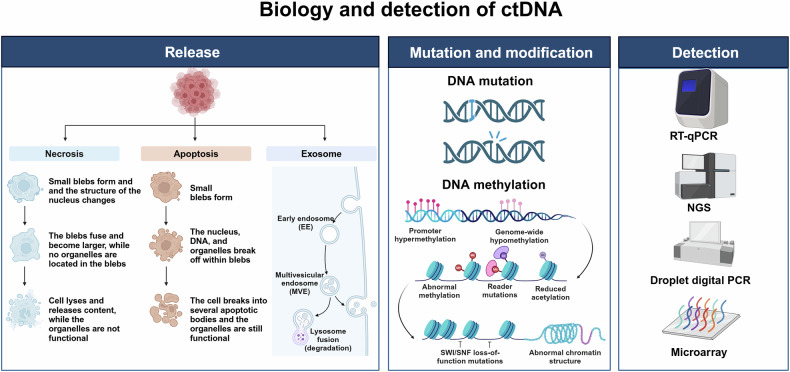
Liquid biopsy markers—ctDNA. CtDNA is usually actively secreted by tumor cells or released into the circulatory system during the apoptosis or necrosis of tumor cells. Mutations and methylation of ctDNA are often used as detection indicators. Created with BioRender.com

Circulating tumor DNA (ctDNA) can be extracted from the bloodstream and originates from the tumor. It is a type of circulating extracellular nucleic acids (cfDNA).^35^ CfDNA is primarily derived from normal leukocytes and stromal cells. However, in 1977, Leon et al. found that plasma-free DNA levels were significantly higher in patients with advanced tumors than in healthy individuals suggesting that cfDNA may also be derived from tumor cells.^13^ CtDNA only accounts for a small fraction of cfDNA, approximately 0.1–1.0% of its total^36^ (Fig. 4).

Similar to CTCs, ctDNA has traditionally been obtained from blood, but ctDNA can also be isolated by obtaining ascites, pleural fluid, urine, and cerebrospinal fluid (CSF). CfDNA is primarily derived from normal leukocytes and stromal cells, and ctDNA can dynamically respond to the state of the tumor at a given point in time. Compared with cfDNA, it has been shown that ctDNA base fragments in cancer patients are shorter than cfDNA, which is about 20–50 base pairs, making it less affected by intra-tumor heterogeneity.^37^ On the other hand, ctDNA has a shorter half-life, which is a prerequisite for its ability to be used as a real-time tumor biomarker, and it is these two characteristics of ctDNA that give it a distinct advantage when compared with traditional biopsy markers. The prognostic significance of ctDNA in cancer progression and its response to treatment has been described in recent years.^38,39^ It has been found that ctDNA levels are elevated in the serum of patients with pancreatic cancer (PC) and appear to decrease after treatment.^13^ In addition, the current clinical application often detects the mutation of target genes within ctDNA, for example, Diehl F and his team analyzed the serum ctDNA of 18 colorectal cancer patients and found hotspot mutated genes, such as APC, KRAS, TP53, and PIK3CA. And the mutation rate of ctDNA is related to its therapeutic process.^18^ Gene mutation can often trigger the imbalance of oncogenes and oncogenes, and then lead to cancer, so the mutation detection of ctDNA is of great significance for cancer detection. Abnormal DNA methylation also plays a key role in cancer development. In many tumors, an imbalance in DNA methylation usually precedes tumor formation and contributes to the early diagnosis of tumors.^40^ The detection of ctDNA has become increasingly sophisticated with technological advances, such as real-time quantitative polymerase chain reaction, digital droplet PCR (ddPCR), sanger sequencing, and next-generation sequencing (NGS).^41–43^ In the future, ctDNA assays will be widely used in new therapies to appropriately monitor the dynamics of tumor load and the cancer progression or prognosis.

## Exosomes

**Fig. 5:**
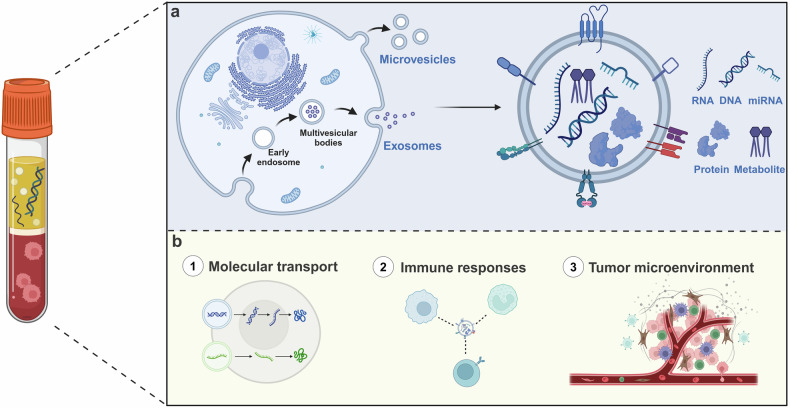
Liquid biopsy markers – exosome. **a** The formation process of exosomes and the main detection contents such as RNA, DNA, miRNA, proteins, and metabolite. **b** The role of exosome in tumor progression. Created with BioRender.com

In 1987, Johnstone first named the vesicles released by sheep reticulocytes as exosomes.^44^ Exosomes are a subtype of extracellular vesicles that originate from endosomes produced by trap buds in the membranes of multivesicular bodies and are released outside the cell after the fusion of multivesicular endosomes with the cell membrane^45^ (Fig. 5). The other two major subtypes of extracellular vesicles are microvesicles and apoptotic vesicles whose categorization is based primarily on size and cellular origin. The three main subtypes of exosomes have received much attention in recent years.^46^ Exosomes can be detected in blood, saliva, urine, and other fluids, engaging in a variety of biological processes such as molecular transport, intercellular communication, and immune responses. In addition, it has been found that exosomes are key components of the tumor microenvironment and play an important role in cancer progression.^47^ While exosomes have unique advantages in the field of liquid biopsy, on the one hand, they are well stabilized, and on the other hand, they are more representative in describing the information of tumor cells.^48^ In recent years, exosomal products, such as nucleic acids, proteins, lipids, and metabolites have gradually become a focus of research in the field of cancer, for example, exosomal non-coding RNAs (ncRNAs) have been shown to provide important reference value in the diagnosis and treatment of cancer patients. The upregulation of exosomes miR-1246, miR-4644, miR-3976, and miR-4306 can be used as highly sensitive biomarkers in prostate cancer patients.^49^ In addition, exosomal lncRNA H19 was found to be upregulated in serum expression in bladder cancer patients, suggesting that exosomal lncRNAs have a potential role as important diagnostic markers.^50^ Due to their unusually large variety and number, exosomal proteins have also received extensive attention in recent years.^51^ Exosomal proteins have a regulatory role in the formation of the cancer microenvironment, tumor progression, and metastasis.^52,53^ In addition, exosomal proteins can also mediate chemoresistance in cancer treatment, and a recent study showed that plasma gelatin (pGSN), an isoform of GSN protein secreted by chemoresistant ovarian cancer cells, can be delivered to exosomes and activate α5β1 integrin. This leads to an increase in hypoxia-inducible factor 1 subunit α, which in turn promotes chemoresistance and survival of ovarian cancer cells.^54^ In view of the fact that exosomes are one of the markers of a liquid biopsy and their important clinical applications, it is particularly important to isolate and detect them efficiently and accurately. In recent years, such approaches as Reverse Transcription-Polymerase Chain Reaction (RT-PCR), genome sequencing, and proteomics are often available for the detection of exosomal content.^55,56^ Techniques such as differential ultracentrifugation, size-based separation, immunomagnetic separation, and microfluidics are commonly used for exosome isolation.^57^ In the future, with the development of technology and multidisciplinary fusion, exosome, one of the markers of liquid biopsy, will be more closely integrated with clinical applications, especially cancer detection.

## Tumor educated-platelets

**Fig. 6:**
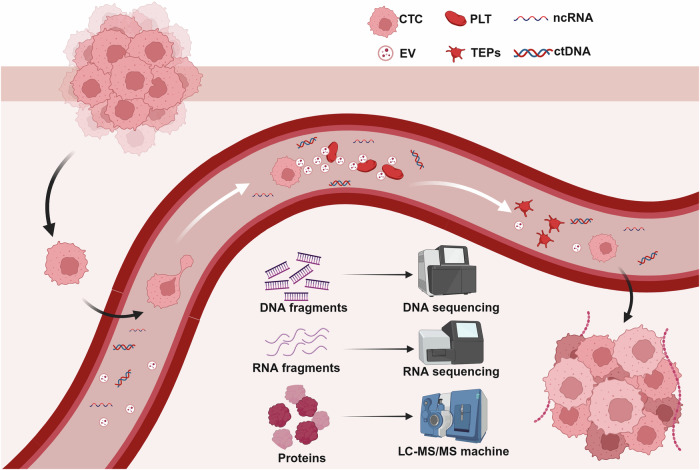
Liquid biopsy markers—TEPs. The formation process and the detection of TEPs. CTC circulating tumor cell, EV extracellular vehicle, PLT platelet, TEPs tumor educated-platelets. Created with BioRender.com

When it comes to platelets, what often first comes to mind is their hemostatic and thrombotic role, however, the fact is that platelets are gradually being recognized as mediators of malignant disease.^58^ As the second most abundant cell in the peripheral blood, they play a role in hematological processes, such as wound healing, atherosclerosis, vascular growth regulation, and angiogenesis.^59^ In the 1800s Reiss et al. first reported that high platelet counts were associated with malignancy and that host-tumor interactions activate the coagulation cascade in many types of cancers, and since then, more relevant evidence has suggested a link between platelet counts and cancer.^60,61^ It has been found that platelet deposition is positively correlated with mortality in patients with cancer, and it is considered to be the second most common cause of cancer deaths.^62^ In addition, there is a unique type of platelet that is often used as a biomarker for liquid biopsies and has received much attention in recent years. It is a type of platelet that is isolated from tumor patients but exhibits a different RNA and protein profile, named TEPs^63^ (Fig. 6). Studies have shown the involvement of TEPs in the progression and spread of a variety of solid tumors. Specifically spliced TEP RNA markers can provide specific information on tumor presence, location, and molecular features, but the exact mechanisms require further research.^64^ While there are no present clinical applications for TEPs, numerous studies have explored the potential clinical uses of TEPs, providing valuable insights. Tumor platelets exert a bidirectional influence, causing platelets to consistently absorb proteins, nucleic acids, vesicles, and granules from tumors. This process results in alterations to the RNA and protein expression profiles of the platelets.^65^ Platelets possess several advantages as a component of liquid biopsy. They exhibit stability and ease of collection, as they may be readily obtained through low-speed centrifugation. Furthermore, the genetic material contained within platelets is relatively durable.^66^ Due to the limited lifespan of platelets, the composition of TEP can accurately indicate the current condition of the tumor, allowing for real-time surveillance of the tumor. Further investigation is required to fully understand the precise mechanism, but the spliced TEP RNA markers have the potential to offer precise details regarding the presence, location, and molecular features of tumors.^64^ Present research on platelets in persons with tumors has primarily concentrated on mRNA and lncRNA. Numerous studies have demonstrated the capability of RNA sequencing analysis to distinguish between tumor patients and those who are in good health.^67^ In 2022, Ye et al. discovered four specific long-stranded non-coding RNA (lncRNA) markers associated with colorectal cancer (CRC) that are found in platelets. These markers include LNCAROD, SNHG20, LINC00534, and TSPOAP-AS1. The expression levels of these lncRNAs were markedly increased in both platelets and serum samples from individuals diagnosed with colorectal cancer. This finding strongly indicates that these lncRNAs hold promising diagnostic value.^68^ A gene expression database specifically designed for platelet-based disease research was established in 2022. We anticipate that this database will significantly enhance the investigation of platelet liquid biopsies.^69^ Currently, the understanding of the mechanisms involving platelet RNA is incomplete, and the use of TEPs for tumor treatment is still in the conceptual phase, necessitating further extensive research.

## miRNA and lncRNA

**Fig. 7:**
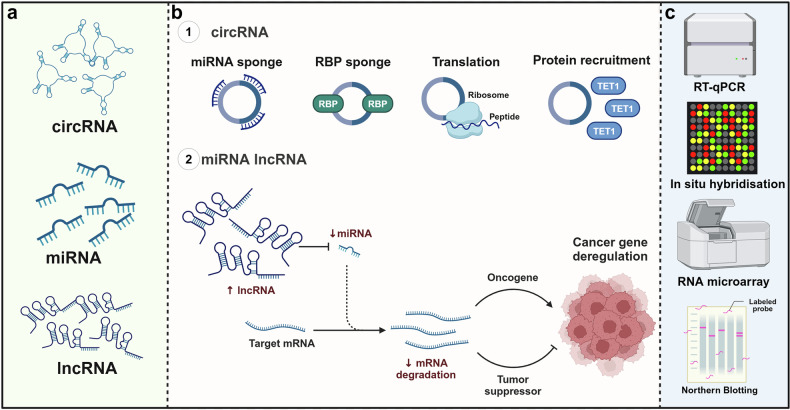
Liquid biopsy markers—RNA. **a** Types of ncRNA. **b** The role of ncRNA. **c** The detection methods for ncRNA. Created with BioRender.com

Non-coding RNAs are diverse and play different functions and roles from coding RNAs in the cell. Initially, there was little understanding of non-coding RNAs, which had been considered to have a limited impact on tumorigenesis and development and were called spam-free RNAs. In recent years, numerous studies have demonstrated that non-coding RNAs play important roles in the development of different types of cancers.^70^ With further research, several non-coding RNAs have been used as biomarkers for liquid biopsies in cancer^71^ (Fig. 7).

miRNAs, a small (18–23 nt) single-stranded RNA molecule involved in post-transcriptional gene regulation, belong to the subclass of non-coding RNAs. It reduces the stability of mRNAs and inhibits gene expression by binding to 3′ untranslated region recognition sites.^72^ miRNA is the most widely studied factor in cancer research and the most studied ncRNA in liquid biopsies. miR-21 and miR-155 have been found to be up-regulated in a variety of cancers and may be able to become a promising cancer liquid biopsy marker.^73^ In recent years, more and more methods have been used for miRNA detection, such as qPCR, hybridization chain reaction, rolling circle amplification, and strand displacement amplification. These methods have greatly aided the study of miRNA, particularly in understanding its two primary features: abundance and tissue stability. These properties could potentially be advantageous in the future for developing non-invasive biomarkers for patients with tumors.

Currently, the second most abundant source of ncRNAs evaluated in cancer liquid biopsies is lncRNAs. lncRNAs are non-protein-coding transcripts more than 200 nt in length, which have a wide range of biological roles.^74^ For example, they regulate the transcription of genes, influence miRNA regulation of target genes, and, through their interactions with proteins affect the function and stability of proteins. Some lncRNAs can also regulate the cell cycle, which in turn affects cell proliferation and differentiation.^75^ Studies have shown that lncRNAs may be implicated in the development of cancer in relation to their ability to regulate key cancer-associated transcriptional activators.^76^ Because of their tissue-specific expression patterns, they may contribute to tumor heterogeneity.^77^

Several known cancer-related lncRNAs are overexpressed in the serum and plasma of cancer patients, enabling them to be promising biomarker candidates for non-invasive diagnosis.^78,79^ For example, it has been found that lncRNA can mediate pancreatic ductal adenocarcinoma (PDAC), which can be used as a liquid biopsy biomarker for PDAC.^80^ Hu and his team have found that lncRNA H19 can be used as a potential biomarker for the adjuvant diagnosis of lung cancer, because of its significant elevation in the plasma of patients with lung cancer.^81^ Although a large number of lncRNAs have been identified in recent years, the specific functions of some lncRNAs and the role they play in cancer are still unknown, so we need to pay close attention to the study of lncRNAs in the future, to fully evaluate its feasibility and accuracy as a liquid biopsy for cancer. Currently, there are abundant studies on lncRNA-based diagnostic and prognostic models.^82–84^ For example, one study discovered m6A immune-associated lncRNA risk models that can accurately forecast prognosis, immunological status, and treatment response in bladder cancer.^82^ And a study utilized overlapping long non-coding RNAs (lncRNAs) to create a signature of lncRNAs linked with cuproptosis. This signature can be employed to forecast the prognosis and determine the effectiveness of immune checkpoint blockade (ICB) therapy in individuals diagnosed with hepatocellular carcinoma.^84^ Despite the lack of clinical studies on the subject, there is no doubt that the modeling of biomarkers using miRNA and lncRNA is a crucial area of development in liquid biopsy.

## CircRNA

Circular RNAs (circRNAs) are a distinct type of RNA molecules that possess a distinctive closed loop structure and do not code for proteins (Fig. 7). The initial documentation of circRNAs may be traced back to a 1971 investigation on potato spindle tuber disease. During this study, circRNAs were not yet recognized as a distinct concept, and scientists provisionally referred to them as a “virus-like” RNA with low molecular weight that has the ability to self-replicate.^85^ In 1976, Sanger et al. isolated this RNA and subjected it to different nuclease enzymes. They discovered that these RNAs were not easily broken down by most nuclease enzymes, indicating that they likely have a looped structure. This is because looped RNAs lack free ends at the 5′ and 3′ termini, making them less recognizable and degradable by nuclease enzymes. Sanger employed radioactive labeling to directly visualize the closed loop structure of virus-like RNAs. The RNA ends were labeled and it was seen that these ends were not labeled under both in vivo and in vitro circumstances, providing additional confirmation of the circRNA.^86^ The investigations conducted by Memczak et al. in 2013 and Hansen et al. in 2013 were significant contributions to the field of cyclic RNA research. These studies systematically have shown the extensive occurrence and significance of cyclic RNAs in human cells and tissues.^87^ Presently, scientists have discovered that circRNAs possess a multitude of biological roles, such as acting as miRNA sponges, controlling the splicing of precursor mRNAs, facilitating transcription, regulating their own stability and location through binding to RBPs (RNA-binding proteins), and encoding functional proteins, among others.^88^ circRNAs are not directly detectable by selective purification procedures that rely on polyA tails due to their absence of a typical polyA tail. Scientists have utilized several techniques like RT-PCR, RNAseq, northern hybridization, and high-throughput sequencing to detect circRNAs. This was achieved by developing primers that target specific reverse splice sites of circRNAs. Because of the inherent characteristics of circRNA, RNA exonuclease is unable to effectively degrade it, while linear RNA can be selectively broken down by RNA exonuclease for the purpose of enrichment.^89^ circRNAs can function as either proto-oncogenes or oncogenes in cancer, depending on the specific pathways they are connected with. One instance is circHIPK3, which can enhance the growth and movement of cancer cells by activating the miR-124/STAT3 pathway. STAT3 is a transcription factor that is linked to multiple oncogenes and the process of cell proliferation. The circHIPK3 molecule indirectly enhances the activation of the STAT3 signaling pathway by preventing the inhibitory effect of miR-124 on STAT3. This, in turn, controls the malignant activity of tumor cells.^90^ Studies have demonstrated that the circRNA ITCH functions as an oncogene in multiple types of cancer. The circ-ITCH molecule has the ability to bind to miRNAs, specifically miR-7, miR-17, and miR-214, resulting in an indirect control over the expression of its target genes. These microRNAs (miRNAs) and their target genes potentially play a role in many signaling pathways associated with tumors, including the Wnt/β-catenin system and the PI3K/AKT pathway.^91^ Aberrant expression of circ-ITCH can potentially facilitate tumor growth by disrupting the equilibrium of these pathways. It has been discovered that circ-ITCH is down-regulated as an oncogene in ovarian cancer, prostate cancer, glioma, and gastric cancer.^92^ To summarize, circRNAs contribute to the development of tumors by facilitating cell proliferation, avoiding growth inhibitors, increasing invasion and metastasis, inducing angiogenesis, disrupting cellular energy regulation, and fostering inflammation.

## Technology for the detection of liquid biopsy markers

As previously stated, liquid biopsy markers primarily include CTCs, ctDNA, exosomes, free miRNA, lncRNA, circRNA, proteins, and so on, which are detected in various ways but share some similarities. CTCs detection necessitates enrichment of CTCs, which are subsequently labeled with particular antibodies or fluorescent dyes. These markers can bind to specific antigens on the surface of circulating tumor cells, generating visible fluorescence signals under a microscope. Physical separation methods and antigen–antibody conjugation methods are the most common approaches for enriching CTCs. Traditional physical separation methods involve separating cells based on screening parameters such as cell size, density, or charge. Traditional antigen-antibody binding approaches for identifying CTCs are primarily achieved by the CellSearch system, which is based on the principle of EpCAM to trap tumor cells.^93–97^ The primary objective of ctDNA detection is to identify specific mutations. Plasma DNA is concentrated and identified by using advanced technologies such as digital PCR (dPCR) and NGS.^98–100^ The identification of exosomes involves the enrichment of exosomes and subsequent analysis of their constituents. In this context, our primary focus is on the enrichment process. The main techniques employed for this purpose include differential centrifugation, filtration, polymer precipitation, immunomagnetic beads, chromatography (specifically volumetric exclusion chromatography), and the relatively new microfluidic technology.^57,101^ The methods used for RNA detection encompass RNA-FISH, RT-PCR, Northern Blotting, RNA Sequencing, RNA Microarray, In Situ Hybridization, and various other techniques.^102–106^ Proteins can be identified using western blot and mass spectrometry techniques. The subsequent tables provide a comparison of the principles linked to each technique, as well as their respective benefits and drawbacks (Tables 1–5).

## Liquid biopsy in systemic tumors

**Fig. 8:**
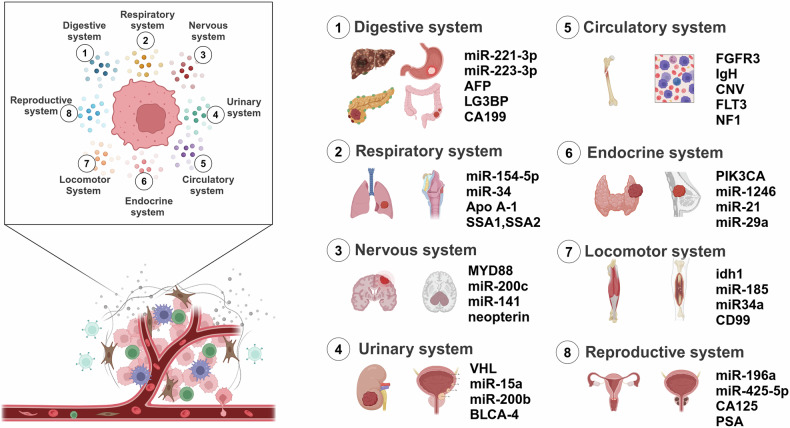
Liquid biopsy biomarkers of systemic tumors. Application of liquid biopsy in tumors of different systems and some examples of biomarkers. Created with BioRender.com

In this section we summarize the application of liquid biopsy in eight systems of tumors (Fig. 8).

## Digestive systems

The digestive system concentrates on the use of liquid biopsy in hepatocellular carcinoma (HCC), cholangiocarcinoma (CCA), CRC, pancreatic cancer (PC) and gastric cancer (GC) (Table 6).

**Table 6:** Liquid biopsy in digestive system cancers

Cancer	Liquid biomarker	Origin	Tendency	Downstream target	Function	Reference
HCC	miR-221-3p, miR-223-3p, miR-10b5p, miR-21-5p	Plasma exosome	up		Early diagnostic biomarker	^108^
	LG3BP, PIGR	Serum exosome	up		Early diagnostic biomarker	^110^
	ECE1, HOXA1, cle11a, AK055957, PFKP, EMX1 methylation	Plasma	up		Early diagnostic biomarker	^112^
	cfDNA	Plasma	up		Early diagnostic biomarker, Efficacy monitoring biomarker	^115^
	CTCs	Peripheral blood	up		Early diagnostic biomarker, Tumor recurrence biomarker	^114^
	Mixed CTCs, Mesenchymal CTCs	Peripheral blood	up		Early diagnostic biomarker, Disease progression biomarker	^116^
CCA	microRNA-21, microRNA-221	Plasma	up		Early diagnostic biomarker	^122^
	hTERT, CK19	Peripheral blood	up		Prognostic biomarker	^123^
	Osteopontin (OPN)	Serum	down	MMP1, MMP10, CXCR4	Efficacy monitoring biomarker, Prognostic biomarker	^124^
	MMP-7	Serum	up		Early diagnostic biomarker	^127^
	CYFRA 21-1	Serum	up		Early diagnostic biomarker, Disease progression biomarker	^129^
	Osteopontin (OPN)	Serum	up		Efficacy monitoring biomarker	^130^
	CTCs	Peripheral blood	up		Tumor aggressiveness biomarker, Prognostic biomarker	^131^
	cfDNA mutation	Bile	up		Prognostic biomarker	^132^
CRC	microRNA-203	Serum exosome	up	M2-TAM	Prognostic biomarker	^135^
	microRNA-21	Plasma exosome	up		Tumor recurrence biomarker, Prognostic biomarker	^137^
	miR-17-5p, miR-92a-3p	Serum exosome	up		Early diagnostic biomarker, Disease progression biomarker	^138^
	miR-25-3p	Serum exosome	up		Tumor aggressiveness biomarker	^139^
	miR-196b-5p	Serum exosome	up	STAT3	Efficacy monitoring biomarker	^147^
	miR-301a, miR-23a	Serum exosome	UP		Early diagnostic biomarker	^140^
	miR-19a	Serum exosome	up		Tumor recurrence biomarker	^142^
	QSOX1	Serum exosome	down		Early diagnostic biomarker	^145^
	ctDNA	Plasma	up		Efficacy monitoring biomarker	^151^
	ctDNA	Plasma	up		Tumor recurrence biomarker, Prognostic biomarker	^152^

## Hepatocellular carcinoma (HCC)

In the diagnosis of HCC, alpha-fetoprotein (AFP) is detected as a classical tumor marker in most patients with HCC, but low expression of AFP in some patients with HCC is detrimental to the detection of HCC by AFP. Because HCC exhibits substantial tumor heterogeneity, neither AFP nor liver biopsy currently fulfills the clinical requirements for early diagnosis or prognosis assessment.^107^ Therefore, it is necessary and meaningful to search for alternative ways of detecting HCC.

Several liquid biopsy markers can be used for early diagnosis of hepatocellular carcinoma. On the one hand, it was found to be feasible to co-detect AFP with miRNA, and the diagnostic ability of patients with low AFP expression can be improved (AUC: 0.80, specificity: 95%, accuracy: 81%) by the combined detection of AFP and miRNAs (including miR-221-3p, miR-223-3p, miR-10b5p, and miR-21-5p).^108^ On the other hand, searching for other more effective protein markers may be an effective way to improve early diagnostic ability. For example, the exosomal proteins LG3BP and PIGR can promote the transformation, invasion, and proliferation of tumor cells, which are associated with a poor prognosis, and they show greater diagnostic ability as biomarkers compared to AFP.^109,110^ As a marker released into the peripheral blood by tumors, cfDNA is usually not used for screening purposes since there is minimal necrosis of tumor cells in the early stages, and only a small amount of ctDNA is released into the bloodstream.^111^ However, a recent study has shown that the methylation properties of ctDNA have great potential in the early diagnosis of tumors. Researchers identified six optimal methylated DNA markers (MDMs), including ECE1, HOXA1, cle11a, AK055957, PFKP, and EMX1, and performed phase I and phase II clinical validation, finding them to be highly AUC (0.96), sensitive (95%) and specific (92%) in the diagnosis of HCC.^112^ Expert consensus on early screening strategies for liver cancer in China incorporates cfDNA whole genome sequencing into the whole process of early liver cancer screening.^113^ CTCs are malignant cells that undergo epithelial-mesenchymal transition (EMT) in the primary tumor. Qi et al used the CanPatrol™ CTCs enrichment technology in 112 patients with HCC, and the positive rate exceeded 90% even for early-stage disease.^114^ In addition to the early diagnosis of tumors, liquid biopsy is also beneficial for patient treatment as well as prognosis. For example, ctDNA, mentioned above, is not only involved in the early diagnosis of tumors but can also be used as an indicator of the efficacy of tumor radiotherapy. Patients with high pre-radiotherapy ctDNA expression tended to have more advanced disease and larger tumors, and after radiotherapy, patients with low ctDNA expression had significantly better prognostic tumor response, intrahepatic non-failure rate, and local control (LC) rate (*p* = 0.017, *p* = 0.035, and *p* = 0.006, respectively).^115^ In addition to the detection of the number of CTCs, the form of CTCs is also an important test. It was found that the ratio of mixed CTCs to mesenchymal CTCs can be used to discriminatie metastatic HCC patients with non-metastatic patients (AUC: 0.861).^116^ Compared to mixed CTCs, mesenchymal CTCs have a greater potential for invasion and metastasis. Bai et al. found that high expression of the CXCR4 protein was more common in mixed CTCs, which may be associated with CTCs progression and metastasis.^117^ And the Guidelines for the Diagnosis and Treatment of Primary Liver Cancer in China suggest that CTCs testing can serve as a novel clinical tool for predicting prognosis and evaluating the effectiveness of treatment for liver cancer. In conclusion, the multiple markers of liquid biopsy can compensate for the inability to detect patients with low AFP expression and play a role in treatment as well as prognosis.

## Cholangiocarcinoma (CCA)

The tumor’s stealthy growth seriously jeopardizes their early discovery, preventing patients from accessing potentially curative treatments.^118^ Additionally, the patient’s fragile and advanced illness state increases the danger of bleeding and peritoneal seeding, and the tiny amount of tissue retrieved might not be sufficient for confirmation by cytology or histology.^119^ For these reasons, liquid biopsy is essential for both the prognosis and diagnosis of cholangiocarcinoma.

The main markers that have been studied in cholangiocarcinoma (CCA) include cfDNA, CTCs, and miRNA. Compared with healthy control specimens, miR-21 and miR-221 showed significant overexpression in the plasma of patients, and higher circulating miR-21 expression was associated with poorer prognosis in ICCA.^120^ However, the current study found that high expression of miR-21 and miR-221 was not only detected in CCA but also in HCC and other liver diseases.^121,122^ Therefore, it is possible that the combination of miR-21 and miR-221 with other markers may be useful for the detection of CCA. For example, high levels of cytokeratin-19 (CYFRA 21-1), MMP-7, osteoblasts, periostin, and IL-6 can be detected in the serum of patients with CCA, which may be helpful for further diagnosis of CCA.^123–130^ In addition to miRNAs, CTCs is an important marker in liquid biopsy of CCA. High expression of CTCs is associated with strong tumor aggressiveness and short survival, and thus evaluation of CTCs may help identify CCA patients at risk of early death.^131^ Unlike miRNAs and CTCs, which are detected in blood, cfDNA can be detected in the bile of CCA patients, and tumor recurrence and prognosis can be inferred mainly by detecting single-nucleotide variants, insertions, and deletions of cfDNAs, but not their expression.^132,133^

## Colorectal cancer (CRC)

Colorectal cancer is a complex illness characterized by numerous genetic or somatic changes, and it is identified in less than half of cases when it is locally advanced.^134^ Thus, the implementation of liquid biopsies is necessary to enhance the accuracy of colorectal cancer diagnosis and to forecast the advancement of the disease.

miRNAs have a crucial role in various aspects like tumorigenesis, proliferation, metastasis, and drug resistance in CRC. For example, high expression of miR-193a and miR25-3p, miR-17-5p and miR-92a-3p, miR-21, and miR-203 promotes liver metastasis by inducing vascular permeability/angiogenesis.^135–139^ Therefore, miRNAs have the potential to serve as an effective liquid biopsy marker. Several scholars have studied miRNAs and found that a variety of miRNAs, such as miR-23a, miR-301a,^140^ as well as miR-17-92a and miR-19a^141,142^ are significantly overexpressed in the blood of tumor-bearing patients and are predictive of early tumorigenesis as well as tumor aggressiveness. Consequently, some miRNAs can distinguish CRC patients from the population and help in the early diagnosis of CRC. As for CTCs, patients with colorectal cancer had higher CTCs counts than those with colorectal polyps (*P* < 0.001).^143^ And CTCs counts were positively correlated with CRC disease stage, with sensitivities ranging from 89 to 97% across the range of disease severity.^144^ However, not all liquid biopsy markers are present in the form of high expression in patients’ blood. The exosomal cargo protein QSOX1 is significantly reduced in the blood of tumor patients compared with healthy human controls while Glypican-1 (GPC1) is significantly increased in exosomes, and a series of recent studies have suggested that dysregulation of exosomal proteins could serve as a promising novel biomarker for the early diagnosis and non-invasive risk stratification of CRC.^145^ At present the monitoring of single extracellular vesicles (SEV) is also helpful in the diagnosis of colorectal cancer. A study has developed a new sensor that combines a DNA aptamer capable of explicitly binding to SEV surface proteins with a single microbead capable of immunoadsorbing EVs, allowing for the direct and rapid monitoring of SEV. Clinical trials have shown that it is able to detect exosomes directly from 2 μL plasma samples, and indicated that cancer patients have higher levels of CD63, EpCAM double-positive exosomes than healthy controls.^146^

In addition to the early diagnosis of tumors, the observation of the efficacy of tumor therapy and the prognosis of survival are important purposes of liquid biopsy. Up-regulation of miR-196b-5p in patients with CRC promotes chemoresistance to 5-FU.^147^ Besides, high expression of CTCs in patients’ blood is often a marker of high tumor recurrence rate and poor prognosis. The results of a study that performed CTCs counts on treatment days 1 and 15 showed that patients with high CTCs counts at baseline had worse overall survival (*p* < 0.001).^148^ In addition, the detection of CTCs surface markers such as thymidylate synthase and excision repair protein RAD23 homolog B can help to predict chemo-/radiotherapy resistance in patients.^149^ According to the Chinese Expert Consensus on Clinical Detection of Molecular Markers for Colorectal Cancer, CTCs could be effective for early screening, prognosis, and efficacy assessment of the disease.^150^ CtDNA has been shown to be useful in detecting the efficacy of surgery and chemotherapy and to play a role in the prediction of tumor recurrence. In patients receiving chemotherapy, downregulation of ctDNA is a predictor of response to treatment.^151^ Conversely, upregulation of ctDNA after surgery predicts a higher five-year risk of recurrence and poorer overall survival.^152^ Also, it is encouraging to note that studies have found a high degree of concordance between ctDNA mutations detected in the bloodstream and those found in biopsies of tumor tissues,^153^ suggesting that liquid biopsies may be able to play an even greater role in the future.

## Pancreatic cancer (PC)

Pancreatic ductal adenocarcinoma (PDAC) is the most common form of PC and accounts for more than 90% of PC^154^. The biology of PDAC is highly diverse and intricate, and its diversity is seen as a primary factor contributing to its resistance to therapies. Tumor heterogeneity is present not only across different patients (inter-tumor heterogeneity), but also within the same tumor (intratumor heterogeneity). Additionally, there is temporal heterogeneity caused by changes in PDAC over time and during treatment.^155^ Consequently, the early detection and monitoring of tumor development in PDAC via tissue biopsy is difficult. As a result, liquid biopsy holds significant research value in the diagnosis of PDAC and other related areas.

In the early diagnosis of PC, the number of CTCs can be effectively distinguished between PC patients and healthy controls, which has a high specificity (96.4%) but insufficient sensitivity (75.0%).^156^ Expert consensus of Oncology Committee of Chinese Medical Association in early diagnosis and treatment of pancreatic cancer states that CTCs can be used as a marker for early diagnosis and differential diagnosis of pancreatic cancer.^157^ Compared to CTCs, circulating epithelial cells (CECs) had a better performance in early diagnosis, with 77.8% patients showing detectable CECs, while only 15.8% of controls had detectable CECs.^158^ In early diagnosis, ctDNA relies heavily on the detection of its mutations. Since KRAS mutations are the most common genetic alterations in pancreatic cancer, and are present in more than 90% of patients, several scholars have investigated the use of KRAS mutations in liquid biopsy. It was found that detecting KRAS mutations by ctDNA alone had poor sensitivity (35.2%), accuracy (51.0%), and AUC (0.683).^159^ This may be due to the coexistence of KRAS mutations in a variety of other tumors.^160^ Therefore, the diagnostic power of ctDNA mutations can be effectively enhanced by combining ctDNA mutations with other markers, e.g., ctDNA mutations in combination with proteins,^161^ ctDNA mutations in combination with CA19-9, etc.^29^. Of these, the combination with CA199 had significantly higher sensitivity (78%) and specificity (91%).^29^ Compared to ctDNA mutations, methylation of ctDNA showed a stronger potential in early diagnosis, and methylation of ADAMTS1 and BNC1 performed well in the early diagnosis of PDAC in terms of its sensitivity (97.4%), specificity (91.6%), and AUC (0.95).^162^ Although CA19-9 is a classical tumor marker, it lacks specificity in early diagnosis as CA19-9 lacks tumor specificity. Therefore, monitoring CA19-9 in combination with other markers can help to improve the specificity of PC diagnosis. One study found that 66.10% of miRNA had better diagnostic value compared to CA19-9 by analyzing a variety of miRNAs.^163^ Expert consensus on the molecular diagnosis of early-stage pancreatic cancer (2023 edition) recommends miRNA combinations as markers for early-stage precision diagnosis of pancreatic cancer to provide guidance to clinicians. Moreover, miRNAs in combination with CA19-9 may have better application value.^164^ When combined with CA19-9, the AUC can be significantly increased compared to CA199 alone.^165^ In extracellular vesicles, the difference in extracellular vesicle long RNA levels had a very high AUC (0.949) in early diagnosis43.^166^ According to *CACA TECHNICAL GUIDELINES FOR HOLISTIC INTEGRATIVE MANAGEMENT OF CANCER*, the combination of CTCs, ctDNA, exosomes, microRNAs, etc., with CA19-9 can improve the accuracy of PC diagnosis. However, its widespread use in the clinic needs to be supported by high-quality clinical research.

For chemoresistance in PC, a variety of liquid biopsy markers can be useful. Although CTCs counts may not be effective in predicting chemotherapy efficacy,^167,168^ detection of CTCs molecular features can help predict therapeutic efficacy, such as CXC-motif chemokine receptor 4 (CXCR4).^169,170^ Compared to CTCs, ctDNA has been more extensively studied in the detection of chemotherapy treatment. On the one hand, the probability of detectable ctDNA in the blood of patients receiving neoadjuvant chemotherapy is dramatically reduced.^171^ On the other hand, a decrease in cfDNA mutant allele fraction (MAF) predicts a response to chemotherapy, and drug-resistant patients show an increase in ctDNA MAF during the course of disease progression.^172^ Various ncRNAs such as miR-20a-5p and miR-373-3p have been found to be associated with chemotherapy resistance^173,174^ and have potential as indicators to monitor therapeutic efficacy. However, current studies on ncRNAs and EVs in chemoresistance have focused on mechanistic studies^175^ and more clinical studies are needed for validation.

In the prognostic prediction of PC, the positivity of CTCs was associated with poor prognosis in patients with PDAC.^176,177^ The KRAS mutation in ctDNA was found to be significantly associated with the prognosis of the patients.^178^ Mutated patients have a tendency to relapse early and have a significantly lower overall survival, and recurrence-free survival, as compared to unmutated patients.^179^ Multiple miRNAs were combined in one study, and the score model constructed could be used to predict 5-year OS in patients, which was lower in patients with higher risk scores.^180^ Similarly, the combined diagnosis of multiple markers in EVs (EV-CK18 mRNA, EV-CD63 mRNA, EV-miR-409, cfDNA concentration, and CA19-9) in the monitoring of PDAC metastasis has a favorable efficacy (accuracy of 84%, sensitivity of 78%, specificity of 88%, AUC of 0.85) due to conventional imaging.^181^

Although CA19-9 is a commonly used tumor marker, there are still 10% of patients who do not synthesize CA199, which is detrimental to the diagnosis of PC. Since the synthesis of CA19-9 is affected by common variants in the fucosyltransferase (FUT) enzymes FUT3 and FUT2, the combination of CA199 with FUT significantly improved the AUC (0.84-0.92).^182^ Measurement of the associated glycan DUPAN-2 is useful in individuals unable to synthesize CA19-9. A recent study found that the accuracy of early pancreatic cancer blood tests (CA19-9 and DUPAN-2) was improved when monitored by measuring the FUT2/FUT3 genotype subgroups and combining CA199 with DUPAN-2.^183^ Therefore, the detection of FUT added to patients with low CA19-9 expression may contribute to a more effective diagnosis of pancreatic cancer.

## Gastric cancer (GC)

The primary indications of gastric cancer are nonspecific and typically involve dyspepsia, which is indicative of peptic ulcers. Patients and doctors sometimes overlook these symptoms, and a physical examination reveals no evident anomaly, or solely the presence of blood in the stool.^184^ Hence, it is imperative to discover novel and more efficient approaches for early detection of stomach cancer.

In early diagnosis, CTCs were found in 90.5% of patients. The sensitivity and specificity rates for detecting CTCs were 85.3% and 90.3%, respectively, among patients with gastric cancer and healthy individuals. Furthermore, it exhibits enhanced sensitivity in detecting advanced gastric cancer patients.^185^ Research has shown that the amount of cfDNA in the plasma of patients with stomach cancer is higher compared to healthy individuals.^186^ When comparing CTCs to cfDNA, it is found that cfDNA has a greater sensitivity (96.67%) and specificity (94.11%) in the early detection of gastric cancer. Additionally, it has an AUC value of 0.9914.^187^ In recent times, various methods have been developed to identify methylation in cfDNA for the purpose of early detection. These techniques offer a high level of accuracy (>90%) in terms of specificity, however their sensitivity is comparatively lower.^188,189^ Hence, there remains ample opportunity for enhancement. Certain circular RNAs (cirRNAs) have the potential to be utilized for early diagnosis.^190^ By combining various cirRNAs to create a prediction model, it is possible to more accurately distinguish between patients and healthy individuals.^191^ Moreover, the use of many miRNAs can be employed for the prompt detection of gastric cancer, exhibiting an impressive area under the curve (AUC) value of 0.9299.^192^ Furthermore, it was discovered that the levels of serum exosomal protein TRIM3 were notably decreased in patients with gastric cancer compared to individuals without the disease.^193^

Liquid biopsy can also reveal cancer progression. Several studies have indicated that CTCs are linked with GC stage, and the amount of CTCs is higher in patients with high stage than in individuals with low stage.^194,195^ CTCs was discovered in 96% of metastatic gastric cancer patients,^196^ and the number of CTCs was considerably higher in patients with GC distant organ metastases than in healthy controls and non-metastatic patients.^197^ The plasma cfDNA was demonstrated to show an elevated trend in its concentration with the progression of gastric cancer.^198^ And the serum cfDNA expression level of patients with stages III-IV was significantly higher than that of patients with stageI.^199^ The role of miRNAs in gastric cancer development has been identified, for example, down-regulation of either miR-17-5p or miR-4742-5p significantly inhibits GC cell proliferation, invasion, and metastasis,^200,201^ and HULC promotes ubiquitous cell invasion and migration through the Wnt/βcatenin signaling pathway,^202^ However, there is currently more mechanistic research and a lack of clinical data to validate the results. Upregulation of exosome hsa_circ_0015286 was found to be closely associated with tumor size, clinical stage, and lymph node metastasis, with an AUC of 0.778, a sensitivity of 82.1%, and a specificity of 65.7% in gastric cancer.^203^

During GC treatment, both CTCs and cfDNA have been found to be useful in predicting efficacy during ICB treatment. Immune checkpoint blockade therapy efficacy can be predicted by analyzing the number and type of CTCs and CTCs-PD-L1 expression.^204^ CfDNA, on the other hand, can be used to predict therapeutic efficacy by detecting microsatellite instability (MSI) in GC,^205^ For chemotherapy, ncRNAs have been mentioned more often, on the one hand, multiple miRNAs (miR100, miR-34a, miR-23a, miR-30a, let- 7g, miR-342, miR-16, miR-181, miR-1, and miR-34) were found to correlate with chemo-sensitivity through data prediction,^206^ and on the other hand, some ncRNAs were confirmed to be associated with chemo-sensitivity through basic research. For example, miR-30a with cisplatin chemotherapy,^207^ hsacirc_004413, miR-145-5p, circCPM with 5-FU resistance.^208,209^ Therefore, ncRNA may be useful for chemotherapy efficacy prediction, which needs to be supported by more clinical data. After undergoing surgical treatment, the expression level of serum exosomal LncRNAH19 was significantly reduced compared with the preoperative level, and its AUC for diagnosing GC was up to 0.849, with a sensitivity and specificity of 74.36% and 83.95%, respectively, and its expression level was significantly correlated with the TNM stage.^210^

For patient prognosis, the OS as well as PTS of patients after treatment showed a significant negative correlation with CTCs and ctDNA,^211–213^ and the detection of cfDNA levels was helpful in predicting the recurrence of patients.^214^ Methylation levels of the cfDNA genes such as RASSF1A, SOX17, and wi −1 were significantly correlated with reduced PFS as well as OS.^215^

## Respiratory system

For the application of liquid biopsy in the respiratory system, we focus on lung cancer, laryngeal squamous cell carcinoma (LSCC), and nasopharyngeal cancer (Table 7).

**Table 7:** Liquid biopsy in respiratory system cancers

Cancer	Liquid biomarker	Origin	Tendency	Downstream target	Function	Reference
LC	cfDNA methylation	Plasma	up		Early diagnostic biomarker	^217^
	CDO1, HOXA9, AJAP1, PTGDR, UNCX, MARCH11 *methylation*	Serum, Pleural effusion, Ascites	up		Early diagnostic biomarker, Prognostic biomarker	^218^
	RASSF1A, CDKN2A, DLEC1 methylation	Plasma	up		Early diagnostic biomarker	^219^
	ctDNA	Plasma	up		Efficacy monitoring biomarker	^222^
	CTCs	Peripheral blood	up		Early diagnostic biomarker	^225^
	let-7i-3p, miR-154-5p	Serum	down		Early diagnostic biomarker	^226^
	miRNA	Plasma exosome	up		Early diagnostic biomarker	^228^
	SSA1,SSA2	Serum, Plasma	up	MMP-9	Early diagnostic biomarker, Tumor aggressiveness biomarker	^221^
LSCC	ctDNA	Plasma, Saliva	up		Early diagnostic biomarker	^240^
	ctDNA methylation	Plasma	up		Early diagnostic biomarker, Disease progression biomarker, Prognostic biomarker, Disease progression biomarker	^242^
	CTCs	Peripheral blood	up		Early diagnostic biomarker	^237^
	CTCs	Peripheral blood	up		Prognostic biomarker, Efficacy monitoring biomarker	^238^
	CTCs	Peripheral blood	up		Prognostic biomarker	^239^
	miR-21	Serum exosome	up		Early diagnostic biomarker	^245^
	miR-155	Plasma	up		Early diagnostic biomarker	^246^
	miRNA-130a	Plasma	down		Disease progression biomarker	^247^
	miR-632	Serum	up		Early diagnostic biomarker, Prognostic biomarker	^248^
	Microbiota	Mouthwash			Early diagnostic biomarker	^236^
NPC	EBV DNA	Plasma	up		Early diagnostic biomarker, Tumor recurrence biomarker	^251^
	EBV DNA	Plasma	up		Efficacy monitoring biomarker	^253^
	EBV DNA methylation	Saliva	up		Early diagnostic biomarker	^254^
	EBV microRNA	Serum	up		Early diagnostic biomarker	^258^

## Lung cancer

The high mortality rate of lung cancer is mainly due to the late detection and diagnosis of lung cancer and the fact that most lung cancer patients show signs of metastasis at the time of symptom onset, leading to a decrease in the overall survival rate of lung cancer.^3^ Therefore, early diagnosis and early treatment are effective measures to reduce the mortality rate of primary lung cancer patients. In screening for lung cancer, ctDNA plays a role as a class of liquid biopsy markers in the diagnosis, treatment, and prognosis of the disease. Firstly, not only the expression of ctDNA is upregulated in lung cancer patients, but also its methylation level is upregulated in early-stage lung cancer, so ctDNA may be used as an effective marker for screening early-stage tumors.^216–219^ The exosome, which is currently popular in liquid biopsies, has likewise been found to serve as a liquid biopsy biomarker for lung cancer. In particular, exosomal proteins, a variety of proteins like SAA1, SAA2, Apo A-1, etc., have been found to be abnormally expressed in lung cancer patients and are considered to be potential markers for the early detection of lung cancer.^220,221^ Although CTCs do not play a significant role in early cancer screening, the number of CTCs detected does correlate strongly with tumor efficacy and prognosis.^222–225^ This idea was well confirmed in a recent study, in which patients with high CTCs counts before or after treatment had a significantly worse prognosis than those with low CTCs.^224^ The CSCO Small Cell Lung Cancer Diagnostic and Treatment Guidelines state that tracking CTCs can assist in accurately determining the disease’s clinical stage, which will help in selecting the best course of action, directing each patient’s unique course of care, keeping an eye on the tumor’s metastasis and recurrence, assessing the effectiveness of the treatment, and forecasting the prognosis for survival. miRNAs, as a prognostic biomarker for lung cancer, have also become an important component of liquid biopsies for lung cancer.^226^ In addition, miRNAs have been found to be involved in a variety of pathogenetic processes in cancer, such as proliferation, migration, and drug resistance.^227,228^ Therefore, miRNAs have the potential to become an effective biomarker for understanding tumor progression as well as treatment efficacy. In addition to this, the amount of ctDNA also reflects the different stages of lung cancer, and the detection rate of ctDNA rises with tumor stage, with ctDNA detected in 100% of plasma specimens from patients with stage II-IV NSCLC.^229^ Moreover, the expression of ctDNA is highly correlated with the volume and size of the tumors, and thus ctDNA detection may be synergistic with imaging, and more helpful in understanding the course of the patient’s disease. The 2021 IASLC NSCLC Liquid Biopsy Consensus states that plasma ctDNA can be considered a useful tool for genotyping newly diagnosed patients with advanced NSCLC, and that the results are often complementary to those from tissue analysis.^230^ Also, ctDNA mutations have been found to be of some significance in lung cancer, but their mutations are not associated with early screening of tumors but rather tend to guide the selection of treatment regimens. Since it has been found that drug-resistant recurrence in many patients is associated with mutations in ctDNA, ctDNA testing may be used as an adjunctive means of detecting therapeutic efficacy and providing more rational clinical drug use.^222,231^

## Laryngeal squamous cell carcinoma (LSCC)

Laryngeal squamous cell carcinoma (LSCC) is the second most common cancer of the respiratory system after lung cancer.^232^ Due to the lack of early disease indicators, the diagnosis is typically made at a late stage. 40% of patients are diagnosed with lymph node metastases and have a bad outcome.^233^ Currently, imaging and tissue biopsy are the predominant diagnostic techniques of head and neck squamous cell carcinoma (HNSCC). However, imaging tools make it difficult to detect micrometastases and persistent lesions in the early stages. Because different metastatic lesions might arise in diverse tumor genetic landscapes, a single tissue sample cannot adequately capture tumor heterogeneity.^234^ As a result, clinical detection strategies to improve early identification and prolong survival of HNSCC are critical.

Classical CTCs as well as ctDNA have been shown to be associated with LSCC. Current studies have shown that ctDNA can be detected in the plasma and saliva of patients with early and advanced disease and that the amount of ctDNA is higher in patients with advanced and metastatic cancers than in patients with early-stage disease.^235^ A recent study found that ecological dysregulation of the oral microbiome is a key hallmark of LSCC and that LSCC can be identified by detecting microbiota in mouthwash, which provides a novel model for liquid biopsy of LSCC.^236^

And a series of studies have found that liquid biopsies have great potential for predicting the treatment efficacy and prognosis of patients. For CTCs, in addition to its early diagnostic role, it can also be used for treatment efficacy testing. CTCs counts are significantly reduced in tumor patients after treatment, and CTCs-negative patients have improved survival compared to CTCs-positive patients.^237,238^ Patients with high preoperative CTCs expression have a worse postoperative prognosis, and reduced CTCs values have been associated with an improved response to treatment.^239^. CtDNA may be associated with tumor recurrence and can appear prior to recurrence, which plays a predictive role.^240^ In addition, hypermethylation of ctDNA has been shown to correlate with tumor stage,^241^ and patients who exhibit high methylation levels early in life have a higher risk of death.^242^ Many miRNAs have been found to be dysregulated in cancers such as LSCC and are associated with tumor progression, and therefore miRNAs have received more attention in liquid biopsies for LSCC.^243,244^ To date, several miRNAs have been found to be highly expressed in the plasma of LSCC patients,^245,246^ and are strongly correlated with tumor size, advanced stage, and LNM.^246^ In addition, the expression of miRNAs such as miR130a and miR-632 has been associated with OS and DFS.^247,248^ LncRNA expression has been significantly correlated with the occurrence of LNM, advanced T-classification, and clinical stage, and may serve as a useful indicator of laryngeal cancer development.^245^

## Nasopharyngeal cancer

Nasopharyngeal cancer is a malignant tumor of the respiratory system, which is often associated with EBV infection, and its symptoms are nonspecific and difficult to detect at an early stage.^249^ Because of the high correlation between nasopharyngeal cancer and EBV infection, EBV detection plays a very important role in liquid biopsy of nasopharyngeal cancer, and the circulating free EBV DNA tends to have the greatest role in early detection of nasopharyngeal cancer.^250^ By detecting the copy number of circulating free EBV (cfEBV) DNA, not only can it reflect the tumor load of patients, but also can be used for the prognosis prediction of metastatic nasopharyngeal cancer.^251–253^ Moreover, it has been found that the methylation of EBV DNA is significantly increased in the saliva of nasopharyngeal cancer patients, which suggests that it may be relevant to the detection of nasopharyngeal cancer.^254^ In addition, the detection of cfEBV DNA has shown other detection values, some scholars have found that the use of cfEBV DNA to guide routine imaging can effectively improve the detection efficiency and reduce the cost of detection.^255^ There is still much room for exploration of EBV in liquid biopsy of nasopharyngeal carcinoma. EBV-associated proteins such as EBNA1, EBER1, EBER2, etc. have been found to be useful in the diagnosis of nasopharyngeal cancer.^256,257^ Besides EBV-associated assays, various exosomal miRNAs have been found to be increased in the blood of patients with nasopharyngeal cancer, and anti-miRNA oligonucleotides (antagomiR) have a greater potential to become a therapeutic approach for nasopharyngeal cancer.^258,259^

## Nervous system

In this part, we mainly introduce the application of liquid biopsy in gliomas as well as central nervous system lymphomas (Table 8).

**Table 8:** Liquid biopsy in neverous system cancers

Cancer	Liquid biomarker	Origin	Tendency	Downstream target	Function	Reference
Glioblastoma	CTCs	Peripheral blood	up		Early diagnostic biomarker, Efficacy monitoring biomarker	^269^
	ctDNA mutation	CSF	up		Early diagnostic biomarker	^260^
	ctDNA H3K27M mutation	CSF	up		Early diagnostic biomarker, Efficacy monitoring biomarker	^272^
	ctDNA methylation	CSF	up		Early diagnostic biomarker	^268^
	MCPH1 methylation	Serum	up		Early diagnostic biomarker, Efficacy monitoring biomarker	^274^
	miR-320, miR-574-3p	Serum exosome	up		Early diagnostic biomarker	^276^
	miRNA	CSF	up		Early diagnostic biomarker	^277^
PCNSL	MYD88, CARD11, CD79 mutation	CSF	up		Early diagnostic biomarker	^280^
	MYD88	CSF	up		Early diagnostic biomarker	^284^
	miR-200c, miR-141	CSF exosome	down	ATP1B3, DYNC1H1, MATR3, NUCKS1, ZNF638, NUDT4, RCN2, GNPDA1, ZBTB38, DOLK	Early diagnostic biomarker, Efficacy monitoring biomarker	^289^
	SPP1, MARCKS, NPM1, VIM	CSF exosome	up		Early diagnostic biomarker	^291^
	IL-10, sIL-2R	CSF	UP		Early diagnostic biomarker	^292^
	IL-10	CSF	up		Early diagnostic biomarker	^293^
	Neopterin	CSF	UP		Early diagnostic biomarker	^295^

## Gliomas

Gliomas are the most prevalent primary malignant brain tumors in adults. Glioblastomas are highly malignant, with an average survival of 14.6 months.^260,261^ Early diagnosis of gliomas and therapeutic testing are therefore important for patients. The principal tool for monitoring gliomas is conventional magnetic resonance imaging, which has problems in separating true progression (TP) from pseudoprogression.^262^ As a result, more reliable and sensitive approaches are required to assess tumor response and evolution. Currently, liquid biopsy of gliomas involves specimens from blood and cerebrospinal fluid.

Firstly, for the early diagnosis of tumors, as EpCAM is widely expressed on the surface of CTCs derived from cancer cells, most CTCs detect cells targeting EpCAM, but EpCAM is not present in GBM cells.^263^ Thus, it has been suggested that circulating brain tumor cells are detected by GBM-specific expression of CD14, CD16, etc.^264^ In comparison to blood, CTCs in the cerebrospinal fluid are more readily identifiable and distinguishable from other cells,^265^ which may result from the presence of the blood-brain barrier and the more complex cellular composition of blood. According to NCCN Clinical Practice Guidelines in Oncology, Version 3.2020 on Central Nervous System Cancers, CTCs improve tumor cell detection and efficacy evaluation sensitivity.^266^ Secondly, the detection of ctDNA is also of diagnostic significance for gliomas, and studies conducted by several scholars have demonstrated that the sensitivity and accuracy of tumor ctDNA detection in cerebrospinal fluid is better than that in plasma compared with blood.^261,267^ In addition to mutations of ctDNA, its methylation can be used in the detection of cerebrospinal fluid, and the detection of ctDNA methylation can help analyze the subtypes of gliomas.^268^ Also, liquid biopsy can monitor the tumor progression. First is the classical CTCs, Various studies have illustrated that the number of CTCs does not only correlate with tumor progression, as well as prognosis.^264,269^ CTCs identification techniques may be taken into consideration for the evaluation of meningeal metastases, according to the Chinese Guidelines for Integrated Diagnosis and Treatment of Tumors—Metastatic Tumors of the Central Nervous System. In the detection of ctDNA, the detection of target mutations has received more attention. The detection of mutations can predict the degree of tumor malignancy.^260^ Liquid biopsy can be also predictive for the treatment and prognosis of gliomas. CTCs may correlate with tumor resistance.^270^ And the detection of ctDNA mutations can monitor the response to drug therapy.^260,271^ CtDNA mutations can also be used to select appropriate targeted therapeutic drugs, which is more conducive to the rational use of medication to improve the efficacy of treatment.^272^ Detection of ctDNA methylation in serum has revealed that the serum markers can reflect the characteristics of tissues and can effectively differentiate between gliomas and other malignant tumors, which can help in the diagnosis of gliomas as well as in the prediction of their prognosis.^273,274^ In addition to ctDNA in circulating tumor nucleic acids, miRNA is also a point of detection. Although miRNAs have advantages such as easy identification, their faster degradation leads to hindrance in the detection process. However, when miRNAs are incorporated into extracellular vesicles like exosomes, their degradation process is impeded, making them more stable and easier to detect.^275^ Detection of miRNAs in exosomes therefore currently appears to be positive in various aspects of the diagnosis of gliomas, for example, RNA RNU6-1 has been recognized as an identifying biomarker for GBM.^276^ Cerebrospinal fluid has been shown to be a source of GBM-specific 9 miRNAs.^277^ In addition, the detection of exosomal proteins has also proved to be promising for research.^275^ 90% of GBM patients have at least one protein differently expressed in their exosomes, including EGFR, EGFRvIII, podoplanin, and IDH1.^278^ Furthermore, chloride intracellular channel 1 identified in exosomes enhances GBM growth and invasiveness, and is associated with poor prognosis.^279^ Currently, detecting changes in protein levels in body fluids or tissues is the most commonly used diagnostic method for the diagnosis, treatment, and prognosis of gliomas.

## Primary central nervous system lymphoma (PCNSL)

Unlike other lymphomas, primary CNS lymphoma is not easily recognized and responded to by immune cells due to the blood-brain barrier and is therefore considered an “immune-privileged (IP)” lymphoma.^280^ Thus, timely diagnosis and treatment are crucial for improving patient prognosis and survival. Due to the difficulty of sampling tissue biopsies, liquid biopsies have the potential to be used in conjunction with radiologic features in the diagnosis of PCNSL.^281^ Currently, ctDNA is the most frequently discussed liquid biopsy for CNS lymphomas, but several studies have failed to find a relationship between the number of ctDNAs and the diagnosis of lymphomas, etc. More attention has been paid to ctDNA mutations such as MYD88, CARD11, CD79B, etc.^280,282,283^. Among them, MYD88 is the most well-researched, and it has been classified as a diagnostic marker for PCNSL.^280^

Several studies have demonstrated that detection of the MYD88 mutation in cerebrospinal fluid or plasma not only allows for the early diagnosis of PCNSL but also helps in the prediction of efficacy and drug resistance of chemotherapy and other therapeutic measures.^284^ Currently, the technology of ctDNA detection is constantly being updated, and a new rapid genotyping system (GeneSoC) based on microfluidic thermocycling technology with RT-PCR has recently made it possible to greatly reduce the detection time compared with the previous NGS and droplet digital PCR,^285,286^ which is more conducive to intraoperative detection and monitoring of the therapeutic efficacy.^284,287^ Liquid biopsy of ctDNA can reduce the impact of spatial heterogeneity of the tumor compared with tissue biopsy, and a recent study found that liquid biopsy detects ctDNA mutations earlier than tissue biopsy, so liquid biopsy of ctDNA has great potential for clinical application in PCNSL.^288^

In addition to the most attention in ctDNA, miRNAs have also been found to be useful as monitoring markers for PCNSL.^289^ There is a lack of research on miRNA compared to ctDNA, and miRNAs are currently mainly detected in exosomes due to the greater stability of miRNAs.^290^ The expression levels of miRNAs such as miR-200c and miR-141 etc. can be used to diagnose PCNNSL as well as to monitor the efficacy of chemotherapy.^289^ In addition to miRNAs, a variety of phosphoproteins associated with PCNNSL in cellular vesicles, including SPP1, MARCKS, NPM1, and VIM, have the potential to be used as markers of PCNNSL.^291^ Some inflammatory factors, such as CSF neopterin, the interleukin (IL)-10, CXCL13, etc. have been found to be up-regulated in the cerebrospinal fluid of PCNSL patients.^280,292–294^ Moreover, CSF neopterin has been found to be significantly higher in PCNSL patients than in patients with other brain tumors and pseudo-inflammatory encephalopathies, and thus neopterin levels may help to differentiate PCNSL from other CNS tumors.^295^

## Urinary system

The liquid biopsy in urology has been focused on the following four tumors, including renal cell carcinoma (RCC), bladder cancer (BLCA), Wilms’ tumor (WT), and uroepithelial carcinoma (Table 9).

**Table 9:** Liquid biopsy in urologic system cancers

Cancer	Liquid biomarker	Origin	Tendency	Downstream target	Function	Reference
RCC	ctDNA	Plasma	up		Prognostic biomarker, Disease progression biomarker	^308^
	cfDNA	Plasma	up		Early diagnostic biomarker, Prognostic biomarker	^309^
	TP53 mutation	Plasma	up		Prognostic biomarker	^310^
	VHL, BAP1, PBRM1 mutation	Plasma	up		Prognostic biomarker	^311^
	ctDNA methylation	Urine, Plasma	up		Early diagnostic biomarker	^299^
	miR-21-5p, miR-150-5p, miR-145-5p, miR-146a-5p	Serum	up		Early diagnostic biomarker	^300^
	miR-328-3p	Urine	down		Prognostic biomarker	^301^
	miR-122-5p, miR-206	Serum	up		Prognostic biomarker	^302^
	miR-15a	Urine	up		Early diagnostic biomarker, Prognostic biomarker	^313^
	MiR-30a-5p methylation	Urine	up		Early diagnostic biomarker, Prognostic biomarker	^303^
	miR-210, miR-1233	Serum exosome	up		Early diagnostic biomarker	^315^
	has-mir-149-3p, has-mir-424-3p	Plasma exosome	up		Early diagnostic biomarker	^316^
	has-mir-92a-1-5p	Plasma exosome	down		Early diagnostic biomarker	^316^
BLCA	CTCs	Peripheral blood	up		Early diagnostic biomarker	^340^
	CTCs	Peripheral blood	up		Efficacy monitoring biomarker	^345^
	CTCs	Peripheral blood	up		Prognostic biomarker	^341^
	p16(INK4a) methylation	Serum	up		Early diagnostic biomarker	^324^
	APC, GSTP1, TIG1 methylation	Serum	up		Prognostic biomarker	^325^
	p14ARF methylation	Plasma	up		Tumor recurrence biomarker	^326^
	CDH13 methylation	Serum	up		Prognostic biomarker	^327^
	ctDNA VAF	Plasma	up		Efficacy monitoring biomarker	^348^
	ctDNA VAF	Plasma	up		Disease progression biomarker	^349^
	ctDNA	Plasma	up		Efficacy monitoring biomarker, Tumor recurrence biomarker	^350^
	miR-19a	Plasma	up	PTEN	Early diagnostic biomarker	^328^
	miR-200b	Plasma	up		Early diagnostic biomarker	^329^
	miR-92, miR-33	Plasma	down		Early diagnostic biomarker	^329^
	miR-663b	Plasma exosome	up	Ets2 deterrence factor	Early diagnostic biomarker	^333^
	BLCA-4	Urine	up		Early diagnostic biomarker	^334^
	MCM5	Urine	up		Early diagnostic biomarker	^335^
WT	miR-124-3p/miR-9-3p/miR-218-5p/miR-490-5p/miR-1538	Serum	up		Early diagnostic biomarker	^549^
	TP53 mutation	Plasma, Serum, Urine	up		Tumor recurrence biomarker	^358^
	ctDNA	Serum	up		Early diagnostic biomarker, Prognostic biomarker	^359^
	Hyaluronidase	Urine	up		Early diagnostic biomarker	^361^
	Basic fibroblast growth factor (bFGF)	Urine	up		Early diagnostic biomarker, Prognostic biomarker	^362^
UC	cfDNA	Plasma	up		Early diagnostic biomarker	^364^
	ctDNA mutation	Plasma	up		Efficacy monitoring biomarker	^365^
	ctDNA methylation	Urine	up		Early diagnostic biomarker	^367^
	*TERT mutation, ONECUT2 methylation*	Urine	up		Early diagnostic biomarker	^371^
	miR-1343-5p, miR-6087	Serum	up		Early diagnostic biomarker	^372^
	miR-141	Serum	up		Early diagnostic biomarker	^373^
	miR-151b	Serum	up		Prognostic biomarker	^374^

## Renal cell carcinoma (RCC)

RCC is one of the most common malignant tumors which is the main type of kidney cancer. It is difficult to diagnose RCC in its early stages and is now prone to recurrence after surgery as well as radiotherapy, hence earlier diagnosis of RCC is required. RCC cancers take a lengthy period (up to 50 years) to evolve from their initial genetic changes to clinical symptoms. Although little histologic modifications are detectable in the comparable histologically normal renal tissues of individuals with renal tumors, epigenetic alterations have accumulated in this noncancerous renal tissue, indicating their potential application in early identification by liquid biopsy.^296^ Liquid biopsy can be used as an auxiliary test for early diagnosis of RCC, and the main biomarkers include CTCs, ctDNA, miRNA, and so on.^297^

In the early diagnosis of RCC, the detection of CTCs is less frequently concerned. And CTCs were detected in 100% of samples evaluated in patients with metastatic clear cell renal cell carcinoma (ccRCC) but not in healthy controls.^298^ In addition, in the differential diagnosis of RCC, ctDNA plays a role in detecting its methylation, and plasma cfDNA has been found to have 300 differentially methylated regions, which is effective in the diagnosis of RCC by detecting the methylation.^299^ Studies on miRNAs as liquid biopsy markers have shown that the combination of multiple miRNAs has high sensitivity and specificity in the diagnosis of RCC and helps to differentiate it from benign renal tumors.^300–302^ For example, four microRNA (miR-21-5p, miR-150-5p, miR-145-5p, and miR-146a-5p) panels were produced, and the AUC of the panels was 0.938 (95% CI: 0.889–0.971; sensitivity: 90.79%, specificity: 93.75%).^300^ Similar to ctDNA, methylation of miRNAs is also beneficial for the diagnosis and differentiation of RCC.^303^ In liquid biopsy of RCC, some scholars have found that in addition to CTCs, cfDNA, and cfRNA, some other biomarkers are also involved in the diagnosis of RCC, such as some metabolites, plasma proteins, and other biomarkers, which are also involved in the diagnosis of RCC, but there are fewer research reports, that require more in-depth exploration.^304–307^

Additionally, CK^+^CTCs are frequently detected and the number of them correlates with disease progression.^298^ miRNAs have been found to be associated with the grading and staging of RCC as well as distant metastasis. There are increased serum miR-122-5p and miR-206 levels in patients with metastatic diseases. In addition, miR-122-5p levels were associated with grade.^302^

More studies have found that liquid biopsy can be used for the treatment monitoring and prognosis prediction of RCC. With the development of genetic testing technology, studies on ctDNA and miRNA have been more focused in liquid biopsy. First, cfDNA content and fragment length play a role in prognostic prediction of RCC, with shorter cfDNA fragments significantly associated with shorter PFS and postoperative ctDNA associated with prognosis only in patients with metastatic RCC but not in those without metastasis.^308–310^ In the case of ctDNA, mutations in ctDNA continue to be of great interest in liquid biopsies of RCC, with several studies detecting a variety of mutations in ctDNA and miRNA. For ctDNA, its mutations remain of great interest in liquid biopsies of RCC, and several mutation sites have been detected in several studies, including VHL, brca1-associated protein 1 (BAP1), recombinant polybrominated gene 1 (PBRM1), TP53, ATM, and others, with the most common mutated genes being VHL.^308,310–312^ Its mutations correlate with prognosis, e.g., patients with high cfDNA concentrations and TP53 mutations have the worst PFS, whereas patients with low cfDNA and no mutations in TP53 have a longer PFS (*p* = 0.004).^310^ Mutation detection of ctDNA helps to predict the efficacy of ICI and TKI therapy, and the frequency of ctDNA mutations is significantly reduced after surgery.^312^ miR-15a has been regarded as a possible key molecule for liquid biopsy of RCC because it not only identifies benign tumors as well as RCC but also correlates with RCC postoperative prognosis(specificity:98.1%,sensitivity:100%, AUC: 0.955).^313^ The number of mixed CTCs in the metastasis and no-metastasis groups at 12 months postoperatively was significantly different from the number of mixed CTCs preoperatively, suggesting that the risk of recurrence or metastasis correlates with dynamic changes in the count of CTCs.^314^ Given that miRNAs are more stable in the exosomes, many studies have begun to target miRNAs in the exosomes, and a great deal of potential exists for their clinical application.^315,316^ Moreover, some circRNAs, lncRNAs, and piRNAs have been considered for liquid biopsy in RCC.^317–320^

## Bladder cancer (BLCA)

Bladder cancer is a highly heterogeneous malignancy. BLCA can present as non-muscle-invasive bladder cancer (NMIBC), muscle-invasive bladder cancer (MIBC), or metastatic disease events, each characterized by distinct molecular drivers.^321^ Currently, invasive cystoscopy and tissue biopsy remain the gold standard for BLCA identification and surveillance. However, this method suffers from drawbacks such as sampling bias, invasiveness, and difficulty in sampling deep tumors, which limits its use in mass screening.^322^

In the early diagnosis of BLCA, CTCs and miRNA are the main liquid biopsy markers. In BLCA, CTCs can be quantified by detecting folate receptor-alpha and can be diagnostic for BLCA (sensitivity: 82.14%, specificity: 61.9%).^323^ The role of ctDNA methylation in liquid biopsy has received much attention. Various ctDNAs such as p16 DNA, APC, GSTP1, TIG1, etc. have been shown to be hypermethylated in patients with BLCA,^324,325^ and there is a positive correlation between the frequency of methylation and the stage, so the methylation of ctDNA may be used as a biomarker for the diagnosis of BLCA.^326,327^ miRNAs in plasma and exosomes have been widely studied as potential biomarkers and therapeutic targets. Firstly, in blood, miR-19a, miR-99a, miR-200b, miRNA-373, and other miRNAs have been shown to be expressed differently in the blood of BLCA patients than in healthy people, which is a potential biomarker for BLCA.^328,329^ When multiple miRNAs are integrated for combined diagnosis, they show high accuracy in early diagnosis and differential diagnosis of BLCA.^330,331^ A study has constructed logistic regression modeling that predicts diagnosis with 89% accuracy in detecting the presence or absence of BLCA, 92% accuracy in distinguishing invasive BLCA from other cases, and 100% accuracy in distinguishing MIBC from controls.^329^ In exosomes, miRNAs have been found to be associated with tumor progression and metastasis, and similar to the miRNA alterations detected in the bloodstream, exosomal miRNAs also play a role in the diagnosis of BLCA and in predicting the prognosis.^332,333^ Compared to blood, urine testing is mainly focused on protein and exfoliative cytology. Most of the proteins are detected by ELISA, such as the expression of BLCA-4(sensitivity: 93%, specificity: 97%, AUC: 0.9607), MCM5(sensitivity: 75.6%, specificity: 71.1%), etc., to assist in the early diagnosis of BLCA.^334,335^ On the other hand, for exfoliated cells, not only cell surface markers, such as Cytokeratin 17, can be used to identify tumor cells for the diagnosis of BLCA,^336^ but exfoliated cell DNA, including TERT promoter mutations(specificity: 100.00%, sensitivity: 46.67%) and FGFR3 mutations, are the most common mutations in somatic cells, which can be used to detect BLCA noninvasively and to monitor recurrence.^337,338^

For the detection of tumor progression, the presence of CTCs has also been associated with metastasis of BLCA, and CTCs have been shown to predict metastasis in NMIBC and to identify those at high risk of recurrence.^339–341^ Also, in MIBC, there is a higher level of CTCs, again demonstrating the correlation between CTCs and tumor muscle infiltration.^339^ Although ctDNA is rarely expressed in blood, it has been found to be superior compared to histology in reacting with advanced tumor load, for example. And plasma ctDNA has a high concordance with genes detected in tumor tissue.^342^

Liquid biopsies can also be used for therapeutic monitoring and prognosis prediction in BLCA. CTCs has been shown to be associated with disease recurrence and poor prognosis in several studies. After clinical treatment, CTCs-positive patients have worse progression-free survival, CSS, and OS (sensitivity: 35%, specificity: 97%).^340,343,344^ CTCs can be used to assess the efficacy of cisplatin-based chemotherapy, PDL1 immunotherapy, etc., and can help to better predict the efficacy of treatments.^345,346^ CTCs-positive patients have higher rates of cancer-related mortality and disease recurrence compared to CTCs-negative patients. And CTCs-positive patients who received neoadjuvant chemotherapy (*n* = 22) survived longer than those who were not CTCs-positive (*n* = 48).^345^

Mutations of ctDNA are another concern in addition to the DNA methylation hotspot. A variety of genetic mutations have been found to be present in the blood of BLCA patients with potential as prognostic markers. For example, FGFR3 and PI3KCA mutations are significantly associated with recurrence of the disease, and the number of genomic alterations has been correlated with response to immunotherapy.^347–349^ A clinical trial has found a strong correlation between ctDNA Variant Allel Frequency (VAF) and treatment duration, clinical activity, PFS, and OS. Compared with patients with dVAF ≥ 0, patients with lower mean VAF had a significantly better PFS and OS.^348^

In addition to this, ctDNA expression levels are a valid indicator, and it has been demonstrated that liquid biopsy results are detected earlier than imaging at the time of tumor recurrence, thus making ctDNA a potent prognostic marker for the patients.^350^ Whether it is chemotherapy, radiotherapy, immunotherapy, or cystectomy, ctDNA testing can respond to disease progression after treatment as well as detect treatment efficacy, suggesting that it may be possible to improve the treatment regimen for better therapeutic efficacy by continuous monitoring of ctDNA.^351–353^

However, compared to blood monitoring, although significant progress has been made in urine biomarkers and urocytology monitoring, their sensitivity and specificity are low, and thus their application in low-grade tumors is partially limited.^322,354^

## Wilms’ tumor (WT)

Wilms’ tumor (WT), which often occurs in children, is the main type of renal tumor in children and currently has a recurrence rate of up to 15%.^355^ The majority of cases of WT are disseminated, caused by mutations in somatic cells that are often limited to tumor tissue, and the tumors are very genetically heterogenous.^356^ Liquid biopsy may play a role in its early screening as well as therapeutic monitoring, which may help in tumor treatment as well as reducing recurrence.

In blood, liquid biopsies are performed mainly by monitoring miRNA and ctDNA. Among them, several miRNAs have been found to serve as markers for WT diagnosis and to play a role in the differential diagnosis of WT from other tumors (accuracy: 97.5%, sensitivity: 99.8%, specificity: 94.7%).^357^ Mutations in ctDNA have been found to allow for the early identification of WT and the OS of ctDNA-positive patients is poorer than that of ctDNA-negative patients.^358,359^ Urine, as the other main sample for liquid biopsy, is less effective in the diagnosis and differentiation of WT than blood. For example, ctDNA was detected in the serum for 82% patients, but in the urine for 26% patients.^359^ However, proteomic monitoring of urine specimens acts as an important class of molecular markers in liquid biopsies. For example, neuron-specific enolase, basic fibroblast growth factor (bFGF), and hyaluronidase are enriched in the urine of patients with nephroblastoma and can be used as indicators for the diagnosis of WT.^360,361^ In addition, in the monitoring of the therapeutic efficacy of WT as well as in the determination of the prognosis, several protein biomarkers, such as transgenic specific enolase (NSE), hyaluronic acid (HA), hyaluronan-stimulating activity (HSA), and hyaluronidase, etc. can be used as assays to assist in the judgment.^360,362^

## Uroepithelial carcinoma (UC)

Uroepithelial carcinoma (UC) can be divided into uroepithelial bladder cancer (UBC) and upper uroepithelial carcinoma (UTUC), UBC has been introduced in detail in the section of BLCA, so this section will mainly focus on UTUC. UTUC is known to be a particularly aggressive form of uroepithelial carcinoma, usually diagnosed at an advanced stage and posing serious therapeutic difficulties due to its anatomical location and the potential for early lymphatic and hematogenous dissemination.^363^

The main biomarkers for liquid biopsy in UTUC are ctDNA, miRNA, protein, etc. For ctDNA, the main focus is on its fragment size, mutation, and methylation. Plasma cfDNA fragment size correlates with UTUC and may be helpful in the diagnosis of UTUC (AUC: 0.72).^364^ Monitoring of ctDNA mutations and methylation in urine has revealed that both can diagnose UTUC (sensitivity: 96%, specificity: 88%),^365–367^ and predict the prognosis of the patients(sensitivity: 86.5%, specificity: 94.7%).^368–370^ Moreover, the combined detection of methylation and mutation can better monitor UTUC with higher sensitivity and specificity (sensitivity: 94.0%, specificity: 93.1%, AUC: 0.96).^371^ Multiple miRNAs have been identified as diagnostic markers for UTUC, and some miRNAs were found to be predictive of UTUC prognosis.^372–374^ For example, miR-151b was able to differentiate between two groups of UTUC patients with significant differences in tumor progression probability (*p* = 0.006) and cancer-specific survival probability (*p* = 0.034).^374^ In addition, a variety of proteins in plasma and urine may be useful for the detection of UTUC. For example, plasma phosphorylated protein 1 and urine FXYD3 can effectively identify patients with early UTUC, facilitating rapid screening for UC.^375,376^ Also, the survival prognosis of UTUC patients can be predicted by proteins, such as albumin-globulin ratio (AGR) and hemoglobin levels.^377^ Certain proteins, such as serum iron-regulated proteins and GDF-15 levels, have been associated with the progression and invasion of UTUC.^378^

## Circulatory system

In the circulatory system, we will mainly discuss the application of liquid biopsies in myelodysplastic syndromes (MDS)/acute myeloid leukemia (AML), lymphomas, and multiple myeloma (MM) (Table 10).

**Table 10:** Liquid biopsy in circulatory system cancers

Cancer	Liquid biomarker	Origin	Tendency	Downstream target	Function	Reference
MDS/AML	ctDNA	Plasma	up		Disease progression biomarker	^382^
	ctDNA	Serum	up		Prognostic biomarker	^389^
	cfDNA	Plasma	up		Disease progression biomarker	^383^
	cfDNA mutation	Plasma, Serum	up		Disease progression biomarker	^385^
	ctDNA methylation	Peripheral blood	up		Early diagnostic biomarker, Efficacy monitoring biomarker	^384^
Lymphoma	ctDNA	Serum	up		Disease progression biomarker, Efficacy monitoring biomarker	^392^
	ctDNA mutation	Peripheral blood	up		Disease progression biomarker	^395^
	ctDNA	Peripheral blood	up		Early diagnostic biomarker, Efficacy monitoring biomarker	^396^
	ctDNA mutation	Peripheral blood	up		Efficacy monitoring biomarker	^397^
	ctDNA	Plasma	up		Efficacy monitoring biomarker	^399^
MM	CTCs	Peripheral blood	up		Early diagnostic biomarker	^403^
	ctDNA	Plasma	up		Disease progression biomarker	^407^
	cfDNA	Plasma	up		Early diagnostic biomarker	^408^
	ctDNA mutation	Serum	up		Disease progression biomarker	^405^
	FGFR3, KMT2C, MAML2, ZFHX4 mutation	Peripheral blood	up		Efficacy monitoring biomarker	^409^

## Myelodysplastic syndromes (MDS)/acute myeloid leukemia (AML)

Because of the analogous mutation profiles of MDS and AML genes and the characteristic transformation of MDS to AML, which occurs in ~30% of MDS, we have discussed MDS/AML jointly when discussing liquid biopsies of circulatory tumors. The reason why ctDNA is able to dominate liquid biopsies of MDS/AML is due to the influence of the following conditions. There are multiple genetic mutations in MDS/AML, and the correlation between ctDNA in the blood and the variants detected by bone marrow biopsy is high.^379,380^ In the plasma of AML patients, sequence variants and copy number variants of ctDNA are highly consistent with the results of the bone marrow biopsy.^379,381^ Also, due to the inherent heterogeneity of the tumors, the full information about the tumors may not be obtained at the time of the bone marrow puncture. The ctDNA test can be used to detect chromosomal aberrations and to detect the course of the disease in patients with MDS.^382^

Studies have shown that the concentration and integrity of DNA in patients with acute leukemia are higher than in healthy controls, and that relapse is associated with a significant increase in DNA integrity, so plasma DNA integrity may be a potential biomarker for detecting leukemia progression.^383^ Methylation of ctDNA has also received attention. AML can be reliably distinguished from the healthy population by detecting ctDNA methylation in the peripheral blood of AML patients.^384^

In addition, ctDNA mutations have been associated with the detection of therapeutic response. ctDNA in the blood of patients with MDS dynamically responds to the tumor load during treatment and demonstrates mutations and karyotypic abnormalities in MDS. Thus, ctDNA may respond to dynamic changes in myelogenetic abnormalities.^385^ Several studies have synthesized and discussed the detection of ctDNA in the plasma of patients with MDS and AML.^386,387^ After demethylation therapy or chemotherapy, ctDNA was detected in the peripheral blood of patients in complete remission/complete rheological recovery (CR/CRi) with a lower mean number of VAFs, and mutations were negatively correlated with longer progression-free survival (PFS) and overall survival (OS).^388^ And ctDNA methylation has been shown to correlate with tumor status after treatment. After treatment with azacitidine, AML patients showed a rapid decrease in peripheral blood ctDNA methylation levels.^384^

And for tumor recurrence, the rate of recurrence is higher in ctDNA-positive patients than in negative patients.^389^ As MDS progresses to AML, mutations in ctDNA can be detected in plasma earlier than changes in cell morphology.^388^

## Lymphoma

Diffuse large B-cell lymphoma is the most common lymphoma and is an aggressive heterogeneous lymphoma. Multiple studies have shown that ctDNA can detect lymphoma-associated genetic alterations with 95% concordance with tissue biopsy results.^390,391^ CtDNA has been shown to be useful for disease progression and predicts progression (positive predictive value of 88.2% and negative predictive value of 97.8%) earlier than conventional imaging.^392^ CtDNA mutations in plasma are significantly downregulated after treatment,^392,393^ and their mutations are associated with event-free survival (EFS) and OS.^394^ To further improve the sensitivity of the analysis, ctDNA detection methods are being actively investigated, including phase variant enrichment and detection sequencing, CAPP-seq, and others.^391,393,395^ In Hodgkin’s lymphoma (HL), it has been demonstrated that ctDNA levels in HL plasma are higher than in the healthy population,^396^ and have correlated with radiologically detected findings, i.e., tumor volume.^397,398^ Its plasma ctDNA has been determined to be a reliable source of tumor DNA for identifying mutations in HL.^397,399^ During the research process of ctDNA mutations, it has been found that ctDNA mutation correlates with efficacy testing and prognosis of various therapies, including chemotherapy, immunotherapy, etc.^399–401^ Plasma ctDNA concentration before treatment could independently predict clinical outcome, and patients with particularly poor prognosis after radical immunochemotherapy could be identified by plasma ctDNA monitoring during treatment.^400^ In combination with PET/CT, ctDNA levels were found to correlate with total metabolic tumor volume detected by PET/CT,^398^ and ctDNA values were correlated with disease progression and survival.^399^ Therefore, the mutual complementarity of the two assays is conducive to a more accurate determination of treatment efficacy and risk of recurrence, etc.

## Multiple myeloma (MM)

MM is characterized by an intricate array of genetic and epigenetic alterations that result in the malignant conversion of plasma cells. It is a hematologic disease that cannot be cured and exhibits significant variation in both space and time.^402^ Detection of CTCs in the serum of MM patients revealed that CTCs were higher in MM patients. Mutation detection of CTCs showed good agreement in the degree of mutation matching between MM cells in BM and CTCs in blood, and was 95% concordance in copy number alterations at the chromosome arm lFel.^403,404^ And in extramedullary (EM) plasmacytoma samples, an 87% concordance was found between the mutational profiles of EM tumor cells and CTCs, with the highly concordant mutations suggesting that CTCs may be responsible for the development of EM.^403^ Despite the good concordance of mutations in CTCs and MM cells, there are still some inconsistencies, which may indicate that a combination of the two tests may be more helpful in the diagnosis of MM. CfDNA is the most commonly used marker for MM liquid biopsy. It was found that changes in ctDNA levels preceded those of other serum markers, such as FLC.^405,406^ Therefore, ctDNA may be an earlier predictor of disease progression like CTCs. In addition, ctDNA can also be used to detect the progression of MM. Firstly, ctDNA levels were detected to show a sustained elevation during MM progression and the number was correlated with tumor load parameters such as the percentage of infiltration with bone marrow plasma cells.^407,408^ Moreover, serial analysis of the mutational status of cfDNA helps to assess the efficacy of treatment, and because EM is difficult to perform routine biopsies, mutation detection of ctDNA may be more of an examination advantage in the diagnosis as well as prognostic assessment of patients with EM.^407^ Studies have been conducted on cfDNA mutations and drug therapy, and it was found that ctDNA mutations such as DIS3, FGFR3, KMT2C, MAML2, and ZFHX4 mutations may predict resistance to certain therapies.^409^ Recently, a study demonstrated that the detection of ctDNA mutations to select the corresponding selective inhibitors for treatment has promising efficacy,^410,411^ further suggesting that liquid biopsy has great potential for use in the diagnosis and treatment of MM.

## Endocrine system

The application of liquid biopsy in endocrine system tumors mainly includes thyroid cancer (TC) and breast cancer (BRCA) (Table 11).

**Table 11:** Liquid biopsy in endocrine system cancers

Cancer	Liquid biomarker	Origin	Tendency	Downstream target	Function	Reference
TC	CTCs	Peripheral blood	up		Early diagnostic biomarker	^421^
	CTCs	Peripheral blood	up		Efficacy monitoring biomarker	^422^
	ctDNA mutation	Plasma	up		Efficacy monitoring biomarker	^425^
	ctDNA	Plasma	up		Disease progression biomarker	^424^
	BRAFV600E mutation	Plasma	up		Efficacy monitoring biomarker	^426^
	RET M918T mutation	Plasma	up		Prognostic biomarker	^427^
	BRAF(T1799A) mutation	Plasma	up		Disease progression biomarker	^428^
	*PIK3CA mutation*	Peripheral blood	up		Prognostic biomarker	^429^
	SLC5A8, SLC26A4 methylation	Plasma	up	BRAF	Early diagnostic biomarker	^418^
	ctDNA methylation	Serum	up		Early diagnostic biomarker, Tumor recurrence biomarker	^430^
	miR-146b-5p, miR-21a-5p	Plasma exosome	up		Early diagnostic biomarker	^420^
	miR-29a	Serum exosome	down		Early diagnostic biomarker, Prognostic biomarker	^431^
BRCA	CTCs	Peripheral blood	up		Prognostic biomarker, Efficacy monitoring biomarker	^452^
	TP53, PIK3CA, ESR1 mutation	Plasma	up		Efficacy monitoring biomarker	^436^
	ctDNA mutation	Plasma	up		Efficacy monitoring biomarker, Tumor recurrence biomarker	^437^
	PIK3CA mutation	Plasma	up		Early diagnostic biomarker, Efficacy monitoring biomarker, Tumor recurrence biomarker	^438^
	miR-1246, miR-21	Plasma exosome	up		Early diagnostic biomarker	^441^
	miR-101, miR-372	Serum exosome	up		Early diagnostic biomarker	^442^
	miR-106a-3p, miR-106a-5p, miR-20b-5p, miR-92a-2-5p	Serum exosome, Plasma exosome	up		Early diagnostic biomarker	^443^

## Thyroid cancer (TC)

TC is the most common endocrine malignancy. The majority of this tumor originates from epithelial tissue and includes differentiated thyroid carcinoma (DTC), poorly differentiated thyroid carcinoma (PDTC), anaplastic thyroid cancer (ATC).^412^ The differentiated type of TC, which usually exhibits an indolent clinical behavior and has a good prognosis, can be further classified into papillary thyroid carcinoma (PTC) (85–90%), follicular thyroid carcinoma (FTC) (5–10%), and Hürthle cell carcinoma (HCTC) (3%) cancers.^413,414^ The incidence of TC has been increasing globally over the past 30 years.^412^ Currently, rapidly evolving liquid biopsy techniques offer unique advantages in the diagnosis and prognostic testing of this disease. The following section focuses on liquid biopsy techniques related to TC.

First, for the detection of CTCs, in addition to enrichment based on its physical properties, immunoaffinity-based enrichment methods are now more commonly used, including assays for anti-EpCAM, tumor-specific cell surface antigens, cytokeratins (CKs), and other stem cell or mesenchymal markers.^415,416^ Of these, the CellSearch® method is currently the only method that is method authorized by the FDA to monitor the number of CTCs in blood samples.^34^ It has been shown that the number of CTCs is significantly increased in patients with DTC and that their number is proportional to the tumor stage at diagnosis, suggesting that CTCs may have a strong correlation with this tumor. CTCs values were significantly higher in TC patients compared to controls and the number of CTCs correlated with initial tumor stage.^417^ In addition to this, methylation of ctDNA has been recognized as a promising biomarker. One study found that the SLC5A8 and SLC26A4 genes have higher methylation expression in patients with TC.^418^ It has been shown that exosomal miRNAs are promising diagnostic markers for TC, being more resistant to the proteolytic activity of RNAases and more stable than free miRNAs in body fluids.^419^ Therefore, the detection of miRNAs in the exosomes seems to be more relevant. It was found that miR-146b-5p and miR-21a-5p were significantly elevated in the cellular exosomes of PTC patients compared to patients with benign multinodular disease, but no significant difference was found when free miRNAs in the blood were analyzed.^420^ This further demonstrates the superiority of exosomal miRNA detection testing for early diagnosis of TC.

Also, various liquid biopsy markers can be used to detect TC progression as well as treatment prognosis. And in the FTC typing of DTC, a research team found that CTCs was more common in malignant patients,^421^ while all benign patients were negative (specificity 100%, sensitivity 46%). So, CTCs may also be used as an indicator to judge the benignity or malignancy of a tumor. Furthermore, in a study by Qiu et al., it was found that metastasis was more likely to occur when the number of isolated CTCs was 5 or more (sensitivity: 64.3%, specificity: 83.8%), whereas isolation of seven or more CTCs predicted a poor prognosis in response to radioiodine treatment (sensitivity: 73.7%, specificity: 69.6%).^422^ Some studies have found that in the metastatic PTC patients mutations in RET and BRAF genes are more common and predict poor prognosis.^423^ Also, mutations in NRAS and TP53 were found to possibly accelerate tumor progression in DTC and ATC patients.^424^

In addition, ctDNA is significantly better than other imaging tests and protein marker tests in predicting the prognosis of TC. Several studies have shown that the detection rate of specific gene mutations in cfDNA is associated with overall survival and poor prognosis of TC, such as PIK3CA, BRAFV600E, RETM918T, and BRAFT1799A.^424–428^ Mutations in cfPIK3CA are also associated with poor prognosis in patients with ATC.^429^ Another study found that patients with recurrent TC have a higher positive rate (70%) of serum DNA methylation.^430^ Similarly, one study testing CTCs in patients with medullary thyroid cancer (MTC) both preoperatively and postoperatively, there were similar conclusions, which suggest that CTCs may be a good prognostic monitoring indicator.^425^ The same can be said for serum exosomes in predicting patient prognosis. One study found that PTC patients with high serum exosomal miR-29a levels had significantly greater OS and RFS than those with low exosomal miR-29a expression levels, suggesting that exosomal miR-29a levels may be associated with PTC recurrence.^431^ In addition, miR-146b, and miR-222 have also been shown to be potential markers of PTC recurrence.^432^ And a large number of miRNAs have also been found and reported, such as miR-16-2-3p, miR-223-5p, miR-130a-3p, miR182-5p, etc. have expressed their advantages in different aspects, which can provide some references for the diagnosis of clinical diseases.^433^

## Breast cancer (BRCA)

Breast cancer (BRCA) is one of the most common malignant tumors among women in China, and its incidence rate has long been in the first place with the leading cause of tumor death among middle-aged women.^434^ Early screening and early diagnosis of this disease are extremely important because early metastatic cases are curable, while distant metastatic cases are currently considered incurable. Tissue biopsy and immunohistochemistry, the gold standard techniques for conventional BRCA treatment, have limited detection rates because of the high heterogeneity of the tumor, while liquid biopsy has unique advantages in this area, which provides sustainable and personalized medical treatment for patients.^435^

CtDNA has become a hotspot for scientists because of its high ability to assess tumor heterogeneity.^435^ Point mutations are one of the more common types of mutations in ctDNA, with genes such as TP53, PIK3CA, and ESR1 being the hotspot mutated genes in BRCA identified to date.^436,437^ By evaluating PIK3CA mutations in the plasma of BRCA patients, Beaver et al. found that the sensitivity of this method for detecting early BC was 93.3%, and the specificity of this gene reached 100%.^438^ Meanwhile the gene has been approved as a biomarker for the Pl3K inhibitor alpelisib in Europe and the United States.^439^ In addition to this, ctDNA methylation can also be used for early diagnosis as well as differentiation of breast cancer. A study has shown that the detection of ctDNA methylation biomarkers is highly accurate in the early diagnosis of breast cancer patients (AUC: 0.889, sensitivity: 100%, specificity: 75%).^440^ It has been reported that the expression levels of plasma exosomes miR-21(AUC:0.69), miR-1246(AUC:0.69), and serum exosomes miR-101, and miR-372 are significantly elevated in BRCA patients, suggesting that the above exosomal miRNAs could be used as a potential biomarker for the diagnosis of early-stage BRCA.^441,442^ By comparing the expression levels of circulating exosomal miRNAs in blood samples from 32 healthy volunteers and 32 BRCA patients, Li et al. found that the levels of four exosomal miRNAs (miR-106a-5p, miR-19b-3p, miR-20b-5p, and miR-92a-3p) were significantly elevated in sera of BRCA patients (sensitivity: 87%, specificity: 89, AUC: 0.937), and found that three plasma-derived exosomal miRNAs (miR-106a-3p, miR-106a-5p and miR-92a-2-5p) levels were elevated compared to healthy volunteers(sensitivity:82%, specificity:79%, AUC:0.889).^443^ It is suggested that the above seven circulating exosomal miRNAs are expected to be biomarkers for early diagnosis of BRCA. Among them, the expression of miR-106a-5p was higher than that of healthy volunteers both in plasma and serum, suggesting that exosomal miR-106a-5p has the potential to be a specific biomarker for early diagnosis of BRCA. In a recent study, a droplet digital ExoELISA method was developed for the detection of GPC-1(+) exosomes in clinical samples from healthy individuals, patients with benign breast cancer, patients with breast cancer, and post-breast cancer patients. The microtitre digital ExoELISA method demonstrated unprecedented accuracy and high specificity in exosome quantification with a detection limit of at least 10 exosomes per microliter. The results showed significantly higher GPC-1 expression in tumor-derived exosomes compared to normal and benign breast disease samples, and higher levels of GPC-1(+) exosomes in breast cancer patients than in healthy controls and patients with benign breast disease.^444^ For CTCs detection, the most effective CTCs test for BRCA is still the FDA-approved CellSearch® method.^445^ This method is an automated immunomagnetic enrichment method based on the EpCAM, which has a high degree of sensitivity and specificity. In addition, chip-based microfluidic methods are currently being developed.^446^ The main principle is to retain and isolate CTCs based on their different sizes and deformability from other blood components as blood flows within a microfluidic chip.^447^ It has been reported that the number of CTCs is positively correlated with the tumor stage,^448^ and that when CTCs are counted at a higher number, the worse the prognosis of the patient is,^30^ and the more prone to metastasis, which suggests that CTCs is a good prognostic indicator. According to the NCCN Clinical Practice Guidelines in Oncology, Version 3.2022, patients who have continuously increased CTCs levels following three weeks of first-line treatment have worse OS and PFS.^449^ A research team dynamically monitored ctDNA, CTCs, and CA15-3 levels in 30 patients with advanced BRCA. The results showed that changes in patient-specific ctDNA levels during treatment correlated most strongly with efficacy, and had higher sensitivity (90%) compared with CA15-3 and CTCs.^450^ Moreover, increased ctDNA levels indicated disease progression on average 5 months earlier than clinical imaging.^450^ In addition, a variety of exosomal miRNAs are associated with an increased risk of BRCA development and shorter survival. In addition, it has been found that genes such as miR-23a, miR-5100, miR-19b-3p, and miR-21 are involved in the process of EMT,^451^ which is also of clinical significance for the prognostic detection of tumors. We can also detect the number of CTCs in patients after taking treatment to reflect the clinical efficacy. For example, Nakamura et al. analyzed the relationship between the effect of chemotherapy and the change in the number of CTCs in metastatic BRCA and found that after 1 cycle of chemotherapy, the number of CTCs decreased by more than 90% compared with the number of CTCs before chemotherapy, 85.7% of the patients were in complete remission or partial remission; and for the patients who did not have a decrease or even an increase in the number of CTCs after chemotherapy, there were 63.6% of the patients who had progression of the disease.^452^ According to the Chinese Society of Clinical Oncology’s (CSCO) Breast Cancer Guidelines 2022, CTCs can partially mimic solid tumors and be employed in addition to genetic sequencing, pathological diagnosis, and disease surveillance.^453^ For patients with advanced BRCA, ctDNA is a sensitive and specific biomarker for monitoring tumor load, and changes in its levels suggest changes in tumor load and thus reflect drug efficacy. Although ctDNA has made large and substantial advances in BRCA and the applications of ctDNA are expanding,^454^ the information provided by ctDNA is usually limited to the assessment of disease burden and the presence or absence of genomic mutations. Further applications and tests remain to be explored.

In addition, lncRNAs and circRNAs have the potential to be diagnostic and prognostic liquid biopsy biomarkers for BRCA. LncRNAs can regulate transcription by binding to enhancer regions. LncRNAs are also involved in the binding of HOTAIR genes to the histone modification complexes PRC2 and LSD1, promoting H3K27 histone methylation and H3K4 demethylation, leading to target gene shutdown and promoting BRCA metastasis.^455^ As for circRNAs, their high stability, abundance, and specific expression make them have considerable clinical potential as new biomarkers. They are mainly involved in the regulation of cell survival, proliferation, and invasion through the MAPK/AKT signaling pathway. Some studies have found that in tumors hsa_circ_0017650 and hsa_circ_0017536 are less expressed in tumor tissues, and it is hypothesized that these two circRNAs may have a tumor suppressor effect in BRCA.^456^ In addition to the above conventional liquid biopsy techniques, other biomarkers for different manifestations of tumors are also developing, such as exosomal phosphorylated proteins, DNA methylation products, etc. The most appropriate liquid biopsy method for different manifestations of tumors can be adopted in order to provide an early reference for the diagnosis of the disease.

## Locomotor system

Primary malignant bone tumors have a high morbidity and mortality rate in children and adolescents, so accurate and effective screening and diagnostic tools are particularly important. However, the invasive nature of routine tissue biopsy makes it impossible to repeat the sampling during patient prognostic monitoring, and patients are unable to receive sustainable precision medicine. Liquid biopsy, which is currently in the spotlight, overcomes the shortcomings of tissue biopsy and provides a new way of thinking for the diagnosis and prognostic monitoring of malignant tumors. Next, we mainly focus on several common bone tumors, including osteosarcoma (OS), chondrosarcoma (CS), and ewing’s sarcoma (ES), to provide a brief introduction to liquid biopsy techniques. However, since studies related to osteosarcoma and chondrosarcoma are also extremely limited, this section focuses on ewing’s sarcoma (Table 12).

**Table 12:** Liquid biopsy in motor system cancers

Cancer	Liquid biomarker	Origin	Tendency	Downstream target	Function	Reference
	*EWSR1 Translocation*	Plasma	up		Efficacy monitoring biomarker	^460^
	ctDNA	Plasma	up		Prognostic biomarker	^462^
	EWSR1 fusion sequence	Plasma	up		Efficacy monitoring biomarker	^458^
	miR-34b	Plasma	down		Tumor aggressiveness biomarker	^472^
	miR-143/145	Plasma	down	FSCN1	Early diagnostic biomarker	^473^
	miR34a	Plasma	down		Disease progression biomarker, Efficacy monitoring biomarker	^475^

For bone tumors of mesenchymal origin, the ability of conventional assays to detect CTCs is extremely limited due to the obvious tumor heterogeneity and the lack of classical tumor markers, and there is also a lack of relevant studies. However, in a recent study, a team confirmed the presence of CTCs in ES patients based on the immunodetachment of CD99^+^ tumor cells and magnetic beads, followed by molecular analysis to detect specific fusion transcripts from chromosomal translocations,^457^ which provides a new way of detecting CTCs. Nevertheless, the potential clinical significance of CTCs is unclear and needs to be further explored. In patients with ES, the expression of CD99^+^ and the presence of chromosomal translocations are two of the most important features of the tumor.^457^ In particular, the detection of fusion genes such as EWSR1-FLI1, which is frequently present, is the gold standard for the diagnosis of ES and can be detected using genomic fusion sequences.^458^

More studies focus on detection of tumor progression and prediction of prognosis. The detection of ctDNA also has certain clinical significance, as some researchers have analyzed the ctDNA levels of IDH1 mutants in preoperative and postoperative patients with CS and found that the levels of ctDNA were correlated with different tumor grades, and a significant decrease in ctDNA levels was found in most patients who underwent surgical resection.^459^ While in OS patients, ctDNA levels also underwent similar changes after treatment.^460,461^ In addition, an increase in chromosome 8q has been observed in the ctDNA of patients with OS, which may be associated with a poorer prognosis.^462^ In addition, tumor cell exosomes have received much attention because they carry some of the functional proteins and genes of their “parent” tumor cells and may also play an important role in the pathogenesis, diagnosis, and treatment of primary bone tumors. Micro-fractionated ultracentrifugation is one of the most widely used methods in routine assays and is also a gold standard for exosome isolation.^463^ In the past few years, many new integrated microfluidic platforms have been developed for analyzing exosome levels, quantifying disease-specific subpopulations, and characterizing exosomal proteins and RNA at the histological level.^464–467^ Compared to conventional methods, emerging microfluidic platforms significantly reduce sample volume, reagent consumption, and separation time, while greatly improving separation recoveries and exosome quality levels for higher specificity.^468,469^ However, research in this area is still in its infancy and has great potential for development. Especially for osteosarcoma (OS), miRNA is highly suggestive of disease staging, metastasis, and therapeutic efficacy.^470^ As well, most of the studies to date have focused mainly on miRNA of OS, followed by CS and ES, for example, it was found that high expression of miR-135b, miR-150, miR-542-5p, and miR-652 may be associated with the onset and progression of OS.^471^ While down-regulation of miR-34b may be associated with metastasis of tumors in OS patients. Compared with non-metastatic patients, miR-34b plasma expression levels were significantly lower in metastatic patients.^472^ In CS, multiple miRNAs such as miR-20, miR-96, miR-100, miR-125b, miR-136 and other genes have significantly altered expression levels in both cell lines and tumor samples.^473^ And in a recent study, 17 key miRNAs were found to be involved in regulating the formation and growth of chondrosarcoma.^474^ Also, miR34a was found to be a potential biomarker for the development of ES.^475^ And miR-185 was shown to be involved in the formation and survival of ES cells.^476^

## Reproductive system

In this section, we focus on the application of liquid biopsy in the following five reproductive system tumors, including cervical cancer (CC), endometrial carcinoma (EC), ovarian cancer (OC), prostate cancer (PCa), seminoma (Table 13).

**Table 13:** Liquid biopsy in reproductive system cancers

Cancer	Liquid biomarker	Origin	Tendency	Downstream target	Function	Reference
Cervical cancer	CTCs	Peripheral blood	up		Prognostic biomarker	^490^
	ccfHPV-DNA	Plasma			Efficacy monitoring biomarker	^478^
	HOTAIR, PVT1, XLOC_000303, AL592284.1	Plasma	up		Early diagnostic biomarker	^482^
	miR-21, -25, -29a, -200a, -486-5p	Serum	up		Early diagnostic biomarker, Disease progression biomarker	^483^
	miR-196a	Serum	up		Disease progression biomarker, Prognostic biomarker	^484^
	miR-425-5p	Serum	up		Prognostic biomarker	^485^
	ESR1, ERBB2 mutation	Plasma	up		Efficacy monitoring biomarker	^486^
Endometrial cancer	CTCs	Peripheral blood	up		Early diagnostic biomarker	^498^
	CK-20	Peripheral blood	up		Tumor aggressiveness biomarker, Tumor recurrence biomarker	^550^
	CTNNB1, KRAS, PTEN, PIK3CA	Plasma	up		Tumor recurrence biomarker, Efficacy monitoring biomarker	^500^
	DNA methylation	Urine	up		Early diagnostic biomarker	^509^
Ovarian Cancer	claudin	Serum exosome	up		Early diagnostic biomarker	^523^
	miR-1307, miR-375	Serum exosome	up		Early diagnostic biomarker	^524^
Prostatic carcinoma	CTCs	Peripheral blood	up		Prognostic biomarker	^530^
	cfDNA mutation	Serum	up		Early diagnostic biomarker	^531^
	cfDNA	Plasma	up		Efficacy monitoring biomarker, Prognostic biomarker	^532^
	miR-21	Serum	up		Efficacy monitoring biomarker	^538^
	miR-141, miR-146b-3p, miR-194	Serum	up		Prognostic biomarker	^539^

## Cervical cancer (CC)

Cervical cancer (CC) is the fourth most common cancer among women worldwide, and HPV infection is the main cause of CC patients. Conventional screening and diagnostic tools are easily rejected because of their invasiveness, and as a non-invasive test, liquid biopsy may be an alternative and complementary tool to conventional screening and diagnosis. The following is a brief introduction to several common liquid biopsy methods.^477^

As an extremely important part of liquid biopsy, ctDNA is also clinically significant in patients with CC. Plasma ctDNA levels are significantly higher in patients with CC than in healthy controls and are strongly correlated with FIGO tumor stage, histologic grading, depth of infiltration, and lymphatic metastasis.^478^ Multiple studies have confirmed the potential use of this assay in clinical practice.^479^ And because of the high association between CC and human papillomavirus (HPV), it is also feasible to screen by detecting HPV cfDNA in the blood. In the detection of circulating HPV cfDNA, a magnetic bead-based HPV genotyping assay (E7-MPG) is an alternative method that is more accurate and significantly more sensitive (96.1%) than conventional dPCR.^480^ On the other hand, non-coding RNAs also have important roles in CC development and contribute to the early diagnosis of CC.^481^ Among them, lncRNAs and miRNAs have the most important roles. As a kind of lncRNA, HOX transcript antisense intragenic RNA (HOTAIR) is highly expressed in CC patients and promotes the proliferation and migration of tumor cells. Its combination with three other lncRNA (i.e., PVT1, AL592284.1, and XLOC_000303) significantly increased the positive predictive value (88%) and negative predictive value (84%) of CC.^482^ Meanwhile, combined testing of multiple miRNAs is often practiced in CC patients and can be more effective than individual testing. Jia et al. identified five serum miRNAs (i.e., miR-21, −25, −29a, −200a, and −486-5p) based on genome-wide miRNA sequencing and quantitative PCR (qPCR) validation, and the combination of these tests can differentiate CC patients from healthy controls.^483^

Moreover, studies have shown that genes such as miR-196a, miR-425-5p, and others have also been shown to be associated with the proliferation and migration of tumor cells in CC.^484,485^ In addition, another strategy for implementing liquid biopsy in CC detection involves the identification of somatic nucleotide variants (SNVs) in cancer driver genes.^486–488^ Tian et al. used an allelic fraction deviation (AFD) algorithm for evaluation and found that the value of AFD was positively correlated with the prognostic degree of patients.^489^ Initially, a phase III randomized clinical trial demonstrated that CTCs counts can be used as a predictive biomarker to guide the treatment of cervical cancer.^490^ Current strategies for the detection and isolation of CTCs in CC similarly rely on the physical and morphological characterization of the cells as well as on the identification and quantification of HPV oncogenes and epithelial markers through the use of molecular and/or immunofluorescence procedures.^491–493^ Compared to the detection of other solid tumors, there are fewer techniques related to the detection of CTCs in CC, and these methods have not yet been recognized.^494^

## Endometrial carcinoma (EC)

Endometrial carcinoma (EC) is the most common cancer of the female reproductive tract, and its incidence is increasing year by year,^495,496^ which seriously affects the quality of life for female patients. Presently, there are no diagnostic techniques available for detecting EC in the general population. Endometrial sample carries the risk of causing discomfort, bleeding, infection, and uterine perforation. Additionally, in up to 25% of cases, a biopsy may not provide enough information to make a diagnosis.^497^Therefore, as an alternative sampling method to traditional tissue biopsy, liquid biopsy has been a boon for early diagnosis in female patients due to its non-invasive nature. In the following, we provide a brief introduction to liquid biopsy methods mainly from the aspects of CTCs, ctDNA, miRNA, and extracellular vesicles.

First, CTCs were also found in patients with endometrial cancer and there was a high correlation between CTCs and EC.^498^ The detection rate of CTCs in the peripheral blood of patients with EC was 7–75%.^499^ This discrepancy may be related to the population characteristics, CTCs detection techniques, and the number of patients studied. Furthermore, ctDNA is clinically important for EC patients. It has been found that at least one ctDNA mutation can be detected in the peripheral blood of 94% EC patients, which mainly occurs in the CTNNB1, KRAS, PTEN, and PIK3CA genes.^500^ In a clinical trial, it was found that miRNA expression levels also differed between healthy adults and EC patients, such as miR-15b(AUC: 0.768), miR-27a(AUC: 0.813), and miR-223(AUC: 0.768) were differentially expressed between endometrial cancer patients and healthy individuals, which is important for improving the diagnosis of endometrial cancer.^501^Another study showed that CTCs were detected in patients with stage III and IV EC or in close proximity to the tumor, but not in patients with early-stage or recurrence, so the prognostic significance of CTCs for patients with EC is still controversial.^502^

And the number of ctDNAs found in the blood increases with the progression of the disease.^503^ Moreover, it has been demonstrated that arch-related mutations (DNMT3A and TET2 genes) may be associated with poor prognosis in endometrial cancer patients, and DNMT3A mutations are more likely to be detected in EC patients in particular.^504^ The simple method of extraction and the ability to store for a long time prior to analysis make the detection of ctDNAs expected to be a potential marker for the diagnosis of EC. Moreover, a variety of miRNAs have been associated with EC tumorigenesis, invasion, and metastasis. miR-183-5p, miR-429, and miR-146a-5p expression were found to be up-regulated by liquid biopsy examination of saline lavage fluid from patients with endometrial cancer after saline infusion ultrasound intrauterine scintigraphy (SIS) procedure, whereas miR-296-5p and miR-204-5p were decreased.^505^ In addition, studies of EVs have found that elevated levels of membrane-bound protein A2 in EVs correlate with high-risk histology, grading, staging, and recurrence risk of EC, suggesting that it may have a role to play in disease surveillance, recurrence, and early disease detection.^506^

In addition to the more mainstream liquid biopsy techniques described above, additional novel assays for the detection of liquid biopsies continue to be developed. For example, for the detection of messenger RNA molecules (mRNA) in plasma samples.^507^ However, relatively few studies have been conducted on this substance, probably due to its poor stability, easy degradation, and low levels of circulation, which makes it more difficult to detect and analyze.^508^ The detection of DNA methylation in urine may also provide an attractive noninvasive testing strategy for early screening of asymptomatic EC patients.^509^

## Ovarian cancer (OC)

Because there are no noticeable symptoms in the initial phases of the disease and no practical tests to detect it, almost 70% of patients with ovarian cancer (OC) are diagnosed in the advanced stages (Stages III and IV). Additionally, biomarkers like CA-125, which are specific to OC, are not sensitive or specific enough for regular screening.^510^ Thus, it is crucial to find new OC liquid biopsy markers.

The first is for classical CTCs, which are detected in OC patients by microbeads covered with the epithelial marker MOC31 or a mixture of cytokeratin and epidermal growth factor receptor (EGFR).^511,512^ The FDA approved the CellSearch® system (Menarini Silicon system, Italy) as the routine gold standard platform for CTCs isolation in clinical practice. Secondly, for ctDNA, the main methods currently used to identify ctDNA in the blood of OC patients are quantitative PCR, ddPCR, whole genome sequencing, and next-generation sequencing, which identify qualitative and quantitative alterations of ctDNA, such as gene fusions, aberrant DNA methylation, tumor-specific variants (TSV), copy number variants, and chromosomal instability.^513^ Up to now, most ctDNA identification techniques have focused on TP53 mutations in patients with high-grade plasmacytoid ovarian cancer (HGSOC).^514^ The use of ctDNA in OC reflects tumor heterogeneity more accurately than other assays and its shorter half-life makes it more precise compared to CA-125.^515,516^ However, the accuracy of ctDNA samples may be affected by its short half-life and low abundance in torrent blood (<0.5% of total cfDNA). Therefore, ctDNA analysis requires higher sensitivity techniques to minimize false negatives.^517^ On the other hand, in OC patients miRNAs are synthesized and activated faster than mRNAs and proteins, with a longer half-life.^518^ Therefore, miRNA may be more suitable for early OC detection.^519^ Several scholars have conducted studies on this topic, and in general, the current study found that lower overall survival in OC patients was mostly associated with the up-regulation of miR-21, miR-221, miR-141, and miR-429 and downregulation of miR-200c, miR-1290, miR-145, miR-199a, and miR-148a.^520^ In most of the studies, miRNA microarrays or NGS have been used to evaluate the miRNAs isolated from patients with ovarian cancer. In contrast to NGS, microarrays are more efficient and less cost-effective, but NGS has the potential to recognize novel miRNAs.^507^ In addition to miRNAs, lncRNAs and circRNAs have the potential to be diagnostic and prognostic liquid biopsy biomarkers of OC.^513^ CircRNAs are more stable in the peripheral circulation due to their specific covalent closure structure that makes them more resistant to destruction by RNase.^521^ It has been found that the expression of circRNAs in the differences between primary and metastatic sites of OC, may be associated with OC progression. As for lncRNAs, there are only partial data suggesting that clinical progression in OC patients is associated with lncRNA expression levels (XIST, H19, LSINCT5, AB073614, HOTAIR, CCAT2, and ANRIL). The diagnostic sensitivity and specificity of lncRNAs in these individuals have not yet been fully established, and no lncRNAs are licensed for therapeutic use, as their specific feasibility remains to be investigated.^507^ In addition, it was reported that the total exosome concentration was elevated in serum samples of OC patients, and exosomes of OC patients can carry a large number of miRNAs, therefore, exosomal proteins and miRNAs are the main indicators of exosomes and OC-related studies.^522^ Exosomal secreted proteins can be used as predictive or diagnostic indicators of ovarian cancer, e.g., it is seen in plasma and circulating exosomes of patients with OC overexpression of claudin-4 and can be used to monitor tumor progression.^523^ And the combined detection of exosomal miRNAs with the routine serum tumor biomarkers CA125 and HE4 can improve the detection rate of OC.^524^ In addition to this, thrombocytosis has been associated with increased cancer risk and shorter survival, especially in ovarian cancer. Because tumor cells are able to transcriptionally reprogram TEPs through multiple mechanisms,^525^ RNA sequencing of TEPs has become the latest component of liquid biopsy for tumor detection. It is also highly specific in the identification of OC and has been validated as a good test in different races and populations.

## Prostate cancer (PCa)

Prostate cancer (PCa) is the second leading cause of cancer-related deaths in men, but the survival rate of PC improves significantly after appropriate treatment, so early screening and diagnosis of this disease is of great importance.^526^ Currently, prostate-specific antigen (PSA) and transrectal ultrasound-guided biopsy are mostly used in clinical practice, but the former is not a specific marker for tumors with low sensitivity and specificity, while the latter often causes rejection due to its invasiveness.^527^ In contrast, liquid biopsy shows its superiority here, and we will introduce the relevant methods from the following aspects.

Firstly, for the detection of CTCs, in addition to using physical properties, it can also be isolated using its biological properties, such as antibody-antigen interactions.^528^ Detection of the number of CTCs in the blood by flow cytometry predicts the prognosis of metastatic desmoplasia-resistant prostate cancer (mCRPC). Briefly, it means that when the number of detected CTCs is higher, the tumor load is higher and the survival prognosis of the patient is worse. And high CTCs phenotypic heterogeneity was also associated with poorer survival outcomes in mCRPC. According to Prostate Cancer, Version 4.2023, NCCN Clinical Practice Guidelines in Oncology, AR-V7 expression on CTCs can help CRPC patients treated with abiraterone/enzalutamide make decisions about their next course of treatment.^529^ In terms of CTCs phenotype, Lindsay et al. demonstrated that Ki67 and vimentin expression in CTCs correlates with poor prognosis in mCRPC.^530^ Second, plasma ctDNA was found to be a potential clinical marker for the early detection of prostate cancer, and its concentration can come to differentiate between malignant disease and benign hyperplasia of the prostate. Ekkehard Schutz et al. analyzed ctDNA using whole-genome amplification and found differences in the number of ctDNA sequence reads in the 100 kbp interval between patients with PCa and the healthy population.^531^ Similar to CTCs, ctDNA can be used to assess the prognosis of cachectic-resistant prostate cancer (CRPC). Analysis of 663 plasma samples from 140 patients with CRPC showed that ctDNA was associated with poor survival prognosis.^532^ In addition to testing for specific ctDNAs, quantitative characterization of ctDNA can be used as a less invasive and more reliable prognostic biomarker, especially for DNA methylation. Hypermethylation of glutathione-s-transferase P1 (GSTP1) is the most common epigenetic alteration in PCa, and methylation-specific PCR (MSPCR) for this substance has high sensitivity and specificity to differentiate between normal and neoplastic states.^533^ On the other hand, in comparison to the coding genes described above, noncoding RNAs have a unique advantage due to their high tissue and staging specificity for the disease.^534^ miRNAs, in turn, have become one of the most widely studied small noncoding RNAs due to their remarkable stability in body fluids.^535–537^ Serum miR-21 has been reported to be a very useful biomarker. Moreover, it was found that serum miR-21 levels were positively correlated with serum PSA levels in patients with hormone-refractory prostate cancer (HRPC).^538^ Thus, it was concluded that MiR-21 has the potential to serve as a marker and predictor of hormone-refractory disease transformation. Detection of miRNAs in the serum of patients with and without tumor recurrence revealed statistically significant differences in the expression of miR-141, miR-146b-3p, and miR-194 between the recurrence and no recurrence groups (*P* < 0.05). It was hypothesized that the three had potential as biomarkers for predicting disease progression, as they were elevated in PCa patients who subsequently experienced relapse.^539^ In addition to this, the clinical significance of some lncRNAs in PCa patients has been gradually emphasized. Among them, a variety of lncRNAs, including PCAT1, PCGEM1, SChLAP1, and PCAT6, have expressed their advantages in different aspects of PCa and so on, which can provide some references for the diagnosis of clinical diseases.^540–543^ In addition, there are currently major limitations in the development of exosomes in cells due to technological development and associated cost issues. Logozzi et al. found that plasma levels of PSA-expressing EVs were higher in PCa patients than in healthy subjects.^544^ Del Re found that AR-V7 variants in the RNAs of EVs predicted response to ARSI.^545^ However, another study suggests that EVs may be less predictive than CTCs, which contain a higher amount of AR-V7.^546^ Overall, further experimental studies are needed for exosome detection in PCa patients. In addition to routine liquid biopsies, other tests such as protein biomarkers, e.g., PCA3, PSA glycosylation, and DNA methylation biomarkers, etc., are also included. Different tests have different preferences, but they offer great promise for personalized treatment strategies for PCa in the future.

## Seminoma

Seminoma is a common malignant tumor in men of reproductive age. However, routine diagnosis is a multi-step process with poor specificity. Liquid biopsy techniques can provide some early warnings to patients. Dora Raos et al. used pyrophosphate sequencing to assess liquid biopsy cfDNA methylation and compared it with samples from healthy volunteers. It was found that cfDNA methylation of OCT3/4, KITLG, and MAGEC2 can be used as potential non-invasive epigenetic biomarkers in liquid biopsies to some extent, but the conclusion still requires further experiments in larger populations. In addition to this, the results of the experiments will inevitably be affected because cancer-specific cfDNA methylation may be masked by cfDNA methylation in healthy cells.^547^

## Conclusion and perspective

Tissue biopsy remains the gold standard for tumor diagnosis due to its high level of laboratory standardization, good consistency of results, relatively stable samples, and high accuracy of results. However, there are some drawbacks to tissue biopsy, such as the fact that it is invasive, so the part with the highest risk of complications cannot be sampled, and it is difficult to repeat the sampling, making it unsuitable for regular testing and treatment evaluation. Additionally, the tumor information obtained is heavily influenced by the heterogeneity of the sample and can only reflect the information of the sampling site, among other things. As a result, exploring new screening modalities is beneficial to patient therapy and prognosis. Liquid biopsy is now undergoing rapid progress, however its application in clinical practice is still limited. Compared to standard examination methods, various advantages have been established, including little invasiveness, low risk, multiple repeat sampling, suitability for dynamic monitoring, and the ability to mitigate the effect of tumor heterogeneity to some extent. However, there are several limitations to liquid biopsy, such as a lack of laboratory standardization, which weakens the consistency of test findings from different laboratories, the high requirements for sample manipulation, the need to increase accuracy, and so on.^548^

Liquid biopsy holds significant potential for future applications, although it also presents several areas that require enhancement. One key limitation in the practical use of liquid biopsy is the need to isolate, purify, and detect the markers involved in the monitoring process. Hence, it is imperative to prioritize the advancement of novel detection technologies and analysis platforms, with the establishment of standardized operating procedures and unified data analysis, in order to enhance the accuracy of liquid biopsy in future development. Furthermore, it might be attempted to integrate with the swiftly advancing artificial intelligence, which has the potential to become a more effective way of detection. However, in terms of clinical application, it is important to note that a liquid biopsy can only provide information about specific molecules or biomarkers, and cannot fully capture the complex nature of a disease.^548^ Therefore, liquid biopsy cannot completely replace tissue biopsy. Instead, the two methods work together to offer a more comprehensive understanding of the biological aspects of tumors. In order to implement liquid biopsy in a widespread manner in clinical settings, it is necessary to conduct extensive clinical trials, standardize the processes for enriching the samples, and establish consistent methods for downstream analysis.

Hence, the enhancement of detection technology and the integration of liquid biopsy markers or the amalgamation of liquid biopsy with other detection methods could potentially facilitate the advancement and utilization of liquid biopsy technology. Ultimately, liquid biopsy has garnered significant attention and investigation, despite certain remaining research deficiencies. Nevertheless, it holds immense clinical utility.

## References

[1] Crosby, D. et al. Early detection of cancer. *Science* **375**, eaay9040 (2022). 35298272 10.1126/science.aay9040

[2] Vaidyanathan, R., Soon, R. H., Zhang, P., Jiang, K. & Lim, C. T. Cancer diagnosis: from tumor to liquid biopsy and beyond. *Lab. Chip* **19**, 11–34 (2018). 30480287 10.1039/c8lc00684a

[3] Li, W. et al. Liquid biopsy in lung cancer: significance in diagnostics, prediction, and treatment monitoring. *Mol. Cancer* **21**, 25 (2022). 35057806 10.1186/s12943-022-01505-z PMC8772097

[4] Casagrande, G. M. S., Silva, M. O., Reis, R. M. & Leal, L. F. Liquid biopsy for lung cancer: up-to-date and perspectives for screening programs. *Int. J. Mol. Sci.* **24**, 2505 (2023). 36768828 10.3390/ijms24032505 PMC9917347

[5] Pantel, K. & Alix-Panabières, C. Circulating tumour cells in cancer patients: challenges and perspectives. *Trends Mol. Med.* **16**, 398–406 (2010). 20667783 10.1016/j.molmed.2010.07.001

[6] Fu, Y., Zhang, Y. & Khoo, B. L. Liquid biopsy technologies for hematological diseases. *Med. Res. Rev.* **41**, 246–274 (2021). 32929726 10.1002/med.21731

[7] Lone, S. N. et al. Liquid biopsy: a step closer to transform diagnosis, prognosis and future of cancer treatments. *Mol. Cancer* **21**, 79 (2022). 35303879 10.1186/s12943-022-01543-7 PMC8932066

[8] Nikanjam, M., Kato, S. & Kurzrock, R. Liquid biopsy: current technology and clinical applications. *J. Hematol. Oncol.* **15**, 131 (2022). 36096847 10.1186/s13045-022-01351-y PMC9465933

[9] Galvis, M. M., Romero, C. S., Bueno, T. O. & Teng, Y. Toward a new era for the management of circulating tumor cells. *Adv. Exp. Med. Biol.* **1286**, 125–134 (2021). 33725350 10.1007/978-3-030-55035-6_9 PMC8647934

[10] Mandel, P. & Metais, P. Nuclear acids in human blood plasma. *C. R. Seances. Soc. Biol. Fil.* **142**, 241–243 (1948). 18875018

[11] Wolf, P. The nature and significance of platelet products in human plasma. *Br. J. Haematol.* **13**, 269–288 (1967). 6025241 10.1111/j.1365-2141.1967.tb08741.x

[12] Pan, B. T. & Johnstone, R. M. Fate of the transferrin receptor during maturation of sheep reticulocytes in vitro: selective externalization of the receptor. *Cell* **33**, 967–978 (1983). 6307529 10.1016/0092-8674(83)90040-5

[13] Leon, S. A., Shapiro, B., Sklaroff, D. M. & Yaros, M. J. Free DNA in the serum of cancer patients and the effect of therapy. *Cancer Res.* **37**, 646–650 (1977). 837366

[14] Alix-Panabières, C. & Pantel, K. Liquid biopsy: from discovery to clinical application. *Cancer Discov.* **11**, 858–873 (2021). 33811121 10.1158/2159-8290.CD-20-1311

[15] Sorenson, G. D. et al. Soluble normal and mutated DNA sequences from single-copy genes in human blood. *Cancer Epidemiol. Biomark. Prev.* **3**, 67–71 (1994). 8118388

[16] Raposo, G. et al. B lymphocytes secrete antigen-presenting vesicles. *J. Exp. Med.* **183**, 1161–1172 (1996). 8642258 10.1084/jem.183.3.1161 PMC2192324

[17] Smirnov, D. A. et al. Global gene expression profiling of circulating tumor cells. *Cancer Res.* **65**, 4993–4997 (2005). 15958538 10.1158/0008-5472.CAN-04-4330

[18] Diehl, F. et al. Circulating mutant DNA to assess tumor dynamics. *Nat. Med* **14**, 985–990 (2008). 18670422 10.1038/nm.1789 PMC2820391

[19] Wu, J., Lu, A. D., Zhang, L. P., Zuo, Y. X. & Jia, Y. P. Study of clinical outcome and prognosis in pediatric core binding factor-acute myeloid leukemia. *Zhonghua. Xue. Ye. Xue. Za. Zhi.* **40**, 52–57 (2019). 30704229 10.3760/cma.j.issn.0253-2727.2019.01.010 PMC7351698

[20] Giuliano, A. E., Edge, S. B. & Hortobagyi, G. N. Eighth edition of the AJCC cancer staging manual: breast cancer. *Ann. Surg. Oncol.* **25**, 1783–1785 (2018). 29671136 10.1245/s10434-018-6486-6

[21] Huang, X. & Yin, Y. M. [Updates of Chinese society of clinical oncology (CSCO) guideline for breast cancer in 2018]. *Zhonghua. Yi. Xue. Za. Zhi.* **98**, 1213–1217 (2018). 29747306 10.3760/cma.j.issn.0376-2491.2018.16.005

[22] Yu, M., Stott, S., Toner, M., Maheswaran, S. & Haber, D. A. Circulating tumor cells: approaches to isolation and characterization. *J. Cell Biol.* **192**, 373–382 (2011). 21300848 10.1083/jcb.201010021 PMC3101098

[23] Lianidou, E. S., Strati, A. & Markou, A. Circulating tumor cells as promising novel biomarkers in solid cancers. *Crit. Rev. Clin. Lab Sci.* **51**, 160–171 (2014). 24641350 10.3109/10408363.2014.896316

[24] Agashe, R. & Kurzrock, R. Circulating tumor cells: from the laboratory to the cancer clinic. *Cancers* **12**, 3065 (2020). 32825548 10.3390/cancers12092361 PMC7564158

[25] Salu, P. & Reindl, K. M. Advancements in circulating tumor cell research: bridging biology and clinical applications. *Cancers* **16**, 1213 (2024). 38539545 10.3390/cancers16061213 PMC10969710

[26] Cohen, S. J. et al. Relationship of circulating tumor cells to tumor response, progression-free survival, and overall survival in patients with metastatic colorectal cancer. *J. Clin. Oncol.* **26**, 3213–3221 (2008). 18591556 10.1200/JCO.2007.15.8923

[27] Marcuello, M. et al. Circulating biomarkers for early detection and clinical management of colorectal cancer. *Mol. Asp. Med.* **69**, 107–122 (2019). 10.1016/j.mam.2019.06.002 31189073

[28] Qiu, J. et al. Refining cancer management using integrated liquid biopsy. *Theranostics* **10**, 2374–2384 (2020). 32089746 10.7150/thno.40677 PMC7019147

[29] Sefrioui, D. et al. Diagnostic value of CA19.9, circulating tumour DNA and circulating tumour cells in patients with solid pancreatic tumours. *Br. J. Cancer* **117**, 1017–1025 (2017). 28772284 10.1038/bjc.2017.250 PMC5625666

[30] Cristofanilli, M. et al. Circulating tumor cells, disease progression, and survival in metastatic breast cancer. *N. Engl. J. Med.* **351**, 781–791 (2004). 15317891 10.1056/NEJMoa040766

[31] Vidlarova, M. et al. Recent advances in methods for circulating tumor cell detection. *Int. J. Mol. Sci.* **24**, 3902 (2023). 36835311 10.3390/ijms24043902 PMC9959336

[32] Russo, G. I. et al. The role of dielectrophoresis for cancer diagnosis and prognosis. *Cancers* **14**, 198 (2021). 35008359 10.3390/cancers14010198 PMC8750463

[33] Lozar, T. et al. Preclinical and clinical evaluation of magnetic-activated cell separation technology for CTC isolation in breast cancer. *Front Oncol.* **10**, 554554 (2020). 33042837 10.3389/fonc.2020.554554 PMC7522616

[34] Petrik, J. et al. Circulating tumor cells in colorectal cancer: detection systems and clinical utility. *Int. J. Mol. Sci.* **23**, 13582 (2022). 36362369 10.3390/ijms232113582 PMC9654465

[35] Li, Y. Z., Kong, S. N., Liu, Y. P., Yang, Y. & Zhang, H. M. Can liquid biopsy based on ctDNA/cfDNA replace tissue biopsy for the precision treatment of EGFR-mutated NSCLC? *J. Clin. Med.* **12**, 1438 (2023). 36835972 10.3390/jcm12041438 PMC9966257

[36] Campos-Carrillo, A. et al. Circulating tumor DNA as an early cancer detection tool. *Pharm. Ther.* **207**, 107458 (2020). 10.1016/j.pharmthera.2019.107458 PMC6957244 31863816

[37] Underhill, H. R. et al. Fragment length of circulating tumor DNA. *PLoS Genet* **12**, e1006162 (2016). 27428049 10.1371/journal.pgen.1006162 PMC4948782

[38] Chaudhuri, A. A. et al. Early detection of molecular residual disease in localized lung cancer by circulating tumor DNA profiling. *Cancer Discov.* **7**, 1394–1403 (2017). 28899864 10.1158/2159-8290.CD-17-0716 PMC5895851

[39] Jia, N. et al. Association of emergence of new mutations in circulating tumuor DNA during chemotherapy with clinical outcome in metastatic colorectal cancer. *BMC Cancer* **21**, 845 (2021). 34294055 10.1186/s12885-021-08309-2 PMC8296534

[40] Chen, Z. et al. Liquid biopsies for cancer: from bench to clinic. *MedComm* **4**, e329 (2023). 37492785 10.1002/mco2.329 PMC10363811

[41] Hannigan, B. et al. Liquid biopsy assay for lung carcinoma using centrifuged supernatants from fine-needle aspiration specimens. *Ann. Oncol.* **30**, 963–969 (2019). 30887015 10.1093/annonc/mdz102

[42] Olmedillas-López, S., Olivera-Salazar, R., García-Arranz, M. & García-Olmo, D. Current and emerging applications of droplet digital PCR in oncology: an updated review. *Mol. Diagn. Ther.* **26**, 61–87 (2022). 34773243 10.1007/s40291-021-00562-2

[43] Ståhlberg, A. et al. Simple multiplexed PCR-based barcoding of DNA for ultrasensitive mutation detection by next-generation sequencing. *Nat. Protoc.* **12**, 664–682 (2017). 28253235 10.1038/nprot.2017.006

[44] Johnstone, R. M., Adam, M., Hammond, J. R., Orr, L. & Turbide, C. Vesicle formation during reticulocyte maturation. Association of plasma membrane activities with released vesicles (exosomes). *J. Biol. Chem.* **262**, 9412–9420 (1987). 3597417

[45] Witwer, K. W. & Théry, C. Extracellular vesicles or exosomes? On primacy, precision, and popularity influencing a choice of nomenclature. *J. Extracell. Vesicles* **8**, 1648167 (2019). 31489144 10.1080/20013078.2019.1648167 PMC6711079

[46] van Niel, G. et al. Challenges and directions in studying cell-cell communication by extracellular vesicles. *Nat. Rev. Mol. Cell Biol.* **23**, 369–382 (2022). 35260831 10.1038/s41580-022-00460-3

[47] Zhou, Y., Zhang, Y., Gong, H., Luo, S. & Cui, Y. The role of exosomes and their applications in cancer. *Int. J. Mol. Sci.* **22**, 12204 (2021). 34830085 10.3390/ijms222212204 PMC8622108

[48] Han, Q. F. et al. Exosome biogenesis: machinery, regulation, and therapeutic implications in cancer. *Mol. Cancer* **21**, 207 (2022). 36320056 10.1186/s12943-022-01671-0 PMC9623991

[49] Wang, J. et al. Exosomal microRNAs as liquid biopsy biomarkers in prostate cancer. *Crit. Rev. Oncol. Hematol.* **145**, 102860 (2020). 31874447 10.1016/j.critrevonc.2019.102860

[50] Wang, J., Yang, K., Yuan, W. & Gao, Z. Determination of serum exosomal H19 as a noninvasive biomarker for bladder cancer diagnosis and prognosis. *Med. Sci. Monit.* **24**, 9307–9316 (2018). 30576305 10.12659/MSM.912018 PMC6320644

[51] Keerthikumar, S. et al. ExoCarta: a web-based compendium of exosomal cargo. *J. Mol. Biol.* **428**, 688–692 (2016). 26434508 10.1016/j.jmb.2015.09.019 PMC4783248

[52] Wu, X. et al. Extracellular vesicle packaged LMP1-activated fibroblasts promote tumor progression via autophagy and stroma-tumor metabolism coupling. *Cancer Lett.* **478**, 93–106 (2020). 32160975 10.1016/j.canlet.2020.03.004

[53] Kalluri, R. & LeBleu, V. S. The biology, function, and biomedical applications of exosomes. *Science* **367**, eaau6977 (2020). 32029601 10.1126/science.aau6977 PMC7717626

[54] Asare-Werehene, M. et al. The exosome-mediated autocrine and paracrine actions of plasma gelsolin in ovarian cancer chemoresistance. *Oncogene* **39**, 1600–1616 (2020). 31700155 10.1038/s41388-019-1087-9 PMC7018662

[55] Mu, Y. et al. Study of serum exosome miRNA as a biomarker for early onset adult ouclar myastthenia gravis. *Gene* **896**, 148034 (2024). 38013129 10.1016/j.gene.2023.148034

[56] Chen, Y. et al. Exosomal derived miR-1246 from hydroquinone-transformed cells drives S phase accumulation arrest by targeting cyclin G2 in TK6 cells. *Chem. Biol. Interact.* **387**, 110809 (2024). 38006958 10.1016/j.cbi.2023.110809

[57] Chen, J. et al. Review on strategies and technologies for exosome isolation and purification. *Front Bioeng. Biotechnol.* **9**, 811971 (2021). 35071216 10.3389/fbioe.2021.811971 PMC8766409

[58] Franco, A. T., Corken, A. & Ware, J. Platelets at the interface of thrombosis, inflammation, and cancer. *Blood* **126**, 582–588 (2015). 26109205 10.1182/blood-2014-08-531582 PMC4520875

[59] Italiano, J. E. Jr. & Battinelli, E. M. Selective sorting of alpha-granule proteins. *J. Thromb. Haemost.* **7**, 173–176 (2009). 19630794 10.1111/j.1538-7836.2009.03387.x PMC3004150

[60] Tranum, B. L. & Haut, A. Thrombocytosis: platelet kinetics in neoplasia. *J. Lab. Clin. Med.* **84**, 615–619 (1974). 4283783

[61] Bailey, S. E., Ukoumunne, O. C., Shephard, E. A. & Hamilton, W. Clinical relevance of thrombocytosis in primary care: a prospective cohort study of cancer incidence using English electronic medical records and cancer registry data. *Br. J. Gen. Pr.* **67**, e405–e413 (2017). 10.3399/bjgp17X691109 PMC5442956 28533199

[62] Qi, C. et al. P-selectin-mediated adhesion between platelets and tumor cells promotes intestinal tumorigenesis in Apc(Min/+) mice. *Int. J. Biol. Sci.* **11**, 679–687 (2015). 25999791 10.7150/ijbs.11589 PMC4440258

[63] Wang, Y. et al. Application of tumor-educated platelets as new fluid biopsy markers in various tumors. *Clin. Transl. Oncol.* **25**, 114–125 (2023). 36284061 10.1007/s12094-022-02937-1

[64] Best, M. G., Wesseling, P. & Wurdinger, T. Tumor-educated platelets as a noninvasive biomarker source for cancer detection and progression monitoring. *Cancer Res.* **78**, 3407–3412 (2018). 29921699 10.1158/0008-5472.CAN-18-0887

[65] Nilsson, R. J. et al. Blood platelets contain tumor-derived RNA biomarkers. *Blood* **118**, 3680–3683 (2011). 21832279 10.1182/blood-2011-03-344408 PMC7224637

[66] Stratz, C. et al. Micro-array profiling exhibits remarkable intra-individual stability of human platelet micro-RNA. *Thromb. Haemost.* **107**, 634–641 (2012). 22371016 10.1160/TH11-10-0742

[67] Best, M. G. et al. RNA-seq of tumor-educated platelets enables blood-based pan-cancer, multiclass, and molecular pathway cancer diagnostics. *Cancer Cell* **28**, 666–676 (2015). 26525104 10.1016/j.ccell.2015.09.018 PMC4644263

[68] Ye, B. et al. A panel of platelet-associated circulating long non-coding RNAs as potential biomarkers for colorectal cancer. *Genomics* **114**, 31–37 (2022). 34843904 10.1016/j.ygeno.2021.11.026

[69] Zou, D. et al. PltDB: a blood platelets-based gene expression database for disease investigation. *Bioinformatics* **38**, 3143–3145 (2022). 35438150 10.1093/bioinformatics/btac278

[70] Zhang, M., Dang, P., Liu, Y., Qiao, B. & Sun, Z. Noncoding RNAs in pyroptosis and cancer progression: Effect, mechanism, and clinical application. *Front Immunol.* **13**, 982040 (2022). 36059539 10.3389/fimmu.2022.982040 PMC9428448

[71] Toden, S. & Goel, A. Non-coding RNAs as liquid biopsy biomarkers in cancer. *Br. J. Cancer* **126**, 351–360 (2022). 35013579 10.1038/s41416-021-01672-8 PMC8810986

[72] Saliminejad, K., Khorram Khorshid, H. R., Soleymani Fard, S. & Ghaffari, S. H. An overview of microRNAs: Biology, functions, therapeutics, and analysis methods. *J. Cell Physiol.* **234**, 5451–5465 (2019). 30471116 10.1002/jcp.27486

[73] Tili, E., Croce, C. M. & Michaille, J. J. miR-155: on the crosstalk between inflammation and cancer. *Int Rev. Immunol.* **28**, 264–284 (2009). 19811312 10.1080/08830180903093796

[74] Boon, R. A., Jaé, N., Holdt, L. & Dimmeler, S. Long noncoding RNAs: from clinical genetics to therapeutic targets? *J. Am. Coll. Cardiol.* **67**, 1214–1226 (2016). 26965544 10.1016/j.jacc.2015.12.051

[75] Bridges, M. C., Daulagala, A. C. & Kourtidis, A. LNCcation: lncRNA localization and function. *J. Cell Biol.* **220**, e202009045 (2021). 33464299 10.1083/jcb.202009045 PMC7816648

[76] Schmitt, A. M. & Chang, H. Y. Long noncoding RNAs in cancer pathways. *Cancer Cell* **29**, 452–463 (2016). 27070700 10.1016/j.ccell.2016.03.010 PMC4831138

[77] Ramón, Y. C. S., Segura, M. F. & Hümmer, S. Interplay between ncRNAs and cellular communication: a proposal for understanding cell-specific signaling pathways. *Front Genet.* **10**, 281 (2019). 31001323 10.3389/fgene.2019.00281 PMC6454836

[78] Chen, Q. et al. Plasma long non-coding RNA MALAT1 is associated with distant metastasis in patients with epithelial ovarian cancer. *Oncol. Lett.* **12**, 1361–1366 (2016). 27446438 10.3892/ol.2016.4800 PMC4950178

[79] Lin, Q. et al. MALAT1 affects ovarian cancer cell behavior and patient survival. *Oncol. Rep.* **39**, 2644–2652 (2018). 29693187 10.3892/or.2018.6384 PMC5983936

[80] Takahashi, K. et al. Long non-coding RNAs in epithelial-mesenchymal transition of pancreatic cancer. *Front Mol. Biosci.* **8**, 717890 (2021). 34820419 10.3389/fmolb.2021.717890 PMC8606592

[81] Hu, D. et al. Peripheral blood-based DNA methylation of long non-coding RNA H19 and metastasis-associated lung adenocarcinoma transcript 1 promoters are potential non-invasive biomarkers for gastric cancer detection. *Cancer Control* **28**, 10732748211043667 (2021). 34615385 10.1177/10732748211043667 PMC8504648

[82] Feng, Z. H. et al. m6A-immune-related lncRNA prognostic signature for predicting immune landscape and prognosis of bladder cancer. *J. Transl. Med.* **20**, 492 (2022). 36309694 10.1186/s12967-022-03711-1 PMC9617388

[83] Jin, T. LncRNA DRAIR is a novel prognostic and diagnostic biomarker for gastric cancer. *Mamm. Genome.* **32**, 503–507 (2021). 34510245 10.1007/s00335-021-09911-2

[84] Zhang, G., Sun, J. & Zhang, X. A novel Cuproptosis-related LncRNA signature to predict prognosis in hepatocellular carcinoma. *Sci. Rep.* **12**, 11325 (2022). 35790864 10.1038/s41598-022-15251-1 PMC9256635

[85] Diener, T. O. Potato spindle tuber “virus”. IV. A replicating, low molecular weight RNA. *Virology* **45**, 411–428 (1971). 5095900 10.1016/0042-6822(71)90342-4

[86] Sanger, H. L., Klotz, G., Riesner, D., Gross, H. J. & Kleinschmidt, A. K. Viroids are single-stranded covalently closed circular RNA molecules existing as highly base-paired rod-like structures. *Proc. Natl. Acad. Sci. USA* **73**, 3852–3856 (1976). 1069269 10.1073/pnas.73.11.3852 PMC431239

[87] Hentze, M. W. & Preiss, T. Circular RNAs: splicing’s enigma variations. *EMBO J.* **32**, 923–925 (2013). 23463100 10.1038/emboj.2013.53 PMC3616293

[88] Peng, D. et al. CircRNA: an emerging star in the progression of glioma. *Biomed. Pharmacother.* **151**, 113150 (2022). 35623170 10.1016/j.biopha.2022.113150

[89] Liang, Y. et al. A brief review of circRNA biogenesis, detection, and function. *Curr. Genomics* **22**, 485–495 (2021). 35386433 10.2174/1389202922666210331130722 PMC8905635

[90] Zhang, Y., Liu, Q. & Liao, Q. CircHIPK3: a promising cancer-related circular RNA. *Am. J. Transl. Res.* **12**, 6694–6704 (2020). 33194066 PMC7653572

[91] Liu, T., Huang, T., Shang, M. & Han, G. CircRNA ITCH: insight into its role and clinical application prospect in tumor and non-tumor diseases. *Front. Genet.* **13**, 927541 (2022). 35910224 10.3389/fgene.2022.927541 PMC9335290

[92] Su, K., Yi, Q., Dai, X. & Liu, O. Circular RNA ITCH: an emerging multifunctional regulator. *Biomolecules* **12**, 359 (2022). 35327551 10.3390/biom12030359 PMC8944968

[93] Kojima, T. et al. A simple biological imaging system for detecting viable human circulating tumor cells. *J. Clin. Investig.* **119**, 3172–3181 (2009). 19729837 10.1172/JCI38609 PMC2752068

[94] Gao, F. et al. Circulating tumor cell is a common property of brain glioma and promotes the monitoring system. *Oncotarget* **7**, 71330–71340 (2016). 27517490 10.18632/oncotarget.11114 PMC5342081

[95] Hu, B. et al. Comprehensive atlas of circulating rare cells detected by SE-iFISH and image scanning platform in patients with various diseases. *Front. Oncol.* **12**, 821454 (2022). 35311070 10.3389/fonc.2022.821454 PMC8924462

[96] Rushton, A. J., Nteliopoulos, G., Shaw, J. A. & Coombes, R. C. A review of circulating tumour cell enrichment technologies. *Cancers* **13**, 970 (2021). 33652649 10.3390/cancers13050970 PMC7956528

[97] Andree, K. C., van Dalum, G. & Terstappen, L. W. Challenges in circulating tumor cell detection by the CellSearch system. *Mol. Oncol.* **10**, 395–407 (2016). 26795350 10.1016/j.molonc.2015.12.002 PMC5528971

[98] Kojabad, A. A. et al. Droplet digital PCR of viral DNA/RNA, current progress, challenges, and future perspectives. *J. Med. Virol.* **93**, 4182–4197 (2021). 33538349 10.1002/jmv.26846 PMC8013307

[99] Lin, C., Liu, X., Zheng, B., Ke, R. & Tzeng, C. M. Liquid biopsy, ctDNA diagnosis through NGS. *Life* **11**, 890 (2021). 34575039 10.3390/life11090890 PMC8468354

[100] Mandlik, J. S., Patil, A. S. & Singh, S. Next-generation sequencing (NGS): platforms and applications. *J. Pharm. Bioallied Sci.* **16**, S41–s45 (2024). 38595613 10.4103/jpbs.jpbs_838_23 PMC11001031

[101] Sidhom, K., Obi, P. O. & Saleem, A. A review of exosomal isolation methods: is size exclusion chromatography the best option? *Int. J. Mol. Sci.* **21**, 6466 (2020). 32899828 10.3390/ijms21186466 PMC7556044

[102] Meyer, C., Garzia, A. & Tuschl, T. Simultaneous detection of the subcellular localization of RNAs and proteins in cultured cells by combined multicolor RNA-FISH and IF. *Methods* **118-119**, 101–110 (2017). 27664292 10.1016/j.ymeth.2016.09.010 PMC5360554

[103] Freeman, W. M., Walker, S. J. & Vrana, K. E. Quantitative RT-PCR: pitfalls and potential. *Biotechniques* **26**, 112–122 (1999). 124-115. 9894600 10.2144/99261rv01

[104] He, S. L. & Green, R. Northern blotting. *Methods Enzymol.* **530**, 75–87 (2013). 24034315 10.1016/B978-0-12-420037-1.00003-8 PMC4287216

[105] Urbanek, M. O., Nawrocka, A. U. & Krzyzosiak, W. J. Small RNA detection by in situ hybridization methods. *Int. J. Mol. Sci.* **16**, 13259–13286 (2015). 26068454 10.3390/ijms160613259 PMC4490494

[106] Hong, M. et al. RNA sequencing: new technologies and applications in cancer research. *J. Hematol. Oncol.* **13**, 166 (2020). 33276803 10.1186/s13045-020-01005-x PMC7716291

[107] Zhou, Z. et al. Liquid biopsy in hepatocellular carcinoma. *Methods Mol. Biol.* **2695**, 213–225 (2023). 37450121 10.1007/978-1-0716-3346-5_14

[108] Ghosh, S. et al. The exosome encapsulated microRNAs as circulating diagnostic marker for hepatocellular carcinoma with low alpha-fetoprotein. *Int. J. Cancer* **147**, 2934–2947 (2020). 32441313 10.1002/ijc.33111

[109] Jiang, S. S. et al. Galectin-3 is associated with a poor prognosis in primary hepatocellular carcinoma. *J. Transl. Med.* **12**, 273 (2014). 25260879 10.1186/s12967-014-0273-3 PMC4179848

[110] Arbelaiz, A. et al. Serum extracellular vesicles contain protein biomarkers for primary sclerosing cholangitis and cholangiocarcinoma. *Hepatology* **66**, 1125–1143 (2017). 28555885 10.1002/hep.29291

[111] von Felden, J. et al. Mutations in circulating tumor DNA predict primary resistance to systemic therapies in advanced hepatocellular carcinoma. *Oncogene* **40**, 140–151 (2021). 33097857 10.1038/s41388-020-01519-1 PMC12452111

[112] Kisiel, J. B. et al. Hepatocellular carcinoma detection by plasma methylated DNA: discovery, phase I pilot, and phase II clinical validation. *Hepatology* **69**, 1180–1192 (2019). 30168613 10.1002/hep.30244 PMC6429916

[113] Prospective suRveillance for very Early hepatoCellular cARcinoma(PreCar) expert panel. [Expert consensus on early screening strategies for liver cancer in China]. *Zhonghua. Gan. Zang. Bing. Za. Zhi.* **29**, 515–522 (2021). 10.3760/cma.j.cn501113-20210605-00264 PMC12814720 34192841

[114] Qi, L. N. et al. Circulating tumor cells undergoing EMT provide a metric for diagnosis and prognosis of patients with hepatocellular carcinoma. *Cancer Res.* **78**, 4731–4744 (2018). 29915159 10.1158/0008-5472.CAN-17-2459

[115] Park, S., Lee, E. J., Rim, C. H. & Seong, J. Plasma cell-free DNA as a predictive marker after radiotherapy for hepatocellular carcinoma. *Yonsei Med. J.* **59**, 470–479 (2018). 29749129 10.3349/ymj.2018.59.4.470 PMC5949288

[116] Chen, J., Cao, S. W., Cai, Z., Zheng, L. & Wang, Q. Epithelial-mesenchymal transition phenotypes of circulating tumor cells correlate with the clinical stages and cancer metastasis in hepatocellular carcinoma patients. *Cancer Biomark.* **20**, 487–498 (2017). 28869439 10.3233/CBM-170315

[117] Bai, T. et al. Circulating tumor cells and CXCR4 in the prognosis of hepatocellular carcinoma. *Transl. Cancer Res.* **9**, 1384–1394 (2020). 35117486 10.21037/tcr.2020.01.14 PMC8798757

[118] Izquierdo-Sanchez, L. et al. Cholangiocarcinoma landscape in Europe: diagnostic, prognostic and therapeutic insights from the ENSCCA Registry. *J. Hepatol.* **76**, 1109–1121 (2022). 35167909 10.1016/j.jhep.2021.12.010

[119] Valle, J. W. et al. Biliary cancer: ESMO clinical practice guidelines for diagnosis, treatment and follow-up. *Ann. Oncol.* **27**, v28–v37 (2016). 27664259 10.1093/annonc/mdw324

[120] Wang, Y. et al. Diagnostic and prognostic value of circulating miR-21 for cancer: a systematic review and meta-analysis. *Gene* **533**, 389–397 (2014). 24076132 10.1016/j.gene.2013.09.038

[121] Yan, Q. et al. The serum MicroRNA signatures for pancreatic cancer detection and operability evaluation. *Front. Bioeng. Biotechnol.* **8**, 379 (2020). 32411694 10.3389/fbioe.2020.00379 PMC7201024

[122] Correa-Gallego, C. et al. Circulating plasma levels of MicroRNA-21 and MicroRNA-221 are potential diagnostic markers for primary intrahepatic cholangiocarcinoma. *PLoS ONE* **11**, e0163699 (2016). 27685844 10.1371/journal.pone.0163699 PMC5042503

[123] Leelawat, K. et al. Prognostic relevance of circulating CK19 mRNA in advanced malignant biliary tract diseases. *World J. Gastroenterol.* **18**, 175–181 (2012). 22253524 10.3748/wjg.v18.i2.175 PMC3257445

[124] Zhou, K. Q. et al. Circulating osteopontin per tumor volume as a prognostic biomarker for resectable intrahepatic cholangiocarcinoma. *Hepatobiliary Surg. Nutr.* **8**, 582–596 (2019). 31929985 10.21037/hbsn.2019.03.14 PMC6943027

[125] Julich-Haertel, H. et al. Cancer-associated circulating large extracellular vesicles in cholangiocarcinoma and hepatocellular carcinoma. *J. Hepatol.* **67**, 282–292 (2017). 28267620 10.1016/j.jhep.2017.02.024

[126] Xu, H. et al. Elevation of serum KL-6 mucin levels in patients with cholangiocarcinoma. *Hepatogastroenterology* **55**, 2000–2004 (2008). 19260467

[127] Leelawat, K., Sakchinabut, S., Narong, S. & Wannaprasert, J. Detection of serum MMP-7 and MMP-9 in cholangiocarcinoma patients: evaluation of diagnostic accuracy. *BMC Gastroenterol.* **9**, 30 (2009). 19405942 10.1186/1471-230X-9-30 PMC2680894

[128] Kobayashi, S., Werneburg, N. W., Bronk, S. F., Kaufmann, S. H. & Gores, G. J. Interleukin-6 contributes to Mcl-1 up-regulation and TRAIL resistance via an Akt-signaling pathway in cholangiocarcinoma cells. *Gastroenterology* **128**, 2054–2065 (2005). 15940637 10.1053/j.gastro.2005.03.010

[129] Huang, L. et al. Serum CYFRA 21-1 in biliary tract cancers: a reliable biomarker for gallbladder carcinoma and intrahepatic cholangiocarcinoma. *Dig. Dis. Sci.* **60**, 1273–1283 (2015). 25487191 10.1007/s10620-014-3472-0

[130] Loosen, S. H. et al. Elevated levels of circulating osteopontin are associated with a poor survival after resection of cholangiocarcinoma. *J. Hepatol.* **67**, 749–757 (2017). 28668580 10.1016/j.jhep.2017.06.020

[131] Yang, J. D. et al. Circulating tumor cells are associated with poor overall survival in patients with cholangiocarcinoma. *Hepatology* **63**, 148–158 (2016). 26096702 10.1002/hep.27944 PMC4684812

[132] Shen, N. et al. Bile cell-free DNA as a novel and powerful liquid biopsy for detecting somatic variants in biliary tract cancer. *Oncol. Rep.* **42**, 549–560 (2019). 31173267 10.3892/or.2019.7177 PMC6610033

[133] Goyal, L. et al. Polyclonal secondary FGFR2 mutations drive acquired resistance to FGFR inhibition in patients with FGFR2 fusion-positive cholangiocarcinoma. *Cancer Discov.* **7**, 252–263 (2017). 28034880 10.1158/2159-8290.CD-16-1000 PMC5433349

[134] Zygulska, A. L. & Pierzchalski, P. Novel diagnostic biomarkers in colorectal cancer. *Int. J. Mol. Sci.* **23**, 852 (2022). 35055034 10.3390/ijms23020852 PMC8776048

[135] Takano, Y. et al. Circulating exosomal microRNA-203 is associated with metastasis possibly via inducing tumor-associated macrophages in colorectal cancer. *Oncotarget* **8**, 78598–78613 (2017). 29108252 10.18632/oncotarget.20009 PMC5667985

[136] Teng, Y. et al. MVP-mediated exosomal sorting of miR-193a promotes colon cancer progression. *Nat. Commun.* **8**, 14448 (2017). 28211508 10.1038/ncomms14448 PMC5321731

[137] Tsukamoto, M., Iinuma, H., Yagi, T., Matsuda, K. & Hashiguchi, Y. Circulating exosomal MicroRNA-21 as a biomarker in each tumor stage of colorectal cancer. *Oncology* **92**, 360–370 (2017). 28376502 10.1159/000463387

[138] Fu, F., Jiang, W., Zhou, L. & Chen, Z. Circulating exosomal miR-17-5p and miR-92a-3p predict pathologic stage and grade of colorectal cancer. *Transl. Oncol.* **11**, 221–232 (2018). 29367070 10.1016/j.tranon.2017.12.012 PMC5789766

[139] Zeng, Z. et al. Cancer-derived exosomal miR-25-3p promotes pre-metastatic niche formation by inducing vascular permeability and angiogenesis. *Nat. Commun.* **9**, 5395 (2018). 30568162 10.1038/s41467-018-07810-w PMC6300604

[140] Karimi, N. et al. Serum overexpression of miR-301a and miR-23a in patients with colorectal cancer. *J. Chin. Med Assoc.* **82**, 215–220 (2019). 30913118 10.1097/JCMA.0000000000000031

[141] Concepcion, C. P., Bonetti, C. & Ventura, A. The microRNA-17-92 family of microRNA clusters in development and disease. *Cancer J.* **18**, 262–267 (2012). 22647363 10.1097/PPO.0b013e318258b60a PMC3592780

[142] Matsumura, T. et al. Exosomal microRNA in serum is a novel biomarker of recurrence in human colorectal cancer. *Br. J. Cancer* **113**, 275–281 (2015). 26057451 10.1038/bjc.2015.201 PMC4506387

[143] Yang, C., Zhuang, W., Hu, Y. & Zhu, L. Clinical significance of peripheral circulating tumor cell counts in colorectal polyps and non-metastatic colorectal cancer. *World J. Surg. Oncol.* **16**, 13 (2018). 29357895 10.1186/s12957-017-1305-2 PMC5778777

[144] Tsai, W. S. et al. Novel circulating tumor cell assay for detection of colorectal adenomas and cancer. *Clin. Transl. Gastroenterol.* **10**, e00088 (2019). 31663904 10.14309/ctg.0000000000000088 PMC6884354

[145] Ganig, N. et al. Proteomic analyses of fibroblast- and serum-derived exosomes identify QSOX1 as a marker for non-invasive detection of colorectal cancer. *Cancers* **13**, 1351 (2021). 33802764 10.3390/cancers13061351 PMC8002505

[146] Wang, F., Zhang, Y., Chen, D., Zhang, Z. & Li, Z. Single microbead-based fluorescent aptasensor (SMFA) for direct isolation and in situ quantification of exosomes from plasma. *Analyst* **146**, 3346–3351 (2021). 33999063 10.1039/d1an00463h

[147] Ren, D. et al. Maintenance of cancer stemness by miR-196b-5p contributes to chemoresistance of colorectal cancer cells via activating STAT3 signaling pathway. *Oncotarget* **8**, 49807–49823 (2017). 28591704 10.18632/oncotarget.17971 PMC5564809

[148] Camera, S. et al. Prognostic value of the pace of tumor progression as assessed by serial (18)F-FDG PET/CT scan and liquid biopsy in refractory colorectal cancer: the Coriolan trial. *Cancers* **12**, 2752 (2020). 32987838 10.3390/cancers12102752 PMC7601470

[149] Troncarelli Flores, B. C. et al. Molecular and kinetic analyses of circulating tumor cells as predictive markers of treatment response in locally advanced rectal cancer patients. *Cells* **8**, 641 (2019). 31247977 10.3390/cells8070641 PMC6679115

[150] Colorectal Cancer Expert Committee of Chinese Society of Clinical Oncology (CSCO). [Consensus of Chinese experts on clinical detection of molecular markers of colorectal cancer]. *Zhonghua. Wei Chang Wai Ke Za Zhi* **24**, 191–197 (2021). 10.3760/cma.j.cn.441530-20201225-00679 34645160

[151] Osumi, H., Shinozaki, E., Yamaguchi, K. & Zembutsu, H. Early change in circulating tumor DNA as a potential predictor of response to chemotherapy in patients with metastatic colorectal cancer. *Sci. Rep.* **9**, 17358 (2019). 31758080 10.1038/s41598-019-53711-3 PMC6874682

[152] Tie, J. et al. Prognostic significance of postsurgery circulating tumor DNA in nonmetastatic colorectal cancer: Individual patient pooled analysis of three cohort studies. *Int. J. Cancer* **148**, 1014–1026 (2021). 32984952 10.1002/ijc.33312 PMC8818130

[153] (!!! INVALID CITATION !!!)..

[154] Halbrook, C. J., Lyssiotis, C. A., Pasca di Magliano, M. & Maitra, A. Pancreatic cancer: advances and challenges. *Cell* **186**, 1729–1754 (2023). 37059070 10.1016/j.cell.2023.02.014 PMC10182830

[155] Stosic, K. et al. A comprehensive review of the potential role of liquid biopsy as a diagnostic, prognostic, and predictive biomarker in pancreatic ductal adenocarcinoma. *Cells* **13**, 3 (2023). 38201207 10.3390/cells13010003 PMC10778087

[156] Ankeny, J. S. et al. Circulating tumour cells as a biomarker for diagnosis and staging in pancreatic cancer. *Br. J. Cancer* **114**, 1367–1375 (2016). 27300108 10.1038/bjc.2016.121 PMC4984454

[157] Early Diagnosis and Treatment Group, the Oncology Committee of Chinese Medical Association. [Expert consensus of oncology committee of Chinese medical association in early diagnosis and treatment of pancreatic cancer]. *Zhonghua Zhong Liu Za Zhi* **42**, 706–712 (2020). 32988150 10.3760/cma.j.cn112152-20200508-00426

[158] Rhim, A. D. et al. EMT and dissemination precede pancreatic tumor formation. *Cell* **148**, 349–361 (2012). 22265420 10.1016/j.cell.2011.11.025 PMC3266542

[159] Wang, R. et al. Diagnostic and prognostic values of KRAS mutations on EUS-FNA specimens and circulating tumor DNA in patients with pancreatic cancer. *Clin. Transl. Gastroenterol.* **13**, e00487 (2022). 35351843 10.14309/ctg.0000000000000487 PMC9132521

[160] Razavi, P. et al. High-intensity sequencing reveals the sources of plasma circulating cell-free DNA variants. *Nat. Med.* **25**, 1928–1937 (2019). 31768066 10.1038/s41591-019-0652-7 PMC7061455

[161] Cohen, J. D. et al. Combined circulating tumor DNA and protein biomarker-based liquid biopsy for the earlier detection of pancreatic cancers. *Proc. Natl. Acad. Sci. USA* **114**, 10202–10207 (2017). 28874546 10.1073/pnas.1704961114 PMC5617273

[162] Eissa, M. A. L. et al. Promoter methylation of ADAMTS1 and BNC1 as potential biomarkers for early detection of pancreatic cancer in blood. *Clin. Epigenet.* **11**, 59 (2019). 10.1186/s13148-019-0650-0 PMC6451253 30953539

[163] Wnuk, J., Strzelczyk, J. K. & Gisterek, I. Clinical value of circulating miRNA in diagnosis, prognosis, screening and monitoring therapy of pancreatic ductal adenocarcinoma-a review of the literature. *Int. J. Mol. Sci.* **24**, 5113 (2023). 36982210 10.3390/ijms24065113 PMC10049684

[164] Mann, D. V., Edwards, R., Ho, S., Lau, W. Y. & Glazer, G. Elevated tumour marker CA19-9: clinical interpretation and influence of obstructive jaundice. *Eur. J. Surg. Oncol.* **26**, 474–479 (2000). 11016469 10.1053/ejso.1999.0925

[165] Dittmar, R. L. et al. Plasma miRNA biomarkers in limited volume samples for detection of early-stage pancreatic cancer. *Cancer Prev. Res.* **14**, 729–740 (2021). 10.1158/1940-6207.CAPR-20-0303 PMC8818322 33893071

[166] Yu, S. et al. Plasma extracellular vesicle long RNA profiling identifies a diagnostic signature for the detection of pancreatic ductal adenocarcinoma. *Gut* **69**, 540–550 (2020). 31562239 10.1136/gutjnl-2019-318860

[167] Yeo, D. et al. Exploring the clinical utility of pancreatic cancer circulating tumor cells. *Int. J. Mol. Sci.* **23**, 1671 (2022). 35163592 10.3390/ijms23031671 PMC8836025

[168] Kim, H. et al. Clinical significance of circulating tumor cells after chemotherapy in unresectable pancreatic ductal adenocarcinoma. *Transl. Oncol.* **16**, 101321 (2022). 34954457 10.1016/j.tranon.2021.101321 PMC8718659

[169] Fusi, A. et al. Expression of chemokine receptors on circulating tumor cells in patients with solid tumors. *J. Transl. Med.* **10**, 52 (2012). 22433180 10.1186/1479-5876-10-52 PMC3337808

[170] Gardner, K. P., Tsai, S., Aldakkak, M., Gironda, S. & Adams, D. L. CXCR4 expression in tumor associated cells in blood is prognostic for progression and survival in pancreatic cancer. *PLoS ONE* **17**, e0264763 (2022). 35259193 10.1371/journal.pone.0264763 PMC8903256

[171] Groot, V. P. et al. Circulating tumor DNA as a clinical test in resected pancreatic cancer. *Clin. Cancer Res.* **25**, 4973–4984 (2019). 31142500 10.1158/1078-0432.CCR-19-0197 PMC7403524

[172] Wei, T. et al. Monitoring tumor burden in response to FOLFIRINOX chemotherapy via profiling circulating cell-free DNA in pancreatic cancer. *Mol. Cancer Ther.* **18**, 196–203 (2019). 30301865 10.1158/1535-7163.MCT-17-1298

[173] Lu, H. et al. MiR-20a-5p regulates gemcitabine chemosensitivity by targeting RRM2 in pancreatic cancer cells and serves as a predictor for gemcitabine-based chemotherapy. *Biosci. Rep.* **39**, BSR20181374 (2019). 30777929 10.1042/BSR20181374 PMC6504660

[174] van der Sijde, F. et al. Serum miR-373-3p and miR-194-5p are associated with early tumor progression during FOLFIRINOX treatment in pancreatic cancer patients: a prospective multicenter study. *Int. J. Mol. Sci.* **22**, 10902 (2021). 34681562 10.3390/ijms222010902 PMC8535910

[175] Mikamori, M. et al. MicroRNA-155 controls exosome synthesis and promotes gemcitabine resistance in pancreatic ductal adenocarcinoma. *Sci. Rep.* **7**, 42339 (2017). 28198398 10.1038/srep42339 PMC5309735

[176] Song, B. G. et al. Detection of circulating tumor cells in resectable pancreatic ductal adenocarcinoma: a prospective evaluation as a prognostic marker. *Front. Oncol.* **10**, 616440 (2020). 33680936 10.3389/fonc.2020.616440 PMC7930477

[177] Nitschke, C. et al. Characterization of RARRES1 expression on circulating tumor cells as unfavorable prognostic marker in resected pancreatic ductal adenocarcinoma patients. *Cancers* **14**, 4405 (2022). 36139565 10.3390/cancers14184405 PMC9497091

[178] Ako, S. et al. Plasma KRAS mutations predict the early recurrence after surgical resection of pancreatic cancer. *Cancer Biol. Ther.* **22**, 564–570 (2021). 34632919 10.1080/15384047.2021.1980312 PMC8726677

[179] Guo, S. et al. Preoperative detection of KRAS G12D mutation in ctDNA is a powerful predictor for early recurrence of resectable PDAC patients. *Br. J. Cancer* **122**, 857–867 (2020). 31969677 10.1038/s41416-019-0704-2 PMC7078253

[180] Kandimalla, R. et al. Identification of Serum miRNA signature and establishment of a nomogram for risk stratification in patients with pancreatic ductal adenocarcinoma. *Ann. Surg.* **275**, e229–e237 (2022). 32398486 10.1097/SLA.0000000000003945 PMC7648727

[181] Yang, Z. et al. A multianalyte panel consisting of extracellular vesicle miRNAs and mRNAs, cfDNA, and CA19-9 shows utility for diagnosis and staging of pancreatic ductal adenocarcinoma. *Clin. Cancer Res.* **26**, 3248–3258 (2020). 32299821 10.1158/1078-0432.CCR-19-3313 PMC7334066

[182] Dbouk, M. et al. Diagnostic performance of a tumor marker gene test to personalize serum CA19-9 reference ranges. *Clin. Cancer Res.* **29**, 4178–4185 (2023). 37566230 10.1158/1078-0432.CCR-23-0655 PMC10570677

[183] Ando, Y. et al. Using tumor marker gene variants to improve the diagnostic accuracy of dupan-2 and carbohydrate antigen 19-9 for pancreatic cancer. *J. Clin. Oncol.* **42**, 2196–2206 (2024). 38457748 10.1200/JCO.23.01573 PMC11191066

[184] López, M. J. et al. Characteristics of gastric cancer around the world. *Crit. Rev. Oncol. Hematol.* **181**, 103841 (2023). 36240980 10.1016/j.critrevonc.2022.103841

[185] Tang, L. et al. Diagnostic accuracy of circulating tumor cells detection in gastric cancer: systematic review and meta-analysis. *BMC Cancer* **13**, 314 (2013). 23806209 10.1186/1471-2407-13-314 PMC3699416

[186] Park, J. L. et al. Quantitative analysis of cell-free DNA in the plasma of gastric cancer patients. *Oncol. Lett.* **3**, 921–926 (2012). 22741019 10.3892/ol.2012.592 PMC3362424

[187] Kim, K. et al. Circulating cell-free DNA as a promising biomarker in patients with gastric cancer: diagnostic validity and significant reduction of cfDNA after surgical resection. *Ann. Surg. Treat. Res.* **86**, 136–142 (2014). 24761422 10.4174/astr.2014.86.3.136 PMC3994618

[188] Ren, J. et al. Genome-scale methylation analysis of circulating cell-free DNA in gastric cancer patients. *Clin. Chem.* **68**, 354–364 (2022). 34791072 10.1093/clinchem/hvab204

[189] Ko, K. et al. Methylation status and long-fragment cell-free DNA are prognostic biomarkers for gastric cancer. *Cancer Med.* **10**, 2003–2012 (2021). 33641249 10.1002/cam4.3755 PMC7957189

[190] Ma, S. et al. As a biomarker for gastric cancer, circPTPN22 regulates the progression of gastric cancer through the EMT pathway. *Cancer Cell Int.* **21**, 44 (2021). 33430866 10.1186/s12935-020-01701-1 PMC7802183

[191] Roy, S. et al. Diagnostic efficacy of circular RNAs as noninvasive, liquid biopsy biomarkers for early detection of gastric cancer. *Mol. Cancer* **21**, 42 (2022). 35139874 10.1186/s12943-022-01527-7 PMC8826675

[192] Xu, Y. et al. Clinical role of miR-421 as a novel biomarker in diagnosis of gastric cancer patients: a meta-analysis. *Medecine* **101**, e29242 (2022). 10.1097/MD.0000000000029242 PMC9276225 35583533

[193] Fu, H. et al. Exosomal TRIM3 is a novel marker and therapy target for gastric cancer. *J. Exp. Clin. Cancer Res.* **37**, 162 (2018). 30031392 10.1186/s13046-018-0825-0 PMC6054744

[194] Ito, H. et al. Long-term prognostic impact of circulating tumour cells in gastric cancer patients. *World J. Gastroenterol.* **22**, 10232–10241 (2016). 28028372 10.3748/wjg.v22.i46.10232 PMC5155183

[195] Huang, X. et al. Clinicopathological and prognostic significance of circulating tumor cells in patients with gastric cancer: a meta-analysis. *Int. J. Cancer* **136**, 21–33 (2015). 24803400 10.1002/ijc.28954

[196] Negishi, R. et al. Transcriptomic profiling of single circulating tumor cells provides insight into human metastatic gastric cancer. *Commun. Biol.* **5**, 20 (2022). 35017627 10.1038/s42003-021-02937-x PMC8752828

[197] Hiraiwa, K. et al. Clinical significance of circulating tumor cells in blood from patients with gastrointestinal cancers. *Ann. Surg. Oncol.* **15**, 3092–3100 (2008). 18766405 10.1245/s10434-008-0122-9

[198] Zhong, Y. et al. Plasma cfDNA as a potential biomarker to evaluate the efficacy of chemotherapy in gastric cancer. *Cancer Manag Res.* **12**, 3099–3106 (2020). 32440208 10.2147/CMAR.S243320 PMC7211302

[199] Qian, C. et al. Alu-based cell-free DNA: a novel biomarker for screening of gastric cancer. *Oncotarget* **8**, 54037–54045 (2017). 28903321 10.18632/oncotarget.11079 PMC5589560

[200] Bae, W. J. et al. miR-4742-5p promotes invasiveness of gastric cancer via targeting Rab43: An in vitro study. *Biochem. Biophys. Res. Commun.* **613**, 180–186 (2022). 35597125 10.1016/j.bbrc.2022.05.044

[201] Yifei, S., Chunxiao, H. & Dinuo, L. MiR-17-5p inhibits the proliferation and metastasis of gastric cancer cells by targeting PTEN protein. *Alter. Ther. Health Med.* **28**, 23–29 (2022). 35839114

[202] Cai, Y. et al. YncRNA PTCSC3 and lncRNA HULC Negatively affect each other to regulate cancer cell invasion and migration in gastric cancer [Retraction]. *Cancer Manag. Res.* **13**, 8003–8004 (2021). 10.2147/CMAR.S344967 PMC8544115 34707406

[203] Zheng, P., Gao, H., Xie, X. & Lu, P. Plasma exosomal hsa_circ_0015286 as a potential diagnostic and prognostic biomarker for gastric cancer. *Pathol. Oncol. Res.* **28**, 1610446 (2022). 35755416 10.3389/pore.2022.1610446 PMC9218071

[204] Cheng, B. et al. Enumeration and characterization of circulating tumor cells and its application in advanced gastric cancer. *Onco Targets Ther.* **12**, 7887–7896 (2019). 31576146 10.2147/OTT.S223222 PMC6768312

[205] Willis, J. et al. Validation of microsatellite instability detection using a comprehensive plasma-based genotyping panel. *Clin. Cancer Res.* **25**, 7035–7045 (2019). 31383735 10.1158/1078-0432.CCR-19-1324

[206] Azimi, M., Totonchi, M. & Ebrahimi, M. Determining the role of MicroRNAs in self-renewal, metastasis and resistance to drugs in human gastric cancer based on data mining approaches: a systematic review. *Cell J.* **24**, 1–6 (2022). 35182058 10.22074/cellj.2022.7173 PMC8876259

[207] Abbasi, A., Hosseinpourfeizi, M. & Safaralizadeh, R. All-trans retinoic acid-mediated miR-30a up-regulation suppresses autophagy and sensitizes gastric cancer cells to cisplatin. *Life Sci.* **307**, 120884 (2022). 35973456 10.1016/j.lfs.2022.120884

[208] Zhou, F. et al. The regulation of hsacirc_004413 promotes proliferation and drug resistance of gastric cancer cells by acting as a competing endogenous RNA for miR-145-5p. *PeerJ* **10**, e12629 (2022). 35415017 10.7717/peerj.12629 PMC8995023

[209] Fang, L. et al. Circular CPM promotes chemoresistance of gastric cancer via activating PRKAA2-mediated autophagy. *Clin. Transl. Med.* **12**, e708 (2022). 35075806 10.1002/ctm2.708 PMC8787023

[210] Zhou, H., Shen, W., Zou, H., Lv, Q. & Shao, P. Circulating exosomal long non-coding RNA H19 as a potential novel diagnostic and prognostic biomarker for gastric cancer. *J. Int. Med. Res.* **48**, 300060520934297 (2020). 32660285 10.1177/0300060520934297 PMC7361491

[211] Matsusaka, S. et al. Circulating tumor cells as a surrogate marker for determining response to chemotherapy in patients with advanced gastric cancer. *Cancer Sci.* **101**, 1067–1071 (2010). 20219073 10.1111/j.1349-7006.2010.01492.x PMC11159155

[212] Yu, P. et al. Application of circulating tumor cells and circulating free DNA from peripheral blood in the prognosis of advanced gastric cancer. *J. Oncol.* **2022**, 9635218 (2022). 35058982 10.1155/2022/9635218 PMC8766178

[213] Normando, S. R. C. et al. Circulating free plasma tumor DNA in patients with advanced gastric cancer receiving systemic chemotherapy. *BMC Clin. Pathol.* **18**, 12 (2018). 30498396 10.1186/s12907-018-0079-y PMC6258437

[214] Lan, Y. T. et al. Clinical relevance of cell-free DNA in gastrointestinal tract malignancy. *Oncotarget* **8**, 3009–3017 (2017). 27936467 10.18632/oncotarget.13821 PMC5356859

[215] Karamitrousis, E. I. et al. Prognostic Role of RASSF1A, SOX17 and Wif-1 promoter methylation status in cell-free DNA of advanced gastric cancer patients. *Technol. Cancer Res. Treat.* **20**, 1533033820973279 (2021). 33928818 10.1177/1533033820973279 PMC8113658

[216] Nicolazzo, C. et al. True conversions from RAS mutant to RAS wild-type in circulating tumor DNA from metastatic colorectal cancer patients as assessed by methylation and mutational signature. *Cancer Lett.* **507**, 89–96 (2021). 33744389 10.1016/j.canlet.2021.03.014

[217] Vrba, L. et al. DNA methylation biomarkers discovered in silico detect cancer in liquid biopsies from non-small cell lung cancer patients. *Epigenetics* **15**, 419–430 (2020). 31775567 10.1080/15592294.2019.1695333 PMC7153541

[218] Ooki, A. et al. A panel of novel detection and prognostic methylated DNA markers in primary non-small cell lung cancer and serum DNA. *Clin. Cancer Res.* **23**, 7141–7152 (2017). 28855354 10.1158/1078-0432.CCR-17-1222

[219] Yang, Z. et al. DNA methylation analysis of selected genes for the detection of early-stage lung cancer using circulating cell-free DNA. *Adv. Clin. Exp. Med.* **28**, 355–360 (2019). 30516882 10.17219/acem/84935

[220] Kang, S. M. et al. The Haptoglobin β chain as a supportive biomarker for human lung cancers. *Mol. Biosyst.* **7**, 1167–1175 (2011). 21253648 10.1039/c0mb00242a

[221] Sung, H. J. et al. Identification and validation of SAA as a potential lung cancer biomarker and its involvement in metastatic pathogenesis of lung cancer. *J. Proteome Res.* **10**, 1383–1395 (2011). 21141971 10.1021/pr101154j

[222] Cabanero, M. & Tsao, M. S. Circulating tumour DNA in EGFR-mutant non-small-cell lung cancer. *Curr. Oncol.* **25**, S38–s44 (2018). 29910646 10.3747/co.25.3761 PMC6001764

[223] Revelo, A. E. et al. Liquid biopsy for lung cancers: an update on recent developments. *Ann. Transl. Med.* **7**, 349 (2019). 31516895 10.21037/atm.2019.03.28 PMC6712255

[224] Li, R. Y. & Liang, Z. Y. Circulating tumor DNA in lung cancer: real-time monitoring of disease evolution and treatment response. *Chin. Med. J.* **133**, 2476–2485 (2020). 32960843 10.1097/CM9.0000000000001097 PMC7575184

[225] Zhang, Z. et al. Relationship between circulating tumour cell count and prognosis following chemotherapy in patients with advanced non-small-cell lung cancer. *Respirology* **21**, 519–525 (2016). 26661896 10.1111/resp.12696

[226] Huang, J. et al. Deregulation of serum microRNA expression is associated with cigarette smoking and lung cancer. *Biomed. Res. Int.* **2014**, 364316 (2014). 25386559 10.1155/2014/364316 PMC4217347

[227] Rodríguez, M. et al. Different exosome cargo from plasma/bronchoalveolar lavage in non-small-cell lung cancer. *Genes Chromosomes Cancer* **53**, 713–724 (2014). 24764226 10.1002/gcc.22181

[228] Rabinowits, G., Gerçel-Taylor, C., Day, J. M., Taylor, D. D. & Kloecker, G. H. Exosomal microRNA: a diagnostic marker for lung cancer. *Clin. Lung Cancer* **10**, 42–46 (2009). 19289371 10.3816/CLC.2009.n.006

[229] Szczerba, B. M. et al. Neutrophils escort circulating tumour cells to enable cell cycle progression. *Nature* **566**, 553–557 (2019). 30728496 10.1038/s41586-019-0915-y

[230] Rolfo, C. et al. Liquid biopsy for advanced NSCLC: a consensus statement from the International Association for the study of Lung Cancer. *J. Thorac. Oncol.* **16**, 1647–1662 (2021). 34246791 10.1016/j.jtho.2021.06.017

[231] Lee, D. H. Treatments for EGFR-mutant non-small cell lung cancer (NSCLC): the road to a success, paved with failures. *Pharm. Ther.* **174**, 1–21 (2017). 10.1016/j.pharmthera.2017.02.001 28167215

[232] Sung, H. et al. Global cancer statistics 2020: GLOBOCAN estimates of incidence and mortality worldwide for 36 cancers in 185 countries. *CA Cancer J. Clin.* **71**, 209–249 (2021). 33538338 10.3322/caac.21660

[233] Iacob, R. et al. Liquid biopsy in squamous cell carcinoma of the esophagus and of the head and neck. *Front. Med.* **9**, 827297 (2022). 10.3389/fmed.2022.827297 PMC9098838 35572996

[234] Yang, W. Y. et al. Liquid biopsy in head and neck squamous cell carcinoma: circulating tumor cells, circulating tumor DNA, and exosomes. *Expert Rev. Mol. Diagn.* **20**, 1213–1227 (2020). 33232189 10.1080/14737159.2020.1855977

[235] Payne, K. et al. Circulating tumor DNA as a biomarker and liquid biopsy in head and neck squamous cell carcinoma. *Head. Neck* **40**, 1598–1604 (2018). 29542214 10.1002/hed.25140

[236] Yu, S. et al. Oral-microbiome-derived signatures enable non-invasive diagnosis of laryngeal cancers. *J. Transl. Med.* **21**, 438 (2023). 37408030 10.1186/s12967-023-04285-2 PMC10320982

[237] Kawada, T. et al. Circulating tumor cells in patients with head and neck squamous cell carcinoma: feasibility of detection and quantitation. *Head. Neck* **39**, 2180–2186 (2017). 28815839 10.1002/hed.24893

[238] Nichols, A. C. et al. Detection of circulating tumor cells in advanced head and neck cancer using the cell search system. *Head. Neck* **34**, 1440–1444 (2012). 22076949 10.1002/hed.21941

[239] Rizzo, M. I. et al. Detection of circulating tumor cells in patients with laryngeal cancer using screen cell: comparative pre- and post-operative analysis and association with prognosis. *Oncol. Lett.* **19**, 4183–4188 (2020). 32391112 10.3892/ol.2020.11528 PMC7204636

[240] Wang, Y. et al. Detection of somatic mutations and HPV in the saliva and plasma of patients with head and neck squamous cell carcinomas. *Sci. Transl. Med.* **7**, 293ra104 (2015). 26109104 10.1126/scitranslmed.aaa8507 PMC4587492

[241] Sanchez-Cespedes, M. et al. Gene promoter hypermethylation in tumors and serum of head and neck cancer patients. *Cancer Res.* **60**, 892–895 (2000). 10706101

[242] Schröck, A. et al. Free-circulating methylated DNA in blood for diagnosis, staging, prognosis, and monitoring of head and neck squamous cell carcinoma patients: an observational prospective cohort study. *Clin. Chem.* **63**, 1288–1296 (2017). 28515105 10.1373/clinchem.2016.270207

[243] Reddy, K. B. MicroRNA (miRNA) in cancer. *Cancer Cell Int.* **15**, 38 (2015). 25960691 10.1186/s12935-015-0185-1 PMC4424445

[244] Yu, X. & Li, Z. The role of microRNAs expression in laryngeal cancer. *Oncotarget* **6**, 23297–23305 (2015). 26079642 10.18632/oncotarget.4195 PMC4695119

[245] Wang, J. et al. Combined detection of serum exosomal miR-21 and HOTAIR as diagnostic and prognostic biomarkers for laryngeal squamous cell carcinoma. *Med. Oncol.* **31**, 148 (2014). 25099764 10.1007/s12032-014-0148-8

[246] Wang, J. L., Wang, X., Yang, D. & Shi, W. J. The expression of MicroRNA-155 in plasma and tissue is matched in human laryngeal squamous cell carcinoma. *Yonsei Med. J.* **57**, 298–305 (2016). 26847279 10.3349/ymj.2016.57.2.298 PMC4740519

[247] Powrózek, T. et al. miRNA-130a significantly improves accuracy of SGA nutritional assessment tool in prediction of malnutrition and cachexia in radiotherapy-treated head and neck cancer patients. *Cancers* **10**, 294 (2018). 30200243 10.3390/cancers10090294 PMC6162742

[248] Cao, Y. C. et al. Serum miR-632 is a potential marker for the diagnosis and prognosis in laryngeal squamous cell carcinoma. *Acta Otolaryngol.* **140**, 418–421 (2020). 32068453 10.1080/00016489.2020.1717610

[249] Hsu, C. L., Chang, Y. S. & Li, H. P. Molecular diagnosis of nasopharyngeal carcinoma: past and future. *Biomed. J*. 100748, 10.1016/j.bj.2024.100748 (2024).10.1016/j.bj.2024.10074838796105

[250] Tan, R. et al. Clinical utility of Epstein-Barr virus DNA and other liquid biopsy markers in nasopharyngeal carcinoma. *Cancer Commun.* **40**, 564–585 (2020). 10.1002/cac2.12100 PMC7668470 32989921

[251] Lo, Y. M. et al. Quantitative and temporal correlation between circulating cell-free Epstein-Barr virus DNA and tumor recurrence in nasopharyngeal carcinoma. *Cancer Res.* **59**, 5452–5455 (1999). 10554016

[252] Chan, K. A. et al. Analysis of plasma Epstein-Barr virus DNA to screen for nasopharyngeal cancer. *N. Engl. J. Med.* **378**, 973 (2018). 10.1056/NEJMx180004 29514021

[253] Hsu, C. L. et al. Plasma Epstein-Barr virus DNA concentration and clearance rate as novel prognostic factors for metastatic nasopharyngeal carcinoma. *Head Neck* **34**, 1064–1070 (2012). 22083949 10.1002/hed.21890

[254] Zheng, X. H. et al. Saliva biopsy: detecting the difference of EBV DNA methylation in the diagnosis of nasopharyngeal carcinoma. *Int. J. Cancer* **153**, 882–892 (2023). 37170851 10.1002/ijc.34561

[255] Wu, C. F. et al. Liquid biopsy posttreatment surveillance in endemic nasopharyngeal carcinoma: a cost-effective strategy to integrate circulating cell-free Epstein-Barr virus DNA. *BMC Med.* **19**, 193 (2021). 34433440 10.1186/s12916-021-02076-4 PMC8390246

[256] Sheu, L. F. et al. Enhanced malignant progression of nasopharyngeal carcinoma cells mediated by the expression of Epstein-Barr nuclear antigen 1 in vivo. *J. Pathol.* **180**, 243–248 (1996). 8958799 10.1002/(SICI)1096-9896(199611)180:3<243::AID-PATH655>3.0.CO;2-7

[257] Murono, S. et al. Detection of Epstein-Barr virus in nasopharyngeal carcinoma by in situ hybridization and polymerase chain reaction. *Laryngoscope* **107**, 523–526 (1997). 9111384 10.1097/00005537-199704000-00017

[258] Wong, A. M., Kong, K. L., Tsang, J. W., Kwong, D. L. & Guan, X. Y. Profiling of Epstein-Barr virus-encoded microRNAs in nasopharyngeal carcinoma reveals potential biomarkers and oncomirs. *Cancer* **118**, 698–710 (2012). 21720996 10.1002/cncr.26309

[259] Stenvang, J., Petri, A., Lindow, M., Obad, S. & Kauppinen, S. Inhibition of microRNA function by antimiR oligonucleotides. *Silence* **3**, 1 (2012). 22230293 10.1186/1758-907X-3-1 PMC3306207

[260] Zhao, Z. et al. Applications of cerebrospinal fluid circulating tumor DNA in the diagnosis of gliomas. *Jpn. J. Clin. Oncol.* **50**, 325–332 (2020). 32039443 10.1093/jjco/hyz156

[261] Borba, L. A. B., Passos, G. & Oliveira, I. Liquid biopsy and tumor DNA/RNA detection in the cerebrospinal fluid of patients diagnosed with central nervous system glioma—a review article. *Surg. Neurol. Int.* **14**, 183 (2023). 37292399 10.25259/SNI_52_2023 PMC10246314

[262] Li, K. et al. Imaging and liquid biopsy for distinguishing true progression from pseudoprogression in gliomas, current advances and challenges. *Acad. Radiol.* **31**, 3366–3383 (2024). 38614827 10.1016/j.acra.2024.03.019

[263] Thege, F. I. et al. Microfluidic immunocapture of circulating pancreatic cells using parallel EpCAM and MUC1 capture: characterization, optimization and downstream analysis. *Lab Chip* **14**, 1775–1784 (2014). 24681997 10.1039/c4lc00041b

[264] Sullivan, J. P. et al. Brain tumor cells in circulation are enriched for mesenchymal gene expression. *Cancer Discov.* **4**, 1299–1309 (2014). 25139148 10.1158/2159-8290.CD-14-0471 PMC4221467

[265] Wick, M., Gross, C. C., Isenmann, S. & Strik, H. Cytology of cerebrospinal fluid: standards, importance and modern methods. *Nervenarzt* **87**, 1276–1281 (2016). 27649988 10.1007/s00115-016-0219-5

[266] Nabors, L. B. et al. Central nervous system cancers, version 3.2020, NCCN clinical practice guidelines in oncology. *J. Natl. Compr. Cancer Netw.* **18**, 1537–1570 (2020). 10.6004/jnccn.2020.0052 33152694

[267] De Mattos-Arruda, L. et al. Cerebrospinal fluid-derived circulating tumour DNA better represents the genomic alterations of brain tumours than plasma. *Nat. Commun.* **6**, 8839 (2015). 26554728 10.1038/ncomms9839 PMC5426516

[268] Orzan, F. et al. Liquid biopsy of cerebrospinal fluid enables selective profiling of glioma molecular subtypes at first clinical presentation. *Clin. Cancer Res.* **29**, 1252–1266 (2023). 36648487 10.1158/1078-0432.CCR-22-2903 PMC10068436

[269] Macarthur, K. M. et al. Detection of brain tumor cells in the peripheral blood by a telomerase promoter-based assay. *Cancer Res.* **74**, 2152–2159 (2014). 24525740 10.1158/0008-5472.CAN-13-0813 PMC4144786

[270] Müller, C. et al. Hematogenous dissemination of glioblastoma multiforme. *Sci. Transl. Med.* **6**, 247ra101 (2014). 25080476 10.1126/scitranslmed.3009095

[271] Anfossi, S., Babayan, A., Pantel, K. & Calin, G. A. Clinical utility of circulating non-coding RNAs—an update. *Nat. Rev. Clin. Oncol.* **15**, 541–563 (2018). 29784926 10.1038/s41571-018-0035-x

[272] Wierzbicki, K. et al. Targeting and therapeutic monitoring of H3K27M-mutant glioma. *Curr. Oncol. Rep.* **22**, 19 (2020). 32030483 10.1007/s11912-020-0877-0 PMC7501595

[273] Bettegowda, C. et al. Detection of circulating tumor DNA in early- and late-stage human malignancies. *Sci. Transl. Med.* **6**, 224ra224 (2014). 10.1126/scitranslmed.3007094 PMC4017867 24553385

[274] Ghodsi, M., Shahmohammadi, M., Modarressi, M. H. & Karami, F. Investigation of promoter methylation of MCPH1 gene in circulating cell-free DNA of brain tumor patients. *Exp. Brain Res.* **238**, 1903–1909 (2020). 32556427 10.1007/s00221-020-05848-1

[275] Westphal, M. & Lamszus, K. Circulating biomarkers for gliomas. *Nat. Rev. Neurol.* **11**, 556–566 (2015). 26369507 10.1038/nrneurol.2015.171

[276] Manterola, L. et al. A small noncoding RNA signature found in exosomes of GBM patient serum as a diagnostic tool. *Neuro Oncol.* **16**, 520–527 (2014). 24435880 10.1093/neuonc/not218 PMC3956347

[277] Akers, J. C. et al. A cerebrospinal fluid microRNA signature as biomarker for glioblastoma. *Oncotarget* **8**, 68769–68779 (2017). 28978155 10.18632/oncotarget.18332 PMC5620295

[278] Shao, H. et al. Protein typing of circulating microvesicles allows real-time monitoring of glioblastoma therapy. *Nat. Med.* **18**, 1835–1840 (2012). 23142818 10.1038/nm.2994 PMC3518564

[279] Setti, M. et al. Extracellular vesicle-mediated transfer of CLIC1 protein is a novel mechanism for the regulation of glioblastoma growth. *Oncotarget* **6**, 31413–31427 (2015). 26429879 10.18632/oncotarget.5105 PMC4741615

[280] Hiemcke-Jiwa, L. S. et al. Molecular analysis in liquid biopsies for diagnostics of primary central nervous system lymphoma: review of literature and future opportunities. *Crit. Rev. Oncol. Hematol.* **127**, 56–65 (2018). 29891112 10.1016/j.critrevonc.2018.05.010

[281] Baraniskin, A. & Schroers, R. Modern cerebrospinal fluid analyses for the diagnosis of diffuse large B-cell lymphoma of the CNS. *CNS Oncol.* **3**, 77–85 (2014). 25054902 10.2217/cns.13.63 PMC6128195

[282] Akhter, A. et al. Differential expression of Toll-like receptor (TLR) and B cell receptor (BCR) signaling molecules in primary diffuse large B-cell lymphoma of the central nervous system. *J. Neurooncol.* **121**, 289–296 (2015). 25391967 10.1007/s11060-014-1655-3

[283] Chapuy, B. et al. Targetable genetic features of primary testicular and primary central nervous system lymphomas. *Blood* **127**, 869–881 (2016). 26702065 10.1182/blood-2015-10-673236 PMC4760091

[284] Yamaguchi, J. et al. Rapid detection of the MYD88 L265P mutation for pre- and intra-operative diagnosis of primary central nervous system lymphoma. *Cancer Sci.* **114**, 2544–2551 (2023). 36859777 10.1111/cas.15762 PMC10236607

[285] Hiemcke-Jiwa, L. S. et al. The use of droplet digital PCR in liquid biopsies: a highly sensitive technique for MYD88 p.(L265P) detection in cerebrospinal fluid. *Hematol. Oncol.* **36**, 429–435 (2018). 29210102 10.1002/hon.2489

[286] Zorofchian, S. et al. Detection of the MYD88 p.L265P mutation in the CSF of a patient with secondary central nervous system lymphoma. *Front. Oncol.* **8**, 382 (2018). 30294590 10.3389/fonc.2018.00382 PMC6158312

[287] Fontanilles, M. et al. Non-invasive detection of somatic mutations using next-generation sequencing in primary central nervous system lymphoma. *Oncotarget* **8**, 48157–48168 (2017). 28636991 10.18632/oncotarget.18325 PMC5564634

[288] Hou, Y., Zi, J., Liu, S., Ge, Q. & Ge, Z. Mutational profiling of circulating tumor DNA and clinical characteristics in lymphoma: based on next generation sequencing. *Mol. Carcinog.* **62**, 200–209 (2023). 36300887 10.1002/mc.23476

[289] Hu, Y. et al. Exosomal miR-200c and miR-141 as cerebrospinal fluid biopsy biomarkers for the response to chemotherapy in primary central nervous system lymphoma. *Discov. Oncol.* **14**, 205 (2023). 37971595 10.1007/s12672-023-00812-1 PMC10654293

[290] Mathieu, M., Martin-Jaular, L., Lavieu, G. & Théry, C. Specificities of secretion and uptake of exosomes and other extracellular vesicles for cell-to-cell communication. *Nat. Cell Biol.* **21**, 9–17 (2019). 30602770 10.1038/s41556-018-0250-9

[291] Deng, Y. et al. Phosphoproteome analysis of cerebrospinal fluid extracellular vesicles in primary central nervous system lymphoma. *Analyst* **148**, 3594–3602 (2023). 37403840 10.1039/d3an00670k

[292] Ikeguchi, R., Shimizu, Y., Shimizu, S. & Kitagawa, K. CSF and clinical data are useful in differentiating CNS inflammatory demyelinating disease from CNS lymphoma. *Mult. Scler.* **24**, 1212–1223 (2018). 28657431 10.1177/1352458517717804

[293] Sasagawa, Y., Akai, T., Tachibana, O. & Iizuka, H. Diagnostic value of interleukin-10 in cerebrospinal fluid for diffuse large B-cell lymphoma of the central nervous system. *J. Neurooncol.* **121**, 177–183 (2015). 25258254 10.1007/s11060-014-1622-z

[294] Shao, J. et al. High level of IL-10 in cerebrospinal fluid is specific for diagnosis of primary central nervous system lymphoma. *Cancer Manag Res.* **12**, 6261–6268 (2020). 32801871 10.2147/CMAR.S255482 PMC7386815

[295] Viaccoz, A. et al. CSF neopterin level as a diagnostic marker in primary central nervous system lymphoma. *Neuro Oncol.* **17**, 1497–1503 (2015). 26014047 10.1093/neuonc/nov092 PMC4648303

[296] Kubiliute, R. & Jarmalaite, S. Epigenetic biomarkers of renal cell carcinoma for liquid biopsy tests. *Int. J. Mol. Sci.* **22**, 8846 (2021). 34445557 10.3390/ijms22168846 PMC8396354

[297] Li, M. et al. Liquid biopsy at the frontier in renal cell carcinoma: recent analysis of techniques and clinical application. *Mol. Cancer* **22**, 37 (2023). 36810071 10.1186/s12943-023-01745-7 PMC9942319

[298] Bade, R. M. et al. Development and initial clinical testing of a multiplexed circulating tumor cell assay in patients with clear cell renal cell carcinoma. *Mol. Oncol.* **15**, 2330–2344 (2021). 33604999 10.1002/1878-0261.12931 PMC8410529

[299] Nuzzo, P. V. et al. Detection of renal cell carcinoma using plasma and urine cell-free DNA methylomes. *Nat. Med.* **26**, 1041–1043 (2020). 32572266 10.1038/s41591-020-0933-1 PMC8288043

[300] Chen, X. et al. Identification of a four-microRNA panel in serum for screening renal cell carcinoma. *Pathol. Res. Pract.* **227**, 153625 (2021). 34628264 10.1016/j.prp.2021.153625

[301] Di Meo, A. et al. Prognostic urinary miRNAs for the assessment of small renal masses. *Clin. Biochem.* **75**, 15–22 (2020). 31672647 10.1016/j.clinbiochem.2019.10.002

[302] Heinemann, F. G. et al. Serum miR-122-5p and miR-206 expression: non-invasive prognostic biomarkers for renal cell carcinoma. *Clin. Epigenet.* **10**, 11 (2018). 10.1186/s13148-018-0444-9 PMC5781339 29410711

[303] Outeiro-Pinho, G. et al. MicroRNA-30a-5p(me): a novel diagnostic and prognostic biomarker for clear cell renal cell carcinoma in tissue and urine samples. *J. Exp. Clin. Cancer Res.* **39**, 98 (2020). 32487203 10.1186/s13046-020-01600-3 PMC7323611

[304] Di Meo, A. et al. Searching for prognostic biomarkers for small renal masses in the urinary proteome. *Int. J. Cancer* **146**, 2315–2325 (2020). 31465112 10.1002/ijc.32650

[305] Xu, W. et al. Plasma KIM-1 is associated with recurrence risk after nephrectomy for localized renal cell carcinoma: a trial of the ECOG-ACRIN Research Group (E2805). *Clin. Cancer Res.* **27**, 3397–3403 (2021). 33832947 10.1158/1078-0432.CCR-21-0025 PMC8287837

[306] Sato, T. et al. Accurate quantification of urinary metabolites for predictive models manifest clinicopathology of renal cell carcinoma. *Cancer Sci.* **111**, 2570–2578 (2020). 32350988 10.1111/cas.14440 PMC7385347

[307] Wang, Z. et al. UPLC-MS-based urine untargeted metabolomic analyses to differentiate bladder cancer from renal cell carcinoma. *BMC Cancer* **19**, 1195 (2019). 31805976 10.1186/s12885-019-6354-1 PMC6896793

[308] Yamamoto, Y. et al. Clinical significance of the mutational landscape and fragmentation of circulating tumor DNA in renal cell carcinoma. *Cancer Sci.* **110**, 617–628 (2019). 30536551 10.1111/cas.13906 PMC6361573

[309] Salinas-Sánchez, A. S. et al. Clinical value of perioperative levels of DNA and mRNA in plasma of patients with renal cell carcinoma. *Transl. Oncol.* **14**, 100999 (2021). 33348249 10.1016/j.tranon.2020.100999 PMC7750690

[310] Del Re, M. et al. The amount of DNA combined with TP53 mutations in liquid biopsy is associated with clinical outcome of renal cancer patients treated with immunotherapy and VEGFR-TKIs. *J. Transl. Med.* **20**, 371 (2022). 35974365 10.1186/s12967-022-03557-7 PMC9382729

[311] Bacon, J. V. W. et al. Plasma circulating tumor DNA and clonal hematopoiesis in metastatic renal cell carcinoma. *Clin. Genitourin. Cancer* **18**, 322–331.e322 (2020). 32046920 10.1016/j.clgc.2019.12.018

[312] Koh, Y. et al. Early dynamics of circulating tumor DNA predict clinical response to immune checkpoint inhibitors in metastatic renal cell carcinoma. *Int. J. Urol.* **29**, 462–469 (2022). 35184335 10.1111/iju.14816 PMC9306972

[313] Mytsyk, Y. et al. MicroRNA-15a expression measured in urine samples as a potential biomarker of renal cell carcinoma. *Int. Urol. Nephrol.* **50**, 851–859 (2018). 29549624 10.1007/s11255-018-1841-x

[314] Wang, Z. L. et al. Dynamic changes of different phenotypic and genetic circulating tumor cells as a biomarker for evaluating the prognosis of RCC. *Cancer Biol. Ther.* **20**, 505–512 (2019). 30359544 10.1080/15384047.2018.1537576 PMC6422478

[315] Zhang, W. et al. MicroRNAs in serum exosomes as potential biomarkers in clear-cell renal cell carcinoma. *Eur. Urol. Focus* **4**, 412–419 (2018). 28753793 10.1016/j.euf.2016.09.007

[316] Xiao, C. T., Lai, W. J., Zhu, W. A. & Wang, H. MicroRNA derived from circulating exosomes as noninvasive biomarkers for diagnosing renal cell carcinoma. *Onco Targets Ther.* **13**, 10765–10774 (2020). 33122915 10.2147/OTT.S271606 PMC7591082

[317] Guo, R. et al. LncRNA RCAT1 promotes tumor progression and metastasis via miR-214-5p/E2F2 axis in renal cell carcinoma. *Cell Death Dis.* **12**, 689 (2021). 34244473 10.1038/s41419-021-03955-7 PMC8270952

[318] Liu, H. et al. circPTCH1 promotes invasion and metastasis in renal cell carcinoma via regulating miR-485-5p/MMP14 axis. *Theranostics* **10**, 10791–10807 (2020). 32929380 10.7150/thno.47239 PMC7482820

[319] Li, Y. et al. Piwi-interacting RNAs (piRNAs) are dysregulated in renal cell carcinoma and associated with tumor metastasis and cancer-specific survival. *Mol. Med.* **21**, 381–388 (2015). 25998508 10.2119/molmed.2014.00203 PMC4534471

[320] Zhao, C. et al. Mitochondrial PIWI-interacting RNAs are novel biomarkers for clear cell renal cell carcinoma. *World J. Urol.* **37**, 1639–1647 (2019). 30488095 10.1007/s00345-018-2575-1

[321] Piao, X. M., Cha, E. J., Yun, S. J. & Kim, W. J. Role of exosomal miRNA in bladder cancer: a promising liquid biopsy biomarker. *Int. J. Mol. Sci.* **22**, 1713 (2021). 33567779 10.3390/ijms22041713 PMC7915637

[322] Li, S. et al. Blood-based liquid biopsy: insights into early detection, prediction, and treatment monitoring of bladder cancer. *Cell Mol. Biol. Lett.* **28**, 28 (2023). 37016296 10.1186/s11658-023-00442-z PMC10074703

[323] Qi, F. et al. Quantitation of rare circulating tumor cells by folate receptor α ligand-targeted PCR in bladder transitional cell carcinoma and its potential diagnostic significance. *Tumour Biol.* **35**, 7217–7223 (2014). 24771263 10.1007/s13277-014-1894-0

[324] Valenzuela, M. T. et al. Assessing the use of p16(INK4a) promoter gene methylation in serum for detection of bladder cancer. *Eur. Urol.* **42**, 622–628 (2002). 12477660 10.1016/s0302-2838(02)00468-2

[325] Ellinger, J. et al. Hypermethylation of cell-free serum DNA indicates worse outcome in patients with bladder cancer. *J. Urol.* **179**, 346–352 (2008). 18006010 10.1016/j.juro.2007.08.091

[326] Domínguez, G. et al. p14ARF promoter hypermethylation in plasma DNA as an indicator of disease recurrence in bladder cancer patients. *Clin. Cancer Res.* **8**, 980–985 (2002). 11948103

[327] Lin, Y. L., Sun, G., Liu, X. Q., Li, W. P. & Ma, J. G. Clinical significance of CDH13 promoter methylation in serum samples from patients with bladder transitional cell carcinoma. *J. Int. Med. Res.* **39**, 179–186 (2011). 21672320 10.1177/147323001103900119

[328] Feng, Y. et al. miR-19a acts as an oncogenic microRNA and is up-regulated in bladder cancer. *J. Exp. Clin. Cancer Res.* **33**, 67 (2014). 25107371 10.1186/s13046-014-0067-8 PMC4237814

[329] Adam, L. et al. Plasma microRNA profiles for bladder cancer detection. *Urol. Oncol.* **31**, 1701–1708 (2013). 22863868 10.1016/j.urolonc.2012.06.010 PMC5226073

[330] Jiang, X. et al. Serum microRNA expression signatures as novel noninvasive biomarkers for prediction and prognosis of muscle-invasive bladder cancer. *Oncotarget* **7**, 36733–36742 (2016). 27167342 10.18632/oncotarget.9166 PMC5095035

[331] Jiang, X. et al. Serum microRNA expression signatures identified from genome-wide microRNA profiling serve as novel noninvasive biomarkers for diagnosis and recurrence of bladder cancer. *Int. J. Cancer* **136**, 854–862 (2015). 24961907 10.1002/ijc.29041

[332] Liang, Z., Liu, L., Gao, R., Che, C. & Yang, G. Downregulation of exosomal miR-7-5p promotes breast cancer migration and invasion by targeting RYK and participating in the atypical WNT signalling pathway. *Cell Mol. Biol. Lett.* **27**, 88 (2022). 36210461 10.1186/s11658-022-00393-x PMC9549651

[333] Yin, X. et al. Exosomal miR-663b targets Ets2-repressor factor to promote proliferation and the epithelial-mesenchymal transition of bladder cancer cells. *Cell Biol. Int.* **44**, 958–965 (2020). 31872468 10.1002/cbin.11292

[334] Cai, Q. et al. Urine BLCA-4 exerts potential role in detecting patients with bladder cancers: a pooled analysis of individual studies. *Oncotarget* **6**, 37500–37510 (2015). 26462026 10.18632/oncotarget.6061 PMC4741944

[335] Roupret, M. et al. Diagnostic accuracy of MCM5 for the detection of recurrence in nonmuscle invasive bladder cancer followup: a blinded, prospective cohort, multicenter European study. *J. Urol.* **204**, 685–690 (2020). 32314931 10.1097/JU.0000000000001084

[336] Southgate, J., Harnden, P. & Trejdosiewicz, L. K. Cytokeratin expression patterns in normal and malignant urothelium: a review of the biological and diagnostic implications. *Histol. Histopathol.* **14**, 657–664 (1999). 10212826 10.14670/HH-14.657

[337] Hosen, M. I. et al. Urinary TERT promoter mutations are detectable up to 10 years prior to clinical diagnosis of bladder cancer: evidence from the Golestan Cohort Study. *EBioMedicine* **53**, 102643 (2020). 32081602 10.1016/j.ebiom.2020.102643 PMC7118568

[338] Hernández, S. et al. Prospective study of FGFR3 mutations as a prognostic factor in nonmuscle invasive urothelial bladder carcinomas. *J. Clin. Oncol.* **24**, 3664–3671 (2006). 16877735 10.1200/JCO.2005.05.1771

[339] Haga, N. et al. Increase in circulating tumor cells in invasive bladder cancer after transurethral resection of bladder tumor. *Anticancer Res.* **40**, 4299–4307 (2020). 32727757 10.21873/anticanres.14432

[340] Gazzaniga, P. et al. Circulating tumor cells detection has independent prognostic impact in high-risk non-muscle invasive bladder cancer. *Int. J. Cancer* **135**, 1978–1982 (2014). 24599551 10.1002/ijc.28830

[341] Gazzaniga, P. et al. Prognostic value of circulating tumor cells in nonmuscle invasive bladder cancer: a CellSearch analysis. *Ann. Oncol.* **23**, 2352–2356 (2012). 22351740 10.1093/annonc/mdr619

[342] Vandekerkhove, G. et al. Plasma ctDNA is a tumor tissue surrogate and enables clinical-genomic stratification of metastatic bladder cancer. *Nat. Commun.* **12**, 184 (2021). 33420073 10.1038/s41467-020-20493-6 PMC7794518

[343] Raimondi, C., Gradilone, A. & Gazzaniga, P. Circulating tumor cells in early bladder cancer: insight into micrometastatic disease. *Expert Rev. Mol. Diagn.* **14**, 407–409 (2014). 24717055 10.1586/14737159.2014.908119

[344] Zhang, Z. et al. The prognostic and diagnostic value of circulating tumor cells in bladder cancer and upper tract urothelial carcinoma: a meta-analysis of 30 published studies. *Oncotarget* **8**, 59527–59538 (2017). 28938656 10.18632/oncotarget.18521 PMC5601752

[345] Beije, N. et al. Circulating tumour cells to drive the use of neoadjuvant chemotherapy in patients with muscle-invasive bladder cancer. *ESMO Open* **7**, 100416 (2022). 35248823 10.1016/j.esmoop.2022.100416 PMC9058916

[346] Anantharaman, A. et al. Programmed death-ligand 1 (PD-L1) characterization of circulating tumor cells (CTCs) in muscle invasive and metastatic bladder cancer patients. *BMC Cancer* **16**, 744 (2016). 27658492 10.1186/s12885-016-2758-3 PMC5034508

[347] Corcoran, R. B. & Chabner, B. A. Application of cell-free DNA analysis to cancer treatment. *N. Engl. J. Med.* **379**, 1754–1765 (2018). 30380390 10.1056/NEJMra1706174

[348] Raja, R. et al. Early reduction in ctDNA predicts survival in patients with lung and bladder cancer treated with durvalumab. *Clin. Cancer Res.* **24**, 6212–6222 (2018). 30093454 10.1158/1078-0432.CCR-18-0386

[349] Shohdy, K. S. et al. Serial ctDNA analysis predicts clinical progression in patients with advanced urothelial carcinoma. *Br. J. Cancer* **126**, 430–439 (2022). 35046520 10.1038/s41416-021-01648-8 PMC8810988

[350] Birkenkamp-Demtröder, K. et al. Monitoring treatment response and metastatic relapse in advanced bladder cancer by liquid biopsy analysis. *Eur. Urol.* **73**, 535–540 (2018). 28958829 10.1016/j.eururo.2017.09.011

[351] de Kruijff, I. E. et al. Liquid biopsies to select patients for perioperative chemotherapy in muscle-invasive bladder cancer: a systematic review. *Eur. Urol. Oncol.* **4**, 204–214 (2021). 32059957 10.1016/j.euo.2020.01.003

[352] Powles, T. et al. ctDNA guiding adjuvant immunotherapy in urothelial carcinoma. *Nature* **595**, 432–437 (2021). 34135506 10.1038/s41586-021-03642-9

[353] Zhang, J. et al. Circulating tumor DNA analyses predict disease recurrence in non-muscle-invasive bladder cancer. *Front. Oncol.* **11**, 657483 (2021). 33996580 10.3389/fonc.2021.657483 PMC8114939

[354] Koguchi, D. et al. Diagnostic potential of circulating tumor cells, urinary MicroRNA, and urinary cell-free DNA for bladder cancer: a review. *Int. J. Mol. Sci.* **23**, 9148 (2022). 36012417 10.3390/ijms23169148 PMC9409245

[355] Zheng, H., Liu, J., Pan, X. & Cui, X. Biomarkers for patients with Wilms tumor: a review. *Front. Oncol.* **13**, 1137346 (2023). 37554168 10.3389/fonc.2023.1137346 PMC10405734

[356] Miguez, A. C. K. et al. Assessment of somatic mutations in urine and plasma of Wilms tumor patients. *Cancer Med.* **9**, 5948–5959 (2020). 32592321 10.1002/cam4.3236 PMC7433816

[357] Schmitt, J. et al. Treatment-independent miRNA signature in blood of Wilms tumor patients. *BMC Genom.* **13**, 379 (2012). 10.1186/1471-2164-13-379 PMC3563587 22871070

[358] Treger, T. D. et al. Somatic TP53 mutations are detectable in circulating tumor DNA from children with anaplastic Wilms tumors. *Transl. Oncol.* **11**, 1301–1306 (2018). 30172241 10.1016/j.tranon.2018.08.006 PMC6121832

[359] Madanat-Harjuoja, L. M. et al. Circulating tumor DNA as a biomarker in patients with stage III and IV Wilms tumor: analysis from a Children’s Oncology Group Trial, AREN0533. *J. Clin. Oncol.* **40**, 3047–3056 (2022). 35580298 10.1200/JCO.22.00098 PMC9462535

[360] Perotti, D. et al. Hallmark discoveries in the biology of Wilms tumour. *Nat. Rev. Urol.* **21**, 158–180 (2024). 37848532 10.1038/s41585-023-00824-0

[361] Stern, M., Longaker, M. T., Adzick, N. S., Harrison, M. R. & Stern, R. Hyaluronidase levels in urine from Wilms’ tumor patients. *J. Natl. Cancer Inst.* **83**, 1569–1574 (1991). 1660075 10.1093/jnci/83.21.1569

[362] Lin, R. Y., Argenta, P. A., Sullivan, K. M. & Adzick, N. S. Diagnostic and prognostic role of basic fibroblast growth factor in Wilms’ tumor patients. *Clin. Cancer Res.* **1**, 327–331 (1995). 9815988

[363] Padullés, B. et al. Prognostic value of liquid-biopsy-based biomarkers in upper tract urothelial carcinoma. *Int. J. Mol. Sci.* **25**, 3695 (2024). 38612507 10.3390/ijms25073695 PMC11012136

[364] Nakano, K. et al. Fragmentation of cell-free DNA is induced by upper-tract urothelial carcinoma-associated systemic inflammation. *Cancer Sci.* **112**, 168–177 (2021). 33027843 10.1111/cas.14679 PMC7780031

[365] Blumendeller, C. et al. Use of plasma ctDNA as a potential biomarker for longitudinal monitoring of a patient with metastatic high-risk upper tract urothelial carcinoma receiving pembrolizumab and personalized neoepitope-derived multipeptide vaccinations: a case report. *J. Immunother. Cancer* **9**, e001406 (2021). 33431630 10.1136/jitc-2020-001406 PMC7802705

[366] Springer, S. U. et al. Non-invasive detection of urothelial cancer through the analysis of driver gene mutations and aneuploidy. *eLife* **7**, e32143 (2018). 29557778 10.7554/eLife.32143 PMC5860864

[367] Ghoreifi, A. et al. A urine-based DNA methylation marker test to detect upper tract urothelial carcinoma: a prospective cohort study. *J. Urol.* **209**, 854–862 (2023). 36795966 10.1097/JU.0000000000003188

[368] Ge, G. et al. Urothelial carcinoma detection based on copy number profiles of urinary cell-free DNA by shallow whole-genome sequencing. *Clin. Chem.* **66**, 188–198 (2020). 31811000 10.1373/clinchem.2019.309633

[369] Lu, H. et al. Aristolochic acid mutational signature defines the low-risk subtype in upper tract urothelial carcinoma. *Theranostics* **10**, 4323–4333 (2020). 32292497 10.7150/thno.43251 PMC7150494

[370] Chalfin, H. J. et al. Circulating tumor cell and circulating tumor DNA assays reveal complementary information for patients with metastatic urothelial cancer. *Eur. Urol. Oncol.* **4**, 310–314 (2021). 31563523 10.1016/j.euo.2019.08.004

[371] Xu, Y. et al. A urine-based liquid biopsy method for detection of upper tract urinary carcinoma. *Front. Oncol.* **10**, 597486 (2020). 33634022 10.3389/fonc.2020.597486 PMC7901537

[372] Urabe, F. et al. Independent verification of circulating miRNA as diagnostic biomarkers for urothelial carcinoma. *Cancer Sci.* **113**, 3510–3517 (2022). 35848873 10.1111/cas.15496 PMC9530882

[373] Kriebel, S. et al. Analysis of tissue and serum microRNA expression in patients with upper urinary tract urothelial cancer. *PLoS ONE* **10**, e0117284 (2015). 25629698 10.1371/journal.pone.0117284 PMC4309610

[374] Montalbo, R. et al. Prognostic value of circulating microRNAs in upper tract urinary carcinoma. *Oncotarget* **9**, 16691–16700 (2018). 29682178 10.18632/oncotarget.24672 PMC5908279

[375] Li, Y. et al. Identification of plasma secreted phosphoprotein 1 as a novel biomarker for upper tract urothelial carcinomas. *Biomed. Pharmacother.* **113**, 108744 (2019). 30844659 10.1016/j.biopha.2019.108744

[376] Hsu, Y. P. et al. Instrument-free detection of FXYD3 using vial-based immunosensor for earlier and faster urothelial carcinoma diagnosis. *ACS Sens.* **5**, 928–935 (2020). 32162907 10.1021/acssensors.9b02013

[377] Mori, K. et al. Prognostic value of preoperative blood-based biomarkers in upper tract urothelial carcinoma treated with nephroureterectomy: a systematic review and meta-analysis. *Urol. Oncol.* **38**, 315–333 (2020). 32088103 10.1016/j.urolonc.2020.01.015

[378] Traeger, L. et al. Serum Hepcidin and GDF-15 levels as prognostic markers in urothelial carcinoma of the upper urinary tract and renal cell carcinoma. *BMC Cancer* **19**, 74 (2019). 30646851 10.1186/s12885-019-5278-0 PMC6334404

[379] Rogers, A. et al. Relative increase in leukemia-specific DNA in peripheral blood plasma from patients with acute myeloid leukemia and myelodysplasia. *Blood* **103**, 2799–2801 (2004). 14576069 10.1182/blood-2003-06-1840

[380] Zhao, P. et al. Using circulating tumor DNA to monitor myelodysplastic syndromes status. *Hematol. Oncol.* **37**, 531–533 (2019). 31291480 10.1002/hon.2649 PMC6900042

[381] Ruan, M. et al. Targeted next-generation sequencing of circulating tumor DNA, bone marrow, and peripheral blood mononuclear cells in pediatric AML. *Front. Oncol.* **11**, 666470 (2021). 34422630 10.3389/fonc.2021.666470 PMC8377768

[382] Garcia-Gisbert, N. et al. Molecular and cytogenetic characterization of myelodysplastic syndromes in cell-free DNA. *Blood Adv.* **6**, 3178–3188 (2022). 35192693 10.1182/bloodadvances.2021006565 PMC9131900

[383] Gao, Y. J. et al. Increased integrity of circulating cell-free DNA in plasma of patients with acute leukemia. *Clin. Chem. Lab Med.* **48**, 1651–1656 (2010). 20831457 10.1515/CCLM.2010.311

[384] Božic, T. et al. Investigation of measurable residual disease in acute myeloid leukemia by DNA methylation patterns. *Leukemia* **36**, 80–89 (2022). 34131280 10.1038/s41375-021-01316-z PMC8727289

[385] Suzuki, Y. et al. Peripheral blood cell-free DNA is an alternative tumor DNA source reflecting disease status in myelodysplastic syndromes. *Cancer Sci.* **107**, 1329–1337 (2016). 27323954 10.1111/cas.12994 PMC5021037

[386] Yao, C. Y. et al. Distinct mutation profile and prognostic relevance in patients with hypoplastic myelodysplastic syndromes (h-MDS). *Oncotarget* **7**, 63177–63188 (2016). 27527853 10.18632/oncotarget.11050 PMC5325355

[387] Liu, L. P. et al. Early detection of molecular residual disease and risk stratification for children with acute myeloid leukemia via circulating tumor DNA. *Clin. Cancer Res.* **30**, 1143–1151 (2024). 38170574 10.1158/1078-0432.CCR-23-2589

[388] Xue, Y. et al. Applications of circulating tumor DNA in myelodysplastic syndromes and acute myeloid leukemia: promises and challenges. *Front. Biosci. (Landmark Ed.).* **29**, 86 (2024). 38420833 10.31083/j.fbl2902086

[389] Nakamura, S. et al. Prognostic impact of circulating tumor DNA status post-allogeneic hematopoietic stem cell transplantation in AML and MDS. *Blood* **133**, 2682–2695 (2019). 30936070 10.1182/blood-2018-10-880690

[390] Rossi, D. et al. Diffuse large B-cell lymphoma genotyping on the liquid biopsy. *Blood* **129**, 1947–1957 (2017). 28096087 10.1182/blood-2016-05-719641

[391] Scherer, F. et al. Distinct biological subtypes and patterns of genome evolution in lymphoma revealed by circulating tumor DNA. *Sci. Transl. Med.* **8**, 364ra155 (2016). 27831904 10.1126/scitranslmed.aai8545 PMC5490494

[392] Roschewski, M. et al. Circulating tumour DNA and CT monitoring in patients with untreated diffuse large B-cell lymphoma: a correlative biomarker study. *Lancet Oncol.* **16**, 541–549 (2015). 25842160 10.1016/S1470-2045(15)70106-3 PMC4460610

[393] Lauer, E. M., Mutter, J. & Scherer, F. Circulating tumor DNA in B-cell lymphoma: technical advances, clinical applications, and perspectives for translational research. *Leukemia* **36**, 2151–2164 (2022). 35701522 10.1038/s41375-022-01618-w PMC9417989

[394] Kurtz, D. M. et al. Dynamic risk profiling using serial tumor biomarkers for personalized outcome prediction. *Cell* **178**, 699–713.e619 (2019). 31280963 10.1016/j.cell.2019.06.011 PMC7380118

[395] Kurtz, D. M. et al. Enhanced detection of minimal residual disease by targeted sequencing of phased variants in circulating tumor DNA. *Nat. Biotechnol.* **39**, 1537–1547 (2021). 34294911 10.1038/s41587-021-00981-w PMC8678141

[396] Oki, Y. et al. Detection of classical Hodgkin lymphoma specific sequence in peripheral blood using a next-generation sequencing approach. *Br. J. Haematol.* **169**, 689–693 (2015). 25818067 10.1111/bjh.13349 PMC5279064

[397] Desch, A. K. et al. Genotyping circulating tumor DNA of pediatric Hodgkin lymphoma. *Leukemia* **34**, 151–166 (2020). 31431735 10.1038/s41375-019-0541-6

[398] Roschewski, M., Rossi, D., Kurtz, D. M., Alizadeh, A. A. & Wilson, W. H. Circulating tumor DNA in lymphoma: principles and future directions. *Blood Cancer Discov.* **3**, 5–15 (2022). 35015693 10.1158/2643-3230.BCD-21-0029 PMC9245363

[399] Spina, V. et al. Circulating tumor DNA reveals genetics, clonal evolution, and residual disease in classical Hodgkin lymphoma. *Blood* **131**, 2413–2425 (2018). 29449275 10.1182/blood-2017-11-812073

[400] Mutter, J. A. et al. Circulating tumor DNA profiling for detection, risk stratification, and classification of brain lymphomas. *J. Clin. Oncol.* **41**, 1684–1694 (2023). 36542815 10.1200/JCO.22.00826 PMC10419411

[401] Roemer, M. G. M. et al. Major histocompatibility complex class II and programmed death ligand 1 expression predict outcome after programmed death 1 blockade in classic Hodgkin lymphoma. *J. Clin. Oncol.* **36**, 942–950 (2018). 29394125 10.1200/JCO.2017.77.3994 PMC5877802

[402] Li, S., Zhang, E. & Cai, Z. Liquid biopsy by analysis of circulating myeloma cells and cell-free nucleic acids: a novel noninvasive approach of disease evaluation in multiple myeloma. *Biomark. Res.* **11**, 27 (2023). 36890597 10.1186/s40364-023-00469-6 PMC9997021

[403] Garcés, J. J. et al. Circulating tumor cells for comprehensive and multiregional non-invasive genetic characterization of multiple myeloma. *Leukemia* **34**, 3007–3018 (2020). 32475991 10.1038/s41375-020-0883-0

[404] Mishima, Y. et al. The mutational landscape of circulating tumor cells in multiple myeloma. *Cell Rep.* **19**, 218–224 (2017). 28380360 10.1016/j.celrep.2017.03.025 PMC5439509

[405] Rustad, E. H. et al. Monitoring multiple myeloma by quantification of recurrent mutations in serum. *Haematologica* **102**, 1266–1272 (2017). 28385781 10.3324/haematol.2016.160564 PMC5566041

[406] Mithraprabhu, S. et al. Circulating tumour DNA analysis demonstrates spatial mutational heterogeneity that coincides with disease relapse in myeloma. *Leukemia* **31**, 1695–1705 (2017). 27899805 10.1038/leu.2016.366

[407] Mithraprabhu, S., Sirdesai, S., Chen, M., Khong, T. & Spencer, A. Circulating tumour DNA analysis for tumour genome characterisation and monitoring disease burden in extramedullary multiple myeloma. *Int J. Mol. Sci.* **19**, 1858 (2018). 29937522 10.3390/ijms19071858 PMC6073672

[408] Gerber, B. et al. Circulating tumor DNA as a liquid biopsy in plasma cell dyscrasias. *Haematologica* **103**, e245–e248 (2018). 29472358 10.3324/haematol.2017.184358 PMC6058782

[409] Coffey, D. G. et al. High-throughput drug screening and multi-omic analysis to guide individualized treatment for multiple myeloma. *JCO Precis. Oncol.* **5**, PO.20.00442 (2021). 34250400 10.1200/PO.20.00442 PMC8232547

[410] Li, S. et al. Targeting the GCK pathway: a novel and selective therapeutic strategy against RAS-mutated multiple myeloma. *Blood* **137**, 1754–1764 (2021). 33036022 10.1182/blood.2020006334 PMC8020269

[411] Giesen, N. et al. A phase 2 clinical trial of combined BRAF/MEK inhibition for BRAFV600E-mutated multiple myeloma. *Blood* **141**, 1685–1690 (2023). 36608320 10.1182/blood.2022017789

[412] Brown, R. L., de Souza, J. A. & Cohen, E. E. Thyroid cancer: burden of illness and management of disease. *J. Cancer* **2**, 193–199 (2011). 21509149 10.7150/jca.2.193 PMC3079916

[413] Kure, S. & Ohashi, R. Thyroid Hürthle cell carcinoma: clinical, pathological, and molecular features. *Cancers* **13**, 26 (2020). 33374707 10.3390/cancers13010026 PMC7793513

[414] Schlumberger, M. & Leboulleux, S. Current practice in patients with differentiated thyroid cancer. *Nat. Rev. Endocrinol.* **17**, 176–188 (2021). 33339988 10.1038/s41574-020-00448-z

[415] Bankó, P. et al. Technologies for circulating tumor cell separation from whole blood. *J. Hematol. Oncol.* **12**, 48 (2019). 31088479 10.1186/s13045-019-0735-4 PMC6518774

[416] Feng, Z. et al. Circulating tumor cells in the early detection of human cancers. *Int. J. Biol. Sci.* **18**, 3251–3265 (2022). 35637960 10.7150/ijbs.71768 PMC9134923

[417] Ehlers, M. et al. Increased numbers of circulating tumor cells in thyroid cancer patients. *Horm. Metab. Res.* **50**, 602–608 (2018). 30081408 10.1055/a-0651-4913

[418] Zane, M. et al. Circulating cell-free DNA, SLC5A8 and SLC26A4 hypermethylation, BRAF(V600E): a non-invasive tool panel for early detection of thyroid cancer. *Biomed. Pharmacother.* **67**, 723–730 (2013). 23931930 10.1016/j.biopha.2013.06.007

[419] Liu, Y., Geng, H., Liu, X., Cao, M. & Zhang, X. A meta-analysis of circulating microRNAs in the diagnosis of papillary thyroid carcinoma. *PLoS One* **16**, e0251676 (2021). 34019567 10.1371/journal.pone.0251676 PMC8139519

[420] Delcorte, O. et al. Two miRNAs enriched in plasma extracellular vesicles are potential biomarkers for thyroid cancer. *Endocr. Relat. Cancer* **29**, 389–401 (2022). 35521768 10.1530/ERC-21-0343

[421] Sato, T. et al. Circulating tumor cells detected by reverse transcription-polymerase chain reaction for carcinoembryonic antigen mRNA: distinguishing follicular thyroid carcinoma from adenoma. *Surgery* **137**, 552–558 (2005). 15855928 10.1016/j.surg.2004.11.006

[422] Qiu, Z. L. et al. Circulating tumor cells correlate with clinicopathological features and outcomes in differentiated thyroid cancer. *Cell Physiol. Biochem.* **48**, 718–730 (2018). 30025398 10.1159/000491898

[423] Yan, C., Huang, M., Li, X., Wang, T. & Ling, R. Relationship between BRAF V600E and clinical features in papillary thyroid carcinoma. *Endocr. Connect.* **8**, 988–996 (2019). 31252408 10.1530/EC-19-0246 PMC6652244

[424] Allin, D. M. et al. Circulating tumour DNA is a potential biomarker for disease progression and response to targeted therapy in advanced thyroid cancer. *Eur. J. Cancer* **103**, 165–175 (2018). 30253333 10.1016/j.ejca.2018.08.013

[425] Ciampi, R. et al. Pre- and post-operative circulating tumoral DNA in patients with medullary thyroid carcinoma. *J. Clin. Endocrinol. Metab.* **107**, e3420–e3427 (2022). 35470851 10.1210/clinem/dgac222

[426] Lubitz, C. C. et al. Circulating BRAF(V600E) levels correlate with treatment in patients with thyroid carcinoma. *Thyroid* **28**, 328–339 (2018). 29378474 10.1089/thy.2017.0322 PMC5865613

[427] Cote, G. J. et al. Prognostic significance of circulating RET M918T mutated tumor DNA in patients with advanced medullary thyroid carcinoma. *J. Clin. Endocrinol. Metab.* **102**, 3591–3599 (2017). 28911154 10.1210/jc.2017-01039 PMC5587058

[428] Cradic, K. W. et al. Mutant BRAF(T1799A) can be detected in the blood of papillary thyroid carcinoma patients and correlates with disease status. *J. Clin. Endocrinol. Metab.* **94**, 5001–5009 (2009). 19850689 10.1210/jc.2009-1349

[429] Qin, Y. et al. Clinical utility of circulating cell-free DNA mutations in anaplastic thyroid carcinoma. *Thyroid* **31**, 1235–1243 (2021). 33599171 10.1089/thy.2020.0296 PMC8420950

[430] Hu, S. et al. Detection of serum deoxyribonucleic acid methylation markers: a novel diagnostic tool for thyroid cancer. *J. Clin. Endocrinol. Metab.* **91**, 98–104 (2006). 16263813 10.1210/jc.2005-1810

[431] Wen, Q., Wang, Y., Li, X., Jin, X. & Wang, G. Decreased serum exosomal miR-29a expression and its clinical significance in papillary thyroid carcinoma. *J. Clin. Lab. Anal.* **35**, e23560 (2021). 33368640 10.1002/jcla.23560 PMC7843262

[432] Lee, J. C. et al. Papillary thyroid cancer-derived exosomes contain miRNA-146b and miRNA-222. *J. Surg. Res.* **196**, 39–48 (2015). 25819770 10.1016/j.jss.2015.02.027

[433] Toraih, E. A. et al. Diagnostic and prognostic performance of liquid biopsy-derived exosomal microRNAs in thyroid cancer patients: a systematic review and meta-analysis. *Cancers* **13**, 4295 (2021). 34503104 10.3390/cancers13174295 PMC8428356

[434] Chen, W. et al. Cancer statistics in China, 2015. *CA Cancer J. Clin.* **66**, 115–132 (2016). 26808342 10.3322/caac.21338

[435] Freitas, A. J. A. et al. Liquid Biopsy as a Tool for the Diagnosis, Treatment, and Monitoring of Breast Cancer. *Int. J. Mol. Sci.* **23**, 9952 (2022). 36077348 10.3390/ijms23179952 PMC9456236

[436] Guttery, D. S. et al. Noninvasive detection of activating estrogen receptor 1 (ESR1) mutations in estrogen receptor-positive metastatic breast cancer. *Clin. Chem.* **61**, 974–982 (2015). 25979954 10.1373/clinchem.2015.238717

[437] Garcia-Murillas, I. et al. Mutation tracking in circulating tumor DNA predicts relapse in early breast cancer. *Sci. Transl. Med.* **7**, 302ra133 (2015). 26311728 10.1126/scitranslmed.aab0021

[438] Beaver, J. A. et al. Detection of cancer DNA in plasma of patients with early-stage breast cancer. *Clin. Cancer Res.* **20**, 2643–2650 (2014). 24504125 10.1158/1078-0432.CCR-13-2933 PMC4024333

[439] Rugo, H. S. et al. Alpelisib plus fulvestrant in PIK3CA-mutated, hormone receptor-positive advanced breast cancer after a CDK4/6 inhibitor (BYLieve): one cohort of a phase 2, multicentre, open-label, non-comparative study. *Lancet Oncol.* **22**, 489–498 (2021). 33794206 10.1016/S1470-2045(21)00034-6

[440] Hai, L., Li, L., Liu, Z., Tong, Z. & Sun, Y. Whole-genome circulating tumor DNA methylation landscape reveals sensitive biomarkers of breast cancer. *MedComm* **3**, e134 (2022). 35756163 10.1002/mco2.134 PMC9205580

[441] Hannafon, B. N. et al. Plasma exosome microRNAs are indicative of breast cancer. *Breast Cancer Res.* **18**, 90 (2016). 27608715 10.1186/s13058-016-0753-x PMC5016889

[442] Eichelser, C. et al. Increased serum levels of circulating exosomal microRNA-373 in receptor-negative breast cancer patients. *Oncotarget* **5**, 9650–9663 (2014). 25333260 10.18632/oncotarget.2520 PMC4259427

[443] Li, M. et al. Circulating microRNAs from the miR-106a-363 cluster on chromosome X as novel diagnostic biomarkers for breast cancer. *Breast Cancer Res. Treat.* **170**, 257–270 (2018). 29557526 10.1007/s10549-018-4757-3 PMC5999170

[444] Liu, C. et al. Single-exosome-counting immunoassays for cancer diagnostics. *Nano Lett.* **18**, 4226–4232 (2018). 29888919 10.1021/acs.nanolett.8b01184

[445] Racila, E. et al. Detection and characterization of carcinoma cells in the blood. *Proc. Natl Acad. Sci. USA* **95**, 4589–4594 (1998). 9539782 10.1073/pnas.95.8.4589 PMC22534

[446] Ozkumur, E. et al. Inertial focusing for tumor antigen-dependent and -independent sorting of rare circulating tumor cells. *Sci. Transl. Med.* **5**, 179ra147 (2013). 10.1126/scitranslmed.3005616 PMC3760275 23552373

[447] Hvichia, G. E. et al. A novel microfluidic platform for size and deformability based separation and the subsequent molecular characterization of viable circulating tumor cells. *Int. J. Cancer* **138**, 2894–2904 (2016). 26789903 10.1002/ijc.30007 PMC5069649

[448] Allard, W. J. et al. Tumor cells circulate in the peripheral blood of all major carcinomas but not in healthy subjects or patients with nonmalignant diseases. *Clin. Cancer Res.* **10**, 6897–6904 (2004). 15501967 10.1158/1078-0432.CCR-04-0378

[449] Gradishar, W. J. et al. Breast cancer, version 3.2022, NCCN clinical practice guidelines in oncology. *J. Natl Compr. Cancer Netw.* **20**, 691–722 (2022). 10.6004/jnccn.2022.0030 35714673

[450] Dawson, S. J. et al. Analysis of circulating tumor DNA to monitor metastatic breast cancer. *N. Engl. J. Med.* **368**, 1199–1209 (2013). 23484797 10.1056/NEJMoa1213261

[451] Famta, P. et al. Enigmatic role of exosomes in breast cancer progression and therapy. *Life Sci.* **289**, 120210 (2022). 34875250 10.1016/j.lfs.2021.120210

[452] Nakamura, S. et al. Multi-center study evaluating circulating tumor cells as a surrogate for response to treatment and overall survival in metastatic breast cancer. *Breast Cancer* **17**, 199–204 (2010). 19649686 10.1007/s12282-009-0139-3

[453] Jiang, Z. et al. Chinese Society of Clinical Oncology (CSCO) breast cancer guidelines 2022. *Transl. Breast Cancer Res.* **3**, 13 (2022). 38751537 10.21037/tbcr-22-21 PMC11093004

[454] Cristiano, S. et al. Genome-wide cell-free DNA fragmentation in patients with cancer. *Nature* **570**, 385–389 (2019). 31142840 10.1038/s41586-019-1272-6 PMC6774252

[455] Klinge, C. M. Non-coding RNAs in breast cancer: intracellular and intercellular communication. *Noncoding RNA* **4**, 40 (2018). 30545127 10.3390/ncrna4040040 PMC6316884

[456] Tierno, D., Grassi, G., Zanconati, F., Dapas, B. & Scaggiante, B. Plasma circular RNAs as biomarkers for breast cancer. *Biomedicines* **12**, 875 (2024). 38672229 10.3390/biomedicines12040875 PMC11048241

[457] Benini, S. et al. Detection of circulating tumor cells in liquid biopsy from Ewing sarcoma patients. *Cancer Manag Res.* **10**, 49–60 (2018). 29386915 10.2147/CMAR.S141623 PMC5765973

[458] Krumbholz, M. et al. Genomic EWSR1 fusion sequence as highly sensitive and dynamic plasma tumor marker in Ewing sarcoma. *Clin. Cancer Res.* **22**, 4356–4365 (2016). 27283964 10.1158/1078-0432.CCR-15-3028

[459] Gutteridge, A. et al. Digital PCR analysis of circulating tumor DNA: a biomarker for chondrosarcoma diagnosis, prognostication, and residual disease detection. *Cancer Med.* **6**, 2194–2202 (2017). 28834325 10.1002/cam4.1146 PMC5633548

[460] Klega, K. et al. Detection of somatic structural variants enables quantification and characterization of circulating tumor DNA in children With solid tumors. *JCO Precis. Oncol.* **2018**, PO.17.00285 (2018). 30027144 10.1200/PO.17.00285 PMC6049092

[461] McBride, D. J. et al. Use of cancer-specific genomic rearrangements to quantify disease burden in plasma from patients with solid tumors. *Genes Chromosomes Cancer* **49**, 1062–1069 (2010). 20725990 10.1002/gcc.20815 PMC3145117

[462] Shulman, D. S. et al. Detection of circulating tumour DNA is associated with inferior outcomes in Ewing sarcoma and osteosarcoma: a report from the Children’s Oncology Group. *Br. J. Cancer* **119**, 615–621 (2018). 30131550 10.1038/s41416-018-0212-9 PMC6162271

[463] Momen-Heravi, F. et al. Current methods for the isolation of extracellular vesicles. *Biol. Chem.* **394**, 1253–1262 (2013). 23770532 10.1515/hsz-2013-0141 PMC7075462

[464] Fang, S. et al. Clinical application of a microfluidic chip for immunocapture and quantification of circulating exosomes to assist breast cancer diagnosis and molecular classification. *PLoS One* **12**, e0175050 (2017). 28369094 10.1371/journal.pone.0175050 PMC5378374

[465] Liang, L. G. et al. An integrated double-filtration microfluidic device for isolation, enrichment and quantification of urinary extracellular vesicles for detection of bladder cancer. *Sci. Rep.* **7**, 46224 (2017). 28436447 10.1038/srep46224 PMC5402302

[466] Vaidyanathan, R. et al. Detecting exosomes specifically: a multiplexed device based on alternating current electrohydrodynamic induced nanoshearing. *Anal. Chem.* **86**, 11125–11132 (2014). 25324037 10.1021/ac502082b

[467] Sina, A. A. et al. Real time and label free profiling of clinically relevant exosomes. *Sci. Rep.* **6**, 30460 (2016). 27464736 10.1038/srep30460 PMC4964344

[468] Gholizadeh, S. et al. Microfluidic approaches for isolation, detection, and characterization of extracellular vesicles: current status and future directions. *Biosens. Bioelectron.* **91**, 588–605 (2017). 28088752 10.1016/j.bios.2016.12.062 PMC5323331

[469] Liga, A., Vliegenthart, A. D., Oosthuyzen, W., Dear, J. W. & Kersaudy-Kerhoas, M. Exosome isolation: a microfluidic road-map. *Lab. Chip* **15**, 2388–2394 (2015). 25940789 10.1039/c5lc00240k

[470] Nugent, M. MicroRNA function and dysregulation in bone tumors: the evidence to date. *Cancer Manag. Res.* **6**, 15–25 (2014). 24426787 10.2147/CMAR.S53928 PMC3890404

[471] Lulla, R. R. et al. Identification of differentially expressed MicroRNAs in osteosarcoma. *Sarcoma* **2011**, 732690 (2011). 21789031 10.1155/2011/732690 PMC3140035

[472] Tian, Q. et al. A causal role for circulating miR-34b in osteosarcoma. *Eur. J. Surg. Oncol.* **40**, 67–72 (2014). 24063968 10.1016/j.ejso.2013.08.024

[473] Urdinez, J. et al. The miR-143/145 cluster, a novel diagnostic biomarker in chondrosarcoma, acts as a tumor suppressor and directly inhibits Fascin-1. *J. Bone Min. Res.* **35**, 1077–1091 (2020). 10.1002/jbmr.3976 32027760

[474] Parafioriti, A. et al. Expression profiling of microRNAs and isomiRs in conventional central chondrosarcoma. *Cell Death Discov.* **6**, 46 (2020). 32566253 10.1038/s41420-020-0282-3 PMC7287106

[475] Sciandra, M. et al. Circulating miR34a levels as a potential biomarker in the follow-up of Ewing sarcoma. *J. Cell Commun. Signal* **14**, 335–347 (2020). 32504411 10.1007/s12079-020-00567-2 PMC7511499

[476] Zhang, S., Li, D., Jiao, G. J., Wang, H. L. & Yan, T. B. miR-185 suppresses progression of Ewing’s sarcoma via inhibiting the PI3K/AKT and Wnt/β-catenin pathways. *Onco Targets Ther.* **11**, 7967–7977 (2018). 30519038 10.2147/OTT.S167771 PMC6235341

[477] Cafforio, P. et al. Liquid biopsy in cervical cancer: hopes and pitfalls. *Cancers* **13**, 3968 (2021). 34439120 10.3390/cancers13163968 PMC8394398

[478] Thangarajah, F. et al. Digital droplet PCR-based quantification of ccfHPV-DNA as liquid biopsy in HPV-driven cervical and vulvar cancer. *J. Cancer Res. Clin. Oncol.* **149**, 12597–12604 (2023). 37452202 10.1007/s00432-023-05077-3 PMC10587338

[479] Charo, L. M. et al. Clinical implications of plasma circulating tumor DNA in gynecologic cancer patients. *Mol. Oncol.* **15**, 67–79 (2021). 32881280 10.1002/1878-0261.12791 PMC7782073

[480] Galati, L. et al. Detection of circulating HPV16 DNA as a biomarker for cervical cancer by a bead-based HPV genotyping assay. *Microbiol. Spectr.* **10**, e0148021 (2022). 35225653 10.1128/spectrum.01480-21 PMC9045285

[481] Tornesello, M. L. et al. The role of microRNAs, long non-coding RNAs, and circular RNAs in cervical cancer. *Front. Oncol.* **10**, 150 (2020). 32154165 10.3389/fonc.2020.00150 PMC7044410

[482] Sun, W. et al. Four circulating long non-coding RNAs act as biomarkers for predicting cervical cancer. *Gynecol. Obstet. Investig.* **83**, 533–539 (2018). 30134241 10.1159/000487595

[483] Jia, W. et al. Expression profile of circulating microRNAs as a promising fingerprint for cervical cancer diagnosis and monitoring. *Mol. Clin. Oncol.* **3**, 851–858 (2015). 26171195 10.3892/mco.2015.560 PMC4486870

[484] Liu, P., Xin, F. & Ma, C. F. Clinical significance of serum miR-196a in cervical intraepithelial neoplasia and cervical cancer. *Genet. Mol. Res.* **14**, 17995–18002 (2015). 26782446 10.4238/2015.December.22.25

[485] Sun, L. et al. MicoRNA-425-5p is a potential prognostic biomarker for cervical cancer. *Ann. Clin. Biochem.* **54**, 127–133 (2017). 27166306 10.1177/0004563216649377

[486] Page, K. et al. Next generation sequencing of circulating cell-free DNA for evaluating mutations and gene amplification in metastatic breast cancer. *Clin. Chem.* **63**, 532–541 (2017). 27940449 10.1373/clinchem.2016.261834 PMC6241835

[487] Bohers, E. et al. Somatic mutations of cell-free circulating DNA detected by next-generation sequencing reflect the genetic changes in both germinal center B-cell-like and activated B-cell-like diffuse large B-cell lymphomas at the time of diagnosis. *Haematologica* **100**, e280–e284 (2015). 25749829 10.3324/haematol.2015.123612 PMC4486242

[488] Chicard, M. et al. Whole-exome sequencing of cell-free DNA reveals temporo-spatial heterogeneity and identifies treatment-resistant clones in neuroblastoma. *Clin. Cancer Res.* **24**, 939–949 (2018). 29191970 10.1158/1078-0432.CCR-17-1586

[489] Tian, X. et al. Dynamic analysis of circulating tumor DNA to predict prognosis and monitor therapeutic response in metastatic relapsed cervical cancer. *Int. J. Cancer* **148**, 921–931 (2021). 33113150 10.1002/ijc.33362

[490] Tewari, K. S. et al. Circulating Tumor Cells In Advanced Cervical Cancer: NRG Oncology-Gynecologic Oncology Group Study 240 (NCT 00803062). *Mol. Cancer Ther.* **19**, 2363–2370 (2020). 32847980 10.1158/1535-7163.MCT-20-0276 PMC7907274

[491] Weismann, P. et al. The detection of circulating tumor cells expressing E6/E7 HR-HPV oncogenes in peripheral blood in cervical cancer patients after radical hysterectomy. *Neoplasma* **56**, 230–238 (2009). 19309226 10.4149/neo_2009_03_230

[492] Obermayr, E. et al. Assessment of a six gene panel for the molecular detection of circulating tumor cells in the blood of female cancer patients. *BMC Cancer* **10**, 666 (2010). 21129172 10.1186/1471-2407-10-666 PMC3013085

[493] Kiss, I., Kolostova, K., Pawlak, I. & Bobek, V. Circulating tumor cells in gynaecological malignancies. *J. Buon* **25**, 40–50 (2020). 32277613

[494] Du, K. et al. Circulating tumor cells counting act as a potential prognostic factor in cervical cancer. *Technol. Cancer Res. Treat.* **19**, 1533033820957005 (2020). 33034270 10.1177/1533033820957005 PMC7549154

[495] Constantine, G. D., Kessler, G., Graham, S. & Goldstein, S. R. Increased incidence of endometrial cancer following the women’s health initiative: an assessment of risk factors. *J. Women’s. Health* **28**, 237–243 (2019). 10.1089/jwh.2018.6956 PMC6390656 30484734

[496] Lortet-Tieulent, J., Ferlay, J., Bray, F. & Jemal, A. International patterns and trends in endometrial cancer incidence, 1978-2013. *J. Natl. Cancer Inst.* **110**, 354–361 (2018). 29045681 10.1093/jnci/djx214

[497] Shen, Y., Shi, R., Zhao, R. & Wang, H. Clinical application of liquid biopsy in endometrial carcinoma. *Med. Oncol.* **40**, 92 (2023). 36757457 10.1007/s12032-023-01956-4 PMC9911505

[498] Kiss, I. et al. Correlation between disease stage and the presence of viable circulating tumor cells in endometrial cancer. *Anticancer Res.* **38**, 2983–2987 (2018). 29715128 10.21873/anticanres.12550

[499] Bogani, G. et al. Detection of circulating tumor cells in high-risk endometrial cancer. *Anticancer Res.* **35**, 683–687 (2015). 25667446

[500] Bolivar, A. M. et al. Targeted next-generation sequencing of endometrial cancer and matched circulating tumor DNA: identification of plasma-based, tumor-associated mutations in early-stage patients. *Mod. Pathol.* **32**, 405–414 (2019). 30315273 10.1038/s41379-018-0158-8 PMC6395490

[501] Wang, L. et al. Circulating microRNAs as a fingerprint for endometrial endometrioid adenocarcinoma. *PLoS One* **9**, e110767 (2014). 25329674 10.1371/journal.pone.0110767 PMC4203829

[502] Buscail, E. et al. Tumor-proximal liquid biopsy to improve diagnostic and prognostic performances of circulating tumor cells. *Mol. Oncol.* **13**, 1811–1826 (2019). 31216108 10.1002/1878-0261.12534 PMC6717761

[503] Grant, B. M., Pugh, T. J. & Oza, A. M. Molecular monitoring in endometrial cancer-ready for prime time? *Clin. Cancer Res.* **29**, 305–308 (2023). 36354753 10.1158/1078-0432.CCR-22-2781

[504] He, D. et al. DNMT3A/3B overexpression might be correlated with poor patient survival, hypermethylation and low expression of ESR1/PGR in endometrioid carcinoma: an analysis of The Cancer Genome Atlas. *Chin. Med J.* **132**, 161–170 (2019). 30614867 10.1097/CM9.0000000000000054 PMC6365298

[505] Yang, J. et al. Identification of Endometrial Cancer-Specific microRNA Biomarkers in Endometrial Fluid. *Int. J. Mol. Sci.* **24**, 8683 (2023). 37240034 10.3390/ijms24108683 PMC10218319

[506] Urabe, F. et al. Extracellular vesicles as biomarkers and therapeutic targets for cancer. *Am. J. Physiol. Cell Physiol.* **318**, C29–c39 (2020). 31693397 10.1152/ajpcell.00280.2019

[507] Nakamura, K. et al. Clinical relevance of circulating cell-free microRNAs in ovarian cancer. *Mol. Cancer* **15**, 48 (2016). 27343009 10.1186/s12943-016-0536-0 PMC4921011

[508] S, E. L. A., Mäger, I., Breakefield, X. O. & Wood, M. J. Extracellular vesicles: biology and emerging therapeutic opportunities. *Nat. Rev. Drug Discov.* **12**, 347–357 (2013). 23584393 10.1038/nrd3978

[509] van den Helder, R. et al. Non-invasive detection of endometrial cancer by DNA methylation analysis in urine. *Clin. Epigenet.* **12**, 165 (2020). 10.1186/s13148-020-00958-7 PMC7640380 33143739

[510] Karimi, F. et al. Liquid biopsy in ovarian cancer: advantages and limitations for prognosis and diagnosis. *Med. Oncol.* **40**, 265 (2023). 37561363 10.1007/s12032-023-02128-0

[511] Marth, C., Kisic, J., Kaern, J., Tropé, C. & Fodstad, Ø. Circulating tumor cells in the peripheral blood and bone marrow of patients with ovarian carcinoma do not predict prognosis. *Cancer* **94**, 707–712 (2002). 11857303 10.1002/cncr.10250

[512] Judson, P. L. et al. Preoperative detection of peripherally circulating cancer cells and its prognostic significance in ovarian cancer. *Gynecol. Oncol.* **91**, 389–394 (2003). 14599871 10.1016/j.ygyno.2003.08.004

[513] Zhu, J. W., Charkhchi, P. & Akbari, M. R. Potential clinical utility of liquid biopsies in ovarian cancer. *Mol. Cancer* **21**, 114 (2022). 35545786 10.1186/s12943-022-01588-8 PMC9092780

[514] Asante, D. B., Calapre, L., Ziman, M., Meniawy, T. M. & Gray, E. S. Liquid biopsy in ovarian cancer using circulating tumor DNA and cells: ready for prime time? *Cancer Lett.* **468**, 59–71 (2020). 31610267 10.1016/j.canlet.2019.10.014

[515] Siena, S. et al. Dynamic molecular analysis and clinical correlates of tumor evolution within a phase II trial of panitumumab-based therapy in metastatic colorectal cancer. *Ann. Oncol.* **29**, 119–126 (2018). 28945848 10.1093/annonc/mdx504 PMC5834114

[516] Wan, J. C. M. et al. Liquid biopsies come of age: towards implementation of circulating tumour DNA. *Nat. Rev. Cancer* **17**, 223–238 (2017). 28233803 10.1038/nrc.2017.7

[517] Zheng, X., Li, X. & Wang, X. Extracellular vesicle-based liquid biopsy holds great promise for the management of ovarian cancer. *Biochim. Biophys. Acta Rev. Cancer* **1874**, 188395 (2020). 32698041 10.1016/j.bbcan.2020.188395

[518] Wang, L., Zhao, F., Xiao, Z. & Yao, L. Exosomal microRNA-205 is involved in proliferation, migration, invasion, and apoptosis of ovarian cancer cells via regulating VEGFA. *Cancer Cell Int.* **19**, 281 (2019). 31719795 10.1186/s12935-019-0990-z PMC6836480

[519] Mateescu, B. et al. miR-141 and miR-200a act on ovarian tumorigenesis by controlling oxidative stress response. *Nat. Med.* **17**, 1627–1635 (2011). 22101765 10.1038/nm.2512

[520] Konstantinopoulos, P. A., Lheureux, S. & Moore, K. N. PARP inhibitors for ovarian cancer: current indications, future combinations, and novel assets in development to target DNA damage repair. *Am. Soc. Clin. Oncol. Educ. Book* **40**, 1–16 (2020). 32364757 10.1200/EDBK_288015

[521] Wang, M. et al. Circular RNAs: a novel type of non-coding RNA and their potential implications in antiviral immunity. *Int. J. Biol. Sci.* **13**, 1497–1506 (2017). 29230098 10.7150/ijbs.22531 PMC5723916

[522] Zhou, W. et al. Serum exosomes from epithelial ovarian cancer patients contain LRP1, which promotes the migration of epithelial ovarian cancer cell. *Mol. Cell Proteom.* **22**, 100520 (2023). 10.1016/j.mcpro.2023.100520 PMC10113894 36842607

[523] Li, J. et al. Claudin-containing exosomes in the peripheral circulation of women with ovarian cancer. *BMC Cancer* **9**, 244 (2009). 19619303 10.1186/1471-2407-9-244 PMC2719664

[524] Su, Y. Y. et al. Upregulated expression of serum exosomal miR-375 and miR-1307 enhance the diagnostic power of CA125 for ovarian cancer. *J. Ovar. Res.* **12**, 6 (2019). 10.1186/s13048-018-0477-x PMC6341583 30670062

[525] Yazawa, H. et al. Hydrodynamics-based gene delivery of naked DNA encoding fetal liver kinase-1 gene effectively suppresses the growth of pre-existing tumors. *Cancer Gene Ther.* **13**, 993–1001 (2006). 16763608 10.1038/sj.cgt.7700970

[526] Siegel, R. L., Miller, K. D., Wagle, N. S. & Jemal, A. Cancer statistics, 2023. *CA Cancer J. Clin.* **73**, 17–48 (2023). 36633525 10.3322/caac.21763

[527] Lilja, H., Ulmert, D. & Vickers, A. J. Prostate-specific antigen and prostate cancer: prediction, detection and monitoring. *Nat. Rev. Cancer* **8**, 268–278 (2008). 18337732 10.1038/nrc2351

[528] Sharma, S. et al. Circulating tumor cell isolation, culture, and downstream molecular analysis. *Biotechnol. Adv.* **36**, 1063–1078 (2018). 29559380 10.1016/j.biotechadv.2018.03.007 PMC5971144

[529] Schaeffer, E. M. et al. Prostate cancer, version 4.2023, NCCN clinical practice guidelines in oncology. *J. Natl Compr. Cancer Netw.* **21**, 1067–1096 (2023). 10.6004/jnccn.2023.0050 37856213

[530] Lindsay, C. R. et al. Vimentin and Ki67 expression in circulating tumour cells derived from castrate-resistant prostate cancer. *BMC Cancer* **16**, 168 (2016). 26923772 10.1186/s12885-016-2192-6 PMC4770547

[531] Schütz, E. et al. Chromosomal instability in cell-free DNA is a serum biomarker for prostate cancer. *Clin. Chem.* **61**, 239–248 (2015). 25348670 10.1373/clinchem.2014.226571

[532] Choudhury, A. D. et al. Tumor fraction in cell-free DNA as a biomarker in prostate cancer. *JCI Insight* **3**, e122109 (2018). 30385733 10.1172/jci.insight.122109 PMC6238737

[533] Han, X. Y. et al. A new mycobacterium species causing diffuse lepromatous leprosy. *Am. J. Clin. Pathol.* **130**, 856–864 (2008). 19019760 10.1309/AJCPP72FJZZRRVMM

[534] Singh, N. et al. The long noncoding RNA H19 regulates tumor plasticity in neuroendocrine prostate cancer. *Nat. Commun.* **12**, 7349 (2021). 34934057 10.1038/s41467-021-26901-9 PMC8692330

[535] O’Brien, J., Hayder, H., Zayed, Y. & Peng, C. Overview of MicroRNA biogenesis, mechanisms of actions, and circulation. *Front. Endocrinol.* **9**, 402 (2018). 10.3389/fendo.2018.00402 PMC6085463 30123182

[536] Sharova, E. et al. A circulating miRNA assay as a first-line test for prostate cancer screening. *Br. J. Cancer* **114**, 1362–1366 (2016). 27228285 10.1038/bjc.2016.151 PMC4984473

[537] Matsuzaki, K. et al. MiR-30b-3p and miR-126-3p of urinary extracellular vesicles could be new biomarkers for prostate cancer. *Transl. Androl. Urol.* **10**, 1918–1927 (2021). 33968679 10.21037/tau-20-421 PMC8100845

[538] Zhang, H. L. et al. Serum miRNA-21: elevated levels in patients with metastatic hormone-refractory prostate cancer and potential predictive factor for the efficacy of docetaxel-based chemotherapy. *Prostate* **71**, 326–331 (2011). 20842666 10.1002/pros.21246

[539] Selth, L. A. et al. Circulating microRNAs predict biochemical recurrence in prostate cancer patients. *Br. J. Cancer* **109**, 641–650 (2013). 23846169 10.1038/bjc.2013.369 PMC3738112

[540] Liang, C. et al. Long non-coding RNA PCAT-1 in human cancers: a meta-analysis. *Clin. Chim. Acta* **480**, 47–55 (2018). 29378170 10.1016/j.cca.2018.01.043

[541] Xue, Y. et al. Association between lncrna PCGEM1 polymorphisms and prostate cancer risk. *Prostate Cancer Prostatic Dis.* **16**, 139–144 (2013). 23459097 10.1038/pcan.2013.6

[542] Prensner, J. R. et al. RNA biomarkers associated with metastatic progression in prostate cancer: a multi-institutional high-throughput analysis of SChLAP1. *Lancet Oncol.* **15**, 1469–1480 (2014). 25456366 10.1016/S1470-2045(14)71113-1 PMC4559342

[543] Prensner, J. R. et al. The long noncoding RNA SChLAP1 promotes aggressive prostate cancer and antagonizes the SWI/SNF complex. *Nat. Genet.* **45**, 1392–1398 (2013). 24076601 10.1038/ng.2771 PMC3812362

[544] Logozzi, M. et al. Increased PSA expression on prostate cancer exosomes in in vitro condition and in cancer patients. *Cancer Lett.* **403**, 318–329 (2017). 28694142 10.1016/j.canlet.2017.06.036

[545] De Giorgi, U., Conteduca, V., Scarpi, E. & Re: Marzia Del Re, Elisa Biasco, Stefania Crucitta, et al. The detection of androgen receptor splice variant 7 in plasma-derived exosomal rna strongly predicts resistance to hormonal therapy in metastatic prostate cancer patients. Eur Urol 2017;71:680-7. *Eur. Urol.* **73**, e9–e10 (2018). 28789806 10.1016/j.eururo.2017.07.032

[546] Nimir, M. et al. Detection of AR-V7 in liquid biopsies of castrate resistant prostate cancer patients: a comparison of AR-V7 analysis in circulating tumor cells, circulating tumor RNA and exosomes. *Cells* **8**, 688 (2019). 31288377 10.3390/cells8070688 PMC6678978

[547] Raos, D. et al. cfDNA methylation in liquid biopsies as potential testicular seminoma biomarker. *Epigenomics* **14**, 1493–1507 (2022). 36722130 10.2217/epi-2022-0331

[548] Wang, K., Wang, X., Pan, Q. & Zhao, B. Liquid biopsy techniques and pancreatic cancer: diagnosis, monitoring, and evaluation. *Mol. Cancer* **22**, 167 (2023). 37803304 10.1186/s12943-023-01870-3 PMC10557192

[549] Murray, M. J. et al. Solid tumors of childhood display specific serum microRNA profiles. *Cancer Epidemiol. Biomark. Prev.* **24**, 350–360 (2015). 10.1158/1055-9965.EPI-14-0669 PMC4340540 25416717

[550] Klein, A., Fishman, A., Zemer, R., Zimlichman, S. & Altaras, M. M. Detection of tumor circulating cells by cytokeratin 20 in the blood of patients with endometrial carcinoma. *Gynecol. Oncol.* **78**, 352–355 (2000). 10985893 10.1006/gyno.2000.5918

